# Jomon genomics reveal cold adaptation in Upper Paleolithic hunter-gatherers of eastern Eurasia

**DOI:** 10.1126/sciadv.aea7469

**Published:** 2026-08-12

**Authors:** Yusuke Watanabe, Yoshiki Wakiyama, Daisuke Waku, Guido Valverde, Akio Tanino, Mizuki Hayashi, Izumi Naka, Yuka Nakamura, Tsubasa Suzuki, Kae Koganebuchi, Takashi Gakuhari, Takafumi Katsumura, Motoyuki Ogawa, Atsushi Toyoda, Pornlada Nuchnoi, Suwanna Chaorattanakawee, Hathairad Hananantachai, Jintana Patarapotikul, Soichiro Mizushima, Tomohito Nagaoka, Kazuaki Hirata, Minoru Yoneda, Midori Motoi, Hideo Toyoshima, Takafumi Maeda, Takayuki Nishimura, Tadayuki Masuyama, Yasuhiro Yamada, Ryuzaburo Takahashi, Masami Izuho, Jun Ohashi, NCBN Controls WGS Consortium, Hiroki Oota

**Affiliations:** ^1^ Department of Biological Sciences, Graduate School of Science, The University of Tokyo, Bunkyo-ku, Tokyo, Japan.; ^2^ Department of International Agricultural Development, Faculty of International Agriculture and Food Studies, Tokyo University of Agriculture, Setagaya-ku, Tokyo, Japan.; ^3^ Department of Anatomy, Kitasato University School of Medicine, Sagamihara, Kanagawa, Japan.; ^4^ Institute for Mummy Studies, Eurac Research, Bolzano, Italy.; ^5^ Department of Biosciences, Kitasato University School of Science, Sagamihara, Kanagawa, Japan.; ^6^ Center for the Study of Ancient Civilizations and Cultural Resources, College of Human and Social Sciences, Kanazawa University, Kanazawa, Japan.; ^7^ Department of Human Life Design and Science, Faculty of Design, Kyushu University, Minami-Ku, Fukuoka, Japan.; ^8^ Advanced Genomics Center, National Institute of Genetics, Mishima, Shizuoka, Japan.; ^9^ Department of Clinical Microscopy, Faculty of Medical Technology, Mahidol University, Bangkok, Thailand.; ^10^ Center for Research Innovation and Biomedical Informatics, Faculty of Medical Technology, Mahidol University, Bangkok, Thailand.; ^11^ Department of Parasitology and Entomology, Faculty of Public Health, Mahidol University, Bangkok, Thailand.; ^12^ Faculty of Tropical Medicine, Mahidol University, Bangkok, Thailand.; ^13^ Department of Anatomy, St. Marianna University School of Medicine, Kawasaki, Kanagawa, Japan.; ^14^ Aomori Public University, Aomori, Aomori, Japan.; ^15^ The University Museum, The University of Tokyo, Bunkyo-ku, Tokyo, Japan.; ^16^ Fukuoka Urasoe Clinic, Chuou-Ku, Fukuoka, Japan.; ^17^ Tahara City Board of Education, Tahara, Aichi, Japan.; ^18^ Department of History and Archaeology, Tokyo Metropolitan University, Hachioji, Tokyo, Japan.; ^19^ Waseda University, Shinjuku-ku, Tokyo, Japan.

## Abstract

How did *Homo sapiens* thrive in extreme environments during their dispersal across Eurasia? Unraveling adaptations that enabled human survival during the Last Glacial Maximum (LGM) remains critical. Here, we analyze 42 genomes from Holocene hunter-gatherers (Jomon) in the Japanese archipelago, revealing their deep ancestry from an isolated Upper Paleolithic (UP) East Eurasian lineage. We detect gene flow from northern UP Siberians into Jomon, reconciling long-standing debates between bioanthropology and archaeology regarding East Asian peopling. We find no Jomon-specific Denisovan ancestry, suggesting shared introgression before their LGM divergence from mainlanders. Furthermore, we find evidence consistent with cold adaptation in Jomon UP ancestry, highlighting diverse physiological pathways. Our study provides one of the first paleogenomic frameworks for investigating ancient adaptations in UP East Eurasians, revealing an evolutionary legacy from a colder past in present-day humans.

## INTRODUCTION

The dispersal of *Homo sapiens* into Southeast Asia ∼50,000 years ago marked a pivotal moment in East Eurasian prehistory (*1*, *2*). As groups expanded into northern latitudes, particularly during the challenging conditions of the Last Glacial Maximum (LGM) (*3*–*7*), they confronted environmental pressures. Survival in this extreme cold necessitated diverse biological adaptations—broadly categorized as metabolic, isolative, or hypothermic (*8*)—together with cultural innovations. However, the relative contributions of these drivers to human movement and persistence during the LGM remain unclear.

**Fig. 1.**
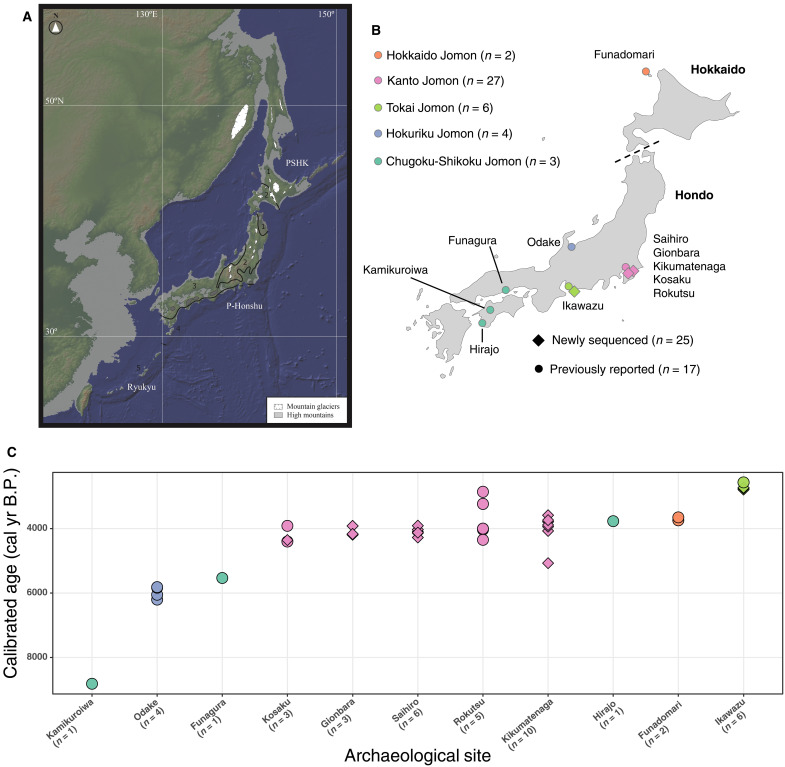
The map of the Japanese archipelago. (**A**) The hypothesized coastline during the LGM (100 m below the present sea level) and the distribution of vegetational areas during the LGM. (1) Open coniferous forest and grassland; (2) cool-temperate coniferous forest; (3) temperate pan-mixed forest; (4) warm-temperate deciduous broadleaf and evergreen broadleaf forests; (5) subtropical evergreen broadleaf forest and coniferous forest. Map was created using GeoMapApp (www.geomapapp.org)/CC BY. (**B** and **C**) Sampling locations and calibrated ages. Archaeological sites are marked with diamonds for samples sequenced in this study and circles for previously reported samples. The colors represent regions in the Japanese archipelago. In this study, regions except for Hokkaido are collectively referred to as Hondo. The current Hondo region was a single island known as the P-Honshu Island during the LGM, due to the lowering of sea levels, while Hokkaido was connected to continental Asia and known as the PSHK.

The Japanese archipelago, situated at the eastern edge of the Eurasian continent, provides a particularly informative setting for investigating the evolutionary history of East Eurasians. The Jomon—prehistoric hunter-gatherers inhabiting these islands from ∼16,000 to 3000 years ago—offer a valuable genomic resource. During the Late Pleistocene, before the emergence of Jomon pottery, the region corresponding to the present-day Japanese archipelago consisted of two major landmasses: Paleo-Honshu (P-Honshu) Island, which was nearly connected to the Korean Peninsula, and the Paleo-Sakhalin-Hokkaido-Kurile Peninsula (PSHK), which connected Hokkaido to the East Eurasian continent (Fig. 1A) (*9*, *10*). Archaeological evidence suggests that *H. sapiens* equipped with small flake-based tool assemblages first appeared on P-Honshu around 38,000 calibrated years before the present (cal yr B.P.) (*11*, *12*). At the end of the LGM, around 19,000 cal yr B.P. (*13*), rising temperatures and global sea level increase led to the submergence of these connections. This fragmented P-Honshu into the modern islands of Honshu, Shikoku, and Kyushu (collectively referred to here as “Hondo”) and isolated Hokkaido from the Eurasian mainland. This isolation suggests that the ancestors of the Jomon may have followed a distinct evolutionary trajectory from other Late Pleistocene East Eurasian populations.

Paleogenomic studies suggest that the Jomon represent a basal lineage in East Eurasia, with close affinity to Southeast Asian Hòabìnhian hunter-gatherers and deep divergence from present-day East/Northeast Asians and Native Americans (*6*, *7*). Genomic evidence also supports that present-day Japanese descend from admixture between the Jomon and later agricultural people from the mainland (*14*). However, whether the Jomon directly descend from Upper Paleolithic (UP) hunter-gatherers who endured glacial conditions remains unclear. Here, we analyze the genomes of 42 Jomon individuals to trace the emergence of cold-adaptive traits in UP East Eurasians and to clarify the Jomon’s role in the broader evolutionary history of eastern Eurasia.

## RESULTS

### Basal since the UP

We analyzed 42 Jomon genomes (25 sequenced in this study and 17 published), including three high-coverage (∼30×) individuals from across the Japanese archipelago (Fig. 1, B and C). To enhance data quality, we imputed genotypes using a reference panel of 9290 modern Japanese individuals, achieving high imputation accuracy (fig. S2 and text S1). Because present-day Japanese retain substantial Jomon ancestry, this panel serves as an ancestry-matched reference for Jomon genomes. Given the deep divergence of the Jomon lineage within East Eurasia, this ancestry matching was particularly important for accurate imputation. Sensitivity analyses excluding modern Japanese genomes from the reference panel resulted in reduced imputation accuracy and distorted demographic inference (fig. S2 and text S1), demonstrating that non–ancestry-matched reference panels can bias downstream demographic analyses of other deeply diverged East Eurasian ancient genomes.

**Fig. 2.**
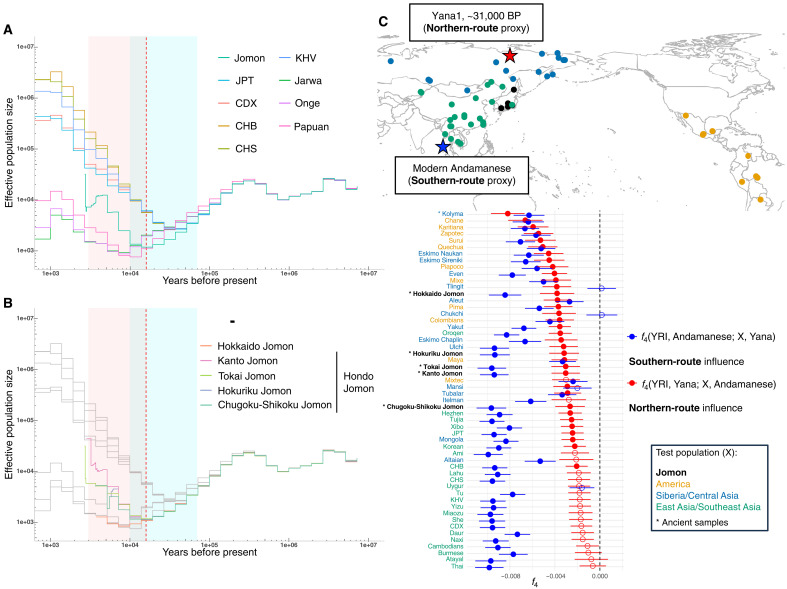
Demographic analyses of Holocene hunter-gatherers in the Japanese archipelago. (**A** and **B**) Effective population size change within the Jomon lineage and East Eurasians. We estimated population size using coalescence rate calculated from Relate-based genome-wide genealogies of 40 Jomon individuals who were subjected to radiocarbon dating, 1KG East Eurasians [Japanese in Tokyo, Japan (JPT), Han Chinese in Beijing, China (CHB), Southern Han Chinese (CHS), Chinese Dai in Xishuangbanna, China (CDX), and Kinh in Ho Chi Minh City, Vietnam (KHV)], Simons Genome Diversity Project Papuan and Andamanese (Jarwa and Onge) from Mondal *et al.* (*97*). (A) The overall population size change of the Jomon lineage. (B) The population size changes within regional Jomon populations. Light blue shading, the Last Glacial period (10,000 to 70,000 years before present); red shading, the Jomon period (3000 to 16,000 years before present). (**C**) *f*_4_ statistics assessing genetic influences from the northern and southern routes on Jomon and other populations. Left: Geographical distribution of modern and ancient populations tested. Right: Corresponding *f*_4_ values for each population. YRI denotes Yoruba in Ibadan, Nigeria. The red points represent *f*_4_(YRI, Yana; X, Andamanese), indicating northern-route influence, while the blue points represent *f*_4_(YRI, Andamanese; X, Yana), indicating southern-route influence. The *x* axis shows *f*_4_ values, and the *y* axis lists test populations (X), including Jomon, EASs/Southeast Asians, Siberians/Central Asians, and Americans from 1KG, Human Genome Diversity Project (HGDP), SGDP, and Korean Personal Genome Project (KPGP). Ancient samples including Jomon and Kolyma1, a representative ancient Siberian genome, are marked with an asterisk (*). Open symbols denote statistics with |*Z*| < 2, while filled symbols denote |*Z*| ≥ 2. SEs are block-jackknife SEs as implemented in ADMIXTOOLS (qpDstat).

We then estimated the demographic history of the Jomon lineage using Relate (*15*), incorporating selected modern East Eurasian populations for comparison. Papuans and Önge/Jarwa (Andamanese; a sister group of Hòabìnhian foragers) represent early-diverging southern lineages (*6*), while East Asian (EAS) populations from the 1000 Genomes Project (1KG) represent groups that split later. These comparisons contextualize the Jomon within the broader East Eurasian demographic landscape. Relate analyses yielded three notable findings: (i) The Jomon lineage diverged from continental East Eurasians ∼27,000 to 19,000 years ago during the UP (fig. S8); (ii) the population size of the Jomon lineage experienced a notable decline during the UP, followed by a recovery at the onset of the Jomon period (Fig. 2A); (iii) the Hokkaido Jomon lineage diverged from the Hondo Jomon lineage ∼10,000 to 8800 years ago, initiating a distinct population history during the Jomon period (Fig. 2B and fig. S9).

The UP occupation in the archipelago began around 38,000 cal yr B.P. (*11*) and continued until about 15,000 cal yr B.P. on P-Honshu, extending from 15,000 to 12,000 cal yr B.P. on Hokkaido, south of PSHK (*16*, *17*). The UP in both regions is divided into three substages—early, middle, and late—characterized by lithic reduction patterns, toolkit compositions, stone-tool types, and site dates. Notably, the middle UP, spanning ∼30,000 to 20,000 cal yr B.P., shows heightened mobility with blade-based backed point assemblages in P-Honshu (*12*, *16*, *18*, *19*). The estimated divergence between the Jomon lineage and continental East Eurasians (∼27,000 to 19,000 years ago; fig. S8) corresponds to the middle-to-late UP, supporting the view that the Jomon lineage emerged from an isolated UP East Eurasian population. Given the changes observed in cultural evidence between the early and the middle UP (*11*, *16*, *18*), together with the timing of the estimated Jomon–continental East Eurasian split (fig. S8), it is possible that reduced connectivity between populations in the Japanese archipelago and continental East Eurasia just before or during the LGM resulted in a divergence, ultimately giving rise to the later Jomon people.

The divergence times between the Jomon lineage and Papuans/Andamanese were also estimated to be between 27,000 and 19,000 years ago (fig. S8), possibly reflecting the rapid dispersal and then subsequent isolation of *H. sapiens* from Eurasia during the abrupt climatic cooling of the LGM, as seen in peripheral regions such as the Japanese archipelago, Sundaland, and the Andaman Islands. The decrease in frequency of cultural occupation preceding the Jomon period (*20*) closely aligns with our findings of a population size decline during this time (Fig. 2). After the LGM, surviving UP hunter-gatherers in the Japanese archipelago contributed to the emergence of the Jomon culture, which thrived in the warmer post–glacial period and is known for its distinctive pottery (*21*). The population size increase began much later in other peripheral East Eurasians such as Papuans and Andamanese than in the Jomon lineage, with population size stagnation until ∼10,000 years ago (Fig. 2A). This earlier growth in the Jomon lineage may have been facilitated by cultural changes associated with the rise of the Jomon tradition, together with ecological and demographic factors, as suggested by contemporaneous archaeological evidence (text S2).

The estimated Holocene divergence between the Hokkaido and Hondo Jomon lineages (fig. S9) suggests that the Hokkaido Jomon shared a recent common ancestry with the Hondo Jomon, rather than representing a direct continuation of the UP inhabitants of Hokkaido. Together with the phylogenetic patterns observed in fig. S3 (text S3), these observations strongly support a “replacement model” in which the UP Hokkaido population was replaced by Jomon immigrants from Hondo during the Holocene. Under this model, the UP Hokkaido population appears to be a “ghost population”—that is, a group that left little to no genetic legacy in later Jomon individuals. This genetic discontinuity is further supported by archaeological evidence: (i) a decline in cultural occupation around 15,000 years ago (*10*, *18*, *22*, *23*) and (ii) abrupt changes in the lithic reduction system, along with the introduction of cultural traits such as pottery production (*17*, *18*, *20*, *21*). Although the specific migration routes taken by UP populations from the continent into the PSHK and P-Honshu remain uncertain, these lines of evidence point to a population replacement before, or during, the early Holocene in Hokkaido.

### Gene flow from northern UP populations and perspectives on the First Americans

Our major interests concern the routes by which *H. sapiens* spread into eastern Eurasia. On the basis of archaeological evidence, it has been proposed that, after leaving Africa, *H. sapiens* reached eastern Eurasia via two distinct routes: one north of the Himalayan Mountains through Siberia (northern-route) and another south of the Himalayan Mountains through the Indian subcontinent and into Southeast Asia (southern-route). Although blade-based technologies resembling those of northern UP populations have been found in the Japanese archipelago (36,000 to 24,000 cal yr B.P.), suggesting cultural ties (*10*, *12*, *24*–*27*), genetic studies have provided little evidence for northern-route ancestry (*28*).

To test for genetic input from northern UP populations into the Jomon, we compared shared drift with northern- and southern-route proxy populations (*7*, *29*) (Fig. 2C and table S4). Jomon individuals, similar to other East/Southeast Asians, showed greater affinity with Andamanese (i.e., the southern proxy), supporting a southern origin of their UP ancestry. The magnitude of this affinity was comparable to that observed in present-day East and Southeast Asians. However, genetic affinity between the Jomon and northern proxy was slightly higher than that observed in other EASs at the same latitude and was comparable to or slightly lower than that seen in modern Siberians and Native Americans. This consistent signal, not previously reported, suggests that subtle gene flow from northern UP populations may have occurred into the Jomon ancestry, possibly accompanying the spread of blade-based technologies.

The signal appears particularly in the Hokkaido Jomon, raising the intriguing possibility of regional variation in contact with northern-route populations. We speculate that the UP population of Hokkaido—identified as a ghost population—carried northern UP ancestry and that the higher northern affinity observed in the Hokkaido Jomon relative to other Jomon groups reflects limited but detectable admixture with its remnants during the early Holocene. Archaeological evidence from the southern PSHK during the Middle Upper Paleolithic (MUP) and Late Upper Paleolithic (LUP) suggests that local UP foragers had closer cultural connections with Siberian groups than with those in Honshu (*10*, *18*, *23*–*25*). Although cultural connections do not necessarily imply gene flow, this archaeological pattern is broadly consistent with the scenario outlined above. While this pattern is suggestive, we emphasize that it does not reflect an exceptionally strong Yana affinity in the Hokkaido Jomon. As our additional *f*_4_(YRI, Yana; Hokkaido Jomon, other Jomon/EASs) tests show (table S5), absolute differences in Yana-related drift among Jomon and other EASs are modest; thus, we interpret the Hokkaido Jomon as exhibiting only a relatively elevated tendency within the broader EAS context, rather than a distinct or exceptional signal. Nevertheless, the underlying demographic process remains uncertain, and the observed signal may alternatively reflect more recent gene flow from northern populations rather than persistence of northern UP-related ancestry in Hokkaido.

Our findings provide insights into the initial peopling of the Americas. A previous study has rejected the “Out-of-Japan” hypothesis, citing the lack of genetic affinity between Hokkaido Jomon and Native American populations (*30*). Under our replacement model, however, the Hokkaido Jomon are not appropriate proxies for the earlier UP inhabitants of Hokkaido: They represent a Holocene population derived from Honshu, not a continuation of the Pleistocene Hokkaido lineage. Thus, prior negative results speak only to the Holocene Jomon layer and do not exclude potential links involving the earlier UP Hokkaido population.

Our results point to a population turnover in southern PSHK, indicating that the earlier UP inhabitants represent a distinct ghost population unrelated to the later Jomon. If the elevated northern affinity observed in the Hokkaido Jomon reflects residual ancestry inherited from this ghost population, then the UP inhabitants of Hokkaido may have carried genetic components related to northern-route UP populations. These observations, together with archaeological evidence of cultural connections, leave open the possibility that UP populations in Hokkaido contributed to the initial dispersal into North America south of the continental ice sheet (*22*, *23*). Although we do not claim evidence for a UP Hokkaido contribution to the First Americans, our findings reopen this question by showing that the relevant population layer has not yet been genetically sampled. Resolving this issue requires genome-wide data from UP Hokkaido individuals to test symmetry with Ancient Paleo-Siberians and early First American–related groups. Until these data are available, the role of UP Hokkaido in broader northern Eurasian population dynamics remains an important open question.

### Archaic segments shared in eastern-edge Eurasians

The presence of Denisovan ancestry in modern human genomes reflects multiple, lineage-specific introgression events (*31*, *32*). By tracing archaic segments in the Jomon—an early diverging lineage in East Eurasia—we provide evidence that the Jomon and mainland EASs share northern Denisovan ancestry, distinct from the Denisovan lineage predominant in Papuans. We infer that imputation—prone to missing rare Denisovan alleles—artificially lowers the apparent number of archaic segments in the Jomon genomes relative to whole-genome sequencing. Therefore, rather than a quantitative comparison for Denisovan segments, we focused on differences in the distribution of matches to Altai Denisovan among populations.

**Fig. 3.**
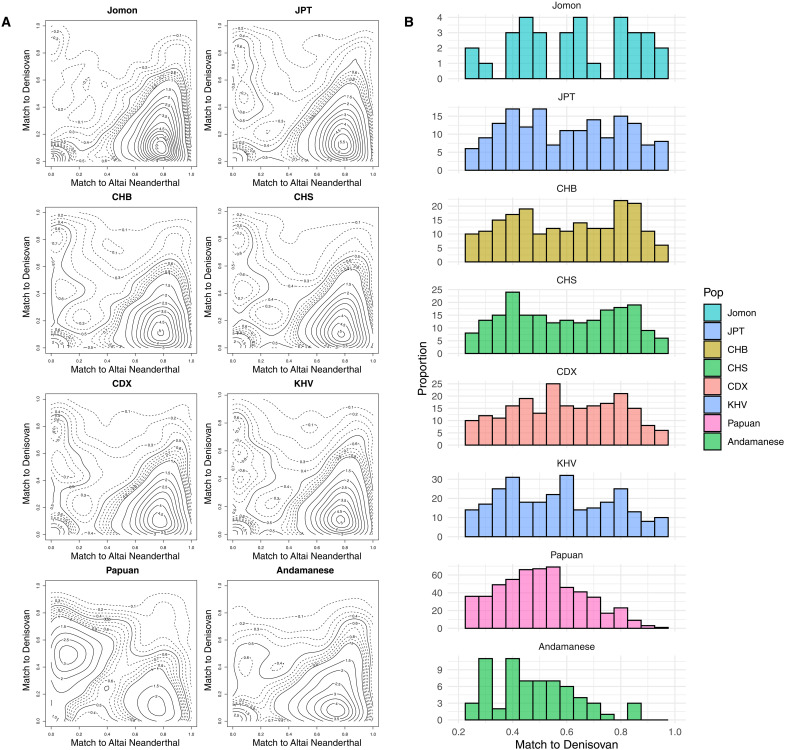
Distribution of match rates to Denisovan/Neanderthal genomes for detected archaic segments in the Jomon individuals and East Eurasians. We inferred archaic segments of 40 Jomon individuals, 1KG East Eurasians (JPT, CHB, CHS, CDX, and KHV), SGDP Papuans, and Andamanese from Mondal *et al.* (*97*) by SPrime software, a reference-free detection method, using 1KG Yoruba (YRI) individuals as a nonadmixed population. The match rate is calculated by the number of matches to the given archaic genome divided by the number of putative archaic alleles in the focal segment. (**A**) Contour density plots of match rate of archaic segments to the Altai Neanderthal/Denisovan. (**B**) Histograms of Match to Denisovan for the archaic segments with a match to Neanderthal of <0.2 and a match to Denisovan of >0.2, which we deemed as derived from the Denisovan lineage.

Although the Jomon and 1KG EAS showed similar distributions of Denisovan-matching segments, Papuan and Andamanese populations differed significantly (permutation test, *P* < 0.01; Fig. 3 and tables S6 and S7). This suggests that Denisovan segments shared by the Jomon and other EASs entered their common ancestral gene pool after diverging from Papuans and Andamanese but before the Jomon mainland split. This interpretation agrees with previous findings that EASs carry Denisovan-derived sequences (termed “D0”) more similar to the Altai Denisovan genome than those found in Papuans (“D1”), suggesting multiple region-specific Denisovan introgression events (*32*). This sharing pattern supports the interpretation that these D0-related segments were already present in the basal gene pool of eastern-edge Eurasians, including the Jomon lineage. Our divergence estimation (fig. S8) indicates that D0-related introgression likely occurred before or during the LGM. The recent identification of Denisovans using paleoproteomics (*33*–*36*) further supports their presence along the eastern margins of continental Asia. These findings support a model in which *H. sapiens* encountered D0 Denisovans primarily in Northeast rather than Southeast Asia.

To further evaluate the presence of Denisovan ancestry using a complementary source-aware framework, we applied IBDmix (*37*) to our imputed diploid Jomon genomes. Comparison with 1KG EASs [excluding Japanese in Tokyo, Japan (JPT)] revealed five Jomon-specific Denisovan segments, a number comparable to those identified in other EASs [Han Chinese in Beijing, China (CHB), 9; Southern Han Chinese (CHS), 3; Kinh in Ho Chi Minh City, Vietnam (KHV), 9; Chinese Dai in Xishuangbanna, China (CDX), 9]. By contrast, applying the same procedure to Papuans yielded 55 population-specific segments, consistent with their substantially higher Denisovan ancestry. A recent study by Yang *et al.* (*38*), using a statistical framework different from the one used here, also suggested that the Jomon may carry lower absolute amounts of Denisovan ancestry than other EASs. Such a reduced amount is compatible with our finding that, although Denisovan segments are present in the Jomon, there is no evidence for an additional Jomon-specific Denisovan introgression pulse beyond the D0-related ancestry shared across EASs.

### Cold adaptation in the UP hunter-gatherers

The Jomon exhibited markedly different frequencies from present-day EASs in phenotype-related alleles that show positive selection in the latter (Table 1). In particular, alleles that reached high frequencies in present-day EASs, including variants associated with hair and dental morphology (*EDAR*) (*39*, *40*), alcohol metabolism (*ADH1B* and *ALDH2*) (*41*, *42*), pigmentation (*OCA2* and *MC1R*) (*43*–*45*), and earwax type (*ABCC11*) (*46*), were generally less frequent in the Jomon, consistent with their ancestry deriving from a UP East Eurasian lineage that diverged from continental populations before the rise of many characteristic EAS adaptive variants. In contrast, the Jomon carried notably higher frequencies of cold adaptation variants, including the *UCP1* haplotype that boosts nonshivering thermogenesis (NST) (*47*, *48*) and the *TRPM8*-derived allele linked to cold sensation (*49*)—both alleles whose frequencies increase toward lower temperature (*47*–*49*) (fig. S10). Although we do not treat this replicated allele frequency–temperature correlation plot (fig. S10) as independent evidence of cold adaptation, if the assumptions underlying prior studies hold, the position of the Jomon in the plot would nevertheless be consistent with a history of cold exposure.

**Table 1.** Allele frequencies in the Jomon associated with phenotypes predominant in East Eurasians. The alleles denoted in bold in the ancestral/derived columns are associated with the EAS characteristic phenotypes.

Phenotype	Gene	Chromosome	Position	rs ID	Ancestral	Derived	Allele frequency associated with phenotype				Reference
							Jomon	EAS	EUR	AFR	
Thicker hair	*EDAR*	2	108897145	rs3827760	A	**G**	0	0.87	0.01	0.003	Fujimoto *et al.* (*39*)
Shovel-shaped incisors											Kimura *et al.* (*40*)
Alcohol intolerance	*ADH1B*	4	99318162	rs1229984	C	**T**	0	0.7	0.029	0.0015	Edenberg and Borson (*41*)
	*ALDH2*	12	111803962	rs671	G	**A**	0	0.17	0	0	Harada *et al.* (*42*)
Light skin color	*OCA2*	15	27951891	rs1800414	T	**C**	0.48	0.6	0	0.0008	Yang *et al.* (*43*)
Iris color											Rawofi *et al.* (*44*)
Freckles	*MC1R*	16	89919532	rs2228479	G	**A**	0	0.29	0.069	0.0038	Yamaguchi *et al.* (*45*)
Light skin color		16	89919746	rs885479	G	**A**	0.35	0.61	0.07	0.0068	
Dry earwax	*ABCC11*	16	48224287	rs17822931	C	**T**	0.24	0.78	0.14	0.012	Yoshiura *et al.* (*46*)
Nonshivering thermogenesis (NST)	*UCP1*	4	140554683	rs3113195	A	**G**	0.67	0.47	0.67	0.24	Hancock *et al.* (*47*) Nishimura *et al.* (*48*)
		4	140563980	rs12502572	A	**G**	0.67	0.48	0.67	0.24	
		4	140572807	rs1800592	**T**	C	0.72	0.53	0.76	0.27	
		4	140580184	rs4956451	**A**	G	0.72	0.53	0.67	0.1	
Cold sensation	*TRPM8*	2	233916448	rs10166942	C	**T**	0.74	0.37	0.83	0.1	Key *et al.* (*49*)

**Fig. 4.**
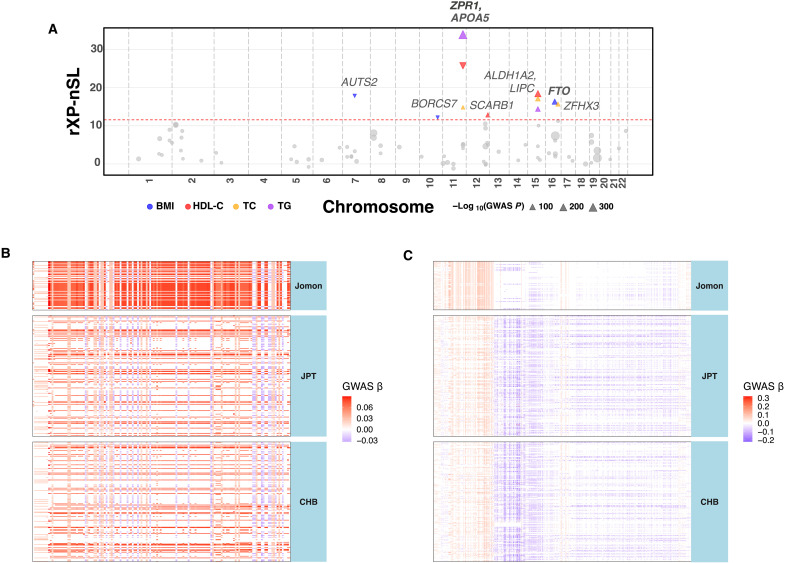
Selective sweep in genetic regions associated with cold adaptation. (**A**) rXP-nSL values for trait-associated regions which were detected by previous genome-wide association studies for BMI, high-density lipoprotein cholesterol (HDL-C), low-density lipoprotein cholesterol, total cholesterol (TC), and TGs. rXP-nSL values are plotted against the physical positions on the chromosomes. We only illustrated regions with an rXP-nSL of >0 that exhibit selective sweep signals for the Jomon rather than continental East Eurasians. The 95th percentile of the empirical distribution is shown as a red dashed horizontal line and was used as the empirical significance threshold. Triangles mark regions with significant rXP-nSL values, and their orientation (upward or downward) signifies whether the selected haplotype in the Jomon elevates or reduces the focal traits. (**B** and **C**) Haplotype structures of the *FTO* and *ZPR1-APOA5* gene regions among the Jomon, CHB, and JPT. Each column represents a trait-associated SNP site and each row represents a phased haplotype. The color of each cell represents the direction of effect for traits, and color intensity represents the β value for each effect allele identified by previous genome-wide association studies. In the *FTO* region, we observed the accumulation of alleles associated with increased BMI in the Jomon individuals compared to CHB and JPT. Conversely, in the *ZPR1-APOA5* region, the Jomon individuals showed less accumulation of alleles associated with decreased TG, which were commonly observed in CHB and JPT.

To further explore the phenotypes acquired through positive natural selection, we conducted a genome-wide selective sweep scan using the regional cross-population number of segregating sites by length (rXP-nSL), a statistic that combines XP-nSL scores (*50*) and genome-wide association study (GWAS) summary statistics (figs. S11 and S12). Because no Jomon-specific GWAS is available, assessing the evolutionary direction of allelic changes requires extrapolating effect orientations from present-day Japanese GWAS. To avoid errors that arise when relying on a single representative single-nucleotide polymorphism (SNP)—whose effect direction may differ across populations due to linkage disequilibrium (LD) differences—we evaluate selection at the level of entire GWAS loci by aggregating XP-nSL signals and trait-associated effects across all SNPs in each region. This locus-wide framework provides a more reliable basis for inferring both the presence and the direction of selection in the Jomon. We identified several genetic regions that underwent positive selection in the Jomon lineage, including loci involved in body mass index (BMI) and lipid metabolism (Fig. 4A and fig. S12). Notable regions included the *FTO* and *ZPR1-APOA5* gene loci, which are linked to increased BMI and elevated triglyceride (TG) levels, respectively (*51*, *52*). The Jomon exhibited haplotypes in the *FTO* and *ZPR1-APOA5* regions associated with higher BMI and TG levels than in present-day CHB and JPT (Fig. 4, B and C). The 140 phenotype-associated SNPs in the *FTO* region (Fig. 4B) include rs8047395, which has an XP-nSL of 2.54 (ranking in the top 1% of 13 million SNPs; fig. S13). The 1019 phenotype-associated SNPs in the *ZPR1-APOA5* region (Fig. 4C) include rs1729410, which has an XP-nSL of 4.18 (ranking in the top 0.005% of 13 million SNPs; fig. S13). The Jomon carry the highest known frequencies of variants most strongly linked to elevated BMI and TG (Table 2). Together, these observations support positive selection acting on loci associated with BMI and lipid metabolism in the Jomon lineage. Sensitivity analyses further confirmed that these signals are robust to alternative LD-clumping parameters and to variation in GWAS effect-size panels: Using an independent large-scale Taiwanese GWAS (*53*) in place of the Japanese cohort, both *APOA5* and *FTO* again emerged among the top candidates across all settings (tables S8 and S9).

**Table 2.** Derived allele frequencies in rs11642015 in the *FTO* region and rs662799 in the *APOA5* region, which exhibited the lowest *P* values in the previous GWAS for their respective phenotypes (BMI and TG). For the Jomon, we presented allele frequencies in 40 imputed genomes, as well as allele frequencies in seven individuals with a depth greater than 5×, as genotype called by bcftools.

Phenotype	Gene	Chromosome	Position	rs ID	Ancestral	Derived	Allele frequency associated with phenotype								GWAS *P* value
							Jomon (imputed, *n* = 40)	Jomon (≧5× depth, called with bcftools, *n* = 7)	1KG EAS	1KG EUR	1KG AFR	SGDP Papuan	SGDP Central Asia/ Siberia	SGDP America	
Higher BMI	*FTO*	16	53768582	rs11642015	C	**T**	**0.73**	0.57	0.17	0.43	0.06	0.033	0.19	0.09	4.9 × 10^−57^^*^
Higher TG level	*APOA5*	11	116792991	rs662799	A	**G**	**0.89**	0.79	0.29	0.08	0.12	0.27	0.33	0.32	<1.0 × 10^−320^†

* Akiyama *et al.* (*51*).

† Kanai *et al.* (*52*).

Regarding the molecular basis for these traits, experimental evidence supports the role of *FTO* and *APOA5* in brown adipose tissue (BAT) activity, which is central to NST. The *FTO* gene harbors the rs1421085 T > C allele, which has been shown in both in vivo and in vitro experiments to enhance NST. Introducing the rs1421085 T > C allele into mice has been shown to enhance thermogenesis by up-regulating *Fto* expression and promoting the maintenance of body temperature under cold exposure (*54*), indicating that the rs1421085 C allele is a functional variant promoting brown-fat thermogenesis. The Jomon exhibit a high frequency of the rs1421085 C allele (0.725), substantially higher than that observed in present-day EAS (0.168), European (0.43), and African populations (0.056). The rs1421085 C allele was encompassed within the haplotype subject to positive natural selection in the Jomon (Fig. 4B), suggesting that this allele was positively selected in the Jomon to enhance cold adaptation.

Similarly, *APOA5* plays a crucial role in lipid metabolism, particularly in the utilization of TG for heat production in BAT. NST is primarily produced by BAT in humans through the utilization of free fatty acids derived from TG. The accumulation of apolipoprotein A5 (apoA5) protein within adipose cells accelerates intracellular TG metabolism, increasing the expression of *UCP1*, a gene vital for NST (*55*). Therefore, the prevalence of *APOA5* haplotypes in the Jomon likely enabled a higher supply of TG from the bloodstream to BAT, facilitating more efficient heat generation. A signal of positive natural selection for the haplotype contributing to the elevated TG levels of the Jomon was observed not only in the *APOA5* gene but also in the *ALDH1A2-LIPC* gene region on chromosome 15 (Fig. 4A), suggesting that positive natural selection acted on multiple loci associated with elevated TG levels. In the case of the Jomon, these genetic adaptations likely enabled not only the accumulation of fat but also its efficient burning in BAT to maintain body temperature, thereby optimizing energy use in extreme cold. Although the functional links among *UCP1*, *APOA5*, and *FTO* rely primarily on findings from prior physiological and molecular studies, our analyses establish the evolutionary and genetic signatures acting on these loci. Together, these lines of evidence support the idea that the UP hunter-gatherers of East Eurasia who were ancestors of the Jomon adapted to cold environments through genetic changes that enhanced NST via *UCP1*, *APOA5*, and *FTO*. NST, facilitated by BAT, allowed the Jomon to maintain body heat more efficiently than through shivering alone.

Another line of evidence for cold adaptation in the Jomon lineage comes from a notable similarity with the Inuit, a population well known for their adaptations to cold environments. In the Inuit, these adaptations are strongly linked to variants in the *FADS* gene family, which are associated with the metabolism of long-chain polyunsaturated fatty acids (PUFAs) derived from a high-fat, marine-based diet (*56*). At the *FADS* locus, the Jomon show allele frequencies significantly closer to the Inuit than to EASs (fig. S14 and table S10), as indicated by a cluster of strongly negative *f*_4_(CEU, GI; Jomon, CHB) values ranking among the most extreme signals in the genome-wide empirical distribution. This pattern suggests a locus-specific convergence driven by similar dietary pressures. This genetic signal is supported by archaeological evidence: Traces of marine and freshwater resource use have been found in Jomon pottery dated to ∼15,000 to 11,800 years ago across the Japanese archipelago (*57*). These findings indicate that Incipient Jomon foragers, representing the earliest stage of the Jomon cultural tradition, heavily relied on aquatic resources at the end of the glacial period. Together, the genetic and archaeological evidence suggests that the Jomon lineage developed metabolic adaptations to a high-fat, high-protein diet, supporting cold tolerance via NST—a strategy convergent with that of the Inuit.

We identified polygenic signatures of cold adaptation in the Jomon lineage, involving multiple genes associated with NST, dietary metabolism, and cold sensation. These adaptations may have originated not during the Holocene but earlier—specifically during the LGM—when the ancestors of the Jomon, as UP East Eurasians, faced extreme cold following their northward migration. To explore this hypothesis, we compared the allele frequencies of cold-adaptive variants between the Jomon and other modern and ancient populations (table S11). Notably, derived alleles in *APOA5* and *TRPM8* were found at high frequencies only in the Jomon but not in the Andamanese or in ancient Siberian (*29*) and North American individuals (*28*, *58*, *59*). In contrast, *FTO*, *UCP1*, and *FADS2* variants were shared across both groups. These patterns are supported by both allele frequency and haplotype network analyses (figs. S15 and S16). We observed higher selection coefficients (*s*) and a more rapid increase in the frequency of the cold-adaptive allele in the *APOA5* gene during the Last Glacial period compared to the post–Last Glacial period (fig. S17). These results suggest that cold adaptation in UP hunter-gatherers was particularly critical during the LGM. Robustness assessments using 18 TG-associated SNPs (GWAS *P* < 1.0 × 10^−200^) across *APOA5* yielded nearly identical selection coefficients (table S12)—again highlighting a sharp increase during the Last Glacial period—demonstrating that the signal reflects coordinated selection across the locus rather than dependence on a single SNP.

These comparative results support our central hypothesis that cold adaptation in UP East Eurasians followed a gene-specific and regionally distinct trajectory. The Jomon likely descend from southern-route migrants who initially settled in warmer Southeast Asian climates (Fig. 2C) (*6*). Within this context, we propose the following evolutionary scenario: Several derived alleles associated with cold adaptation—particularly in *FTO*, *FADS2*, and *UCP1*—were already present at moderate to high frequencies in the common ancestors of both the Jomon and Hòabìnhian hunter-gatherers, as suggested by their similarly high frequencies in the Jomon and Andamanese. These variants, likely beneficial for energy storage and metabolic efficiency, may have conferred adaptive potential even before the migration into colder climates. Among the descendants of this UP population, a subgroup migrated northward through East Asia and eventually gave rise to the Jomon lineage. As these groups moved into colder environments during the LGM, they experienced cold-related ecological pressures that likely triggered further adaptive responses. In this context, *APOA5* and *TRPM8*, genes involved in TG transport to BAT and cold sensation, respectively, underwent lineage-specific selection, with their derived alleles rising to high frequency exclusively in the UP ancestry of the Jomon people. This suggests that cold adaptation in UP East Eurasians was not a single event or uniform process but rather a stepwise accumulation of adaptive alleles.

Understanding evolutionary processes provides valuable insights into how ancient genetic adaptations continue to influence present-day phenotypes. Notably, recent studies have shown that higher proportions of Jomon ancestry are associated with increased obesity rates in present-day Japanese (*60*, *61*). These phenotypes may partly reflect Jomon-derived cold-adaptive variants that may have facilitated survival under the extreme climatic conditions of the LGM through more efficient energy storage and utilization but that may increase susceptibility to adiposity under modern lifestyles—consistent with the “thrifty gene” hypothesis (*62*). By linking ancestral adaptations to modern phenotypic variation within an evolutionary framework, our study contributes to a deeper understanding of modern disease susceptibility and highlights the potential of paleogenomics to inform public health strategies (*63*).

## DISCUSSION

Our analysis of Jomon genomes lends support to their descent from populations that diverged from continental UP groups and settled in the Japanese archipelago ∼27,000 to 19,000 years ago. Following the post-LGM sea level rise (∼19,000 cal yr B.P.), geographic isolation facilitated the long-term genetic continuity of this lineage. As a result, the Jomon genome retains distinct UP characteristics for more than 10,000 years, serving as a genetic archive of UP East Eurasia. At the same time, our results highlight part of the spatiotemporal diversity of the Jomon, as illustrated by the distinct Holocene population history of Jomon groups in Hokkaido relative to Hondo.

We present multiple lines of evidence for cold adaptation during this period. Given the marked differences in diet and environment between past and present, interpretations of ancient physiological phenotypes remain largely speculative—especially in the Japanese archipelago, where poor faunal preservation has limited dietary reconstruction. By focusing on UP ancestry preserved in Jomon genomes, this study provides a pioneering paleogenomic framework for reconstructing physiological traits, dietary adaptations, and environmental responses in UP East Eurasians. Our findings illuminate how ancient populations adapted to extreme environments, and how these adaptations may continue to shape present-day variation in health-related traits, highlighting the broader relevance of paleogenomics to human biology and public health.

## MATERIALS AND METHODS

### Archaeological samples

In this study, we sequenced DNA from 25 human remains from five sites assigned to the Middle, the Late, and the Final Jomon periods [the Middle, 5400 to 4400 cal yr B.P.; the Late, 4400 to 3300 cal yr B.P.; the Final, 3300 to 2300 cal yr B.P. (*64*)].

#### Shell mounds in Ichihara City

We examined the human remains from three shell mounds, Kikumatenaga, Gionbara, and Saihiro, located in Ichihara City, Chiba Prefecture, overlooking Tokyo Bay. Excavations at the Kikumatenaga shell mound were conducted between 1972 and 1995, unearthing approximately 80 Jomon individuals. Radiocarbon dating placed these individuals in 5280 to 3490 cal yr B.P. (table S1) (*65*). Similarly, the Gionbara shell mound underwent five surveys between 1977 and 1995, yielding 112 human skeletal remains from the shell layer of the Late Jomon period. Archaeological analysis suggests that both sites were used from the Late Jomon period to the Final Jomon period, with individuals dated to between 4295 and 3826 cal yr B.P. (table S1) (*65*). In addition, the Saihiro shell mound underwent seven surveys between 1972 and 1987 as part of road construction, unearthing 72 human skeletons. Archaeological analysis of these remains indicated usage of this site from the end of the Middle Jomon period to the early half of the Final Jomon period, with individuals dated to between 4404 and 3779 cal yr B.P. (table S1) (*65*).

Petrous bones from individuals excavated at the shell mounds in Ichihara City are curated at the Department of Anatomy, St. Marianna University School of Medicine. Sampling was conducted with permission from the Department of Anatomy, St. Marianna University School of Medicine and the Ichihara City Board of Education.

#### Kosaku shell mound in Funabashi City

Situated in Funabashi City, Chiba Prefecture, overlooking Tokyo Bay, the Kosaku shell mound has been under excavation since 1926, resulting in the collection of approximately 100 human skeletons from this site. Among them, the subject of this study, KS007, is an infant found cradled in the chest of a woman in her prime. On the basis of pottery chronology, this site is estimated to date to the Late Jomon period. Radiocarbon dating of KS007 places its age at 4420 to 4291 cal yr B.P. (95.4%), consistent with the archaeological contexts of this layer.

Petrous bone from the individual excavated at the Kosaku shell mound is curated at the Department of Anatomy, St. Marianna University School of Medicine. Sampling was conducted with permission from the Department of Anatomy, St. Marianna University School of Medicine.

#### Ikawazu shell mound in Tahara City

Situated in Tahara City, Aichi Prefecture, near the center of the Japanese main island (Honshu), the Ikawazu shell mound has been a focal point of excavation since its initial exploration in 1918, yielding over 100 human bones accompanied by Jomon pottery. On the basis of pottery chronology, this site is classified as the Late to Final Jomon period. The individuals examined in this study were found in association with the type of Gokan-no-mori pottery, characterized by the absence of evidence for rice cultivation. Our analysis confirmed their affiliation with the Jomon period, marked by typical Jomon cultural traits. Radiocarbon dating of five individuals showed a range of 2848 to 2712 cal yr B.P. (table S1), with four of these individuals previously dated by Yamada (*66*).

Petrous bones from individuals excavated at the Ikawazu shell mound are curated at the Tahara City Board of Education. Sampling was conducted with permission from the Tahara City Board of Education.

### Gelatin extraction and radiocarbon dating

Collagen was extracted from each sample, and purified gelatin was obtained using protocols from previous studies (*67*, *68*). Radiocarbon dating was carried out using a compact accelerator mass spectrometer at The University Museum, The University of Tokyo. Calibrated radiocarbon ages were calculated by referencing the IntCal20 and Marine20 datasets (*69*, *70*) using OxCal 4.4 software (*71*).

Given the shell mound origin of our samples, predominantly sourced from marine resources, stable isotope ratios of carbon and nitrogen were measured from extracted gelatin. This allowed for correction of the marine reservoir effect, accounting for variations in the intake ratio of marine fish and shells. A regional correction value (Δ*R*) specific to Tokyo Bay (−98 ± 37 years) was applied to Marine20 based on analysis of a shell specimen collected in 1882 CE (534 ± 36 B.P.) (*72*).

For Ikawazu Jomon, the contribution of amino acids from marine resources was estimated by considering the spectrum between terrestrial herbivores and marine fish. This analysis suggested that ∼50% of the diet at Ikawazu Jomon was derived from marine sources.

### DNA extraction

Of the 25 individuals analyzed in this study, 18 were selected from individuals previously screened on the basis of their endogenous DNA percentage exceeding 10%. For these 18 individuals, sequencing libraries were prepared in the present study from DNA extracted previously (*65*). The remaining seven individuals underwent DNA extraction and library preparation in the present study.

DNA extraction and library preparation were performed in a dedicated clean room exclusively designed for ancient DNA analyses, installed in the Department of Anatomy, Kitasato University School of Medicine or the Department of Biological Sciences, Graduate School of Science, University of Tokyo. DNA extraction followed protocols established in the previous studies (*6*, *73*–*75*). Petrous bones were cut using an ultraviolet-irradiated disc and drill to access the inner part. Bone pieces were treated with 2 ml of lysis buffer containing 20 mM tris-HCl (pH 7.5), 0.7% *N*-lauroylsarcosine, 0.5 M EDTA (pH 8), and recombinant Proteinase K (0.65 U/ml), shaken at 900 rpm and 50°C in a ThermoMixer (Eppendorf) for 15 min. After discarding the supernatant, 2 ml of fresh lysis buffer was added, and the tubes were incubated for >16 hours with shaking at 900 rpm and 50°C in a ThermoMixer. Subsequently, the samples were centrifuged at 13,000*g* for 10 min, and the supernatants were transferred to ultrafiltration tubes (Amicon Ultra-4 Centrifugal Filter Unit 10K, Merck), diluted with 2 ml of Tris-EDTA (TE) buffer (pH 8.0) and centrifuged at 2300*g* until final volumes of ∼100 μl were obtained. These concentrates were then transferred to silica columns (MinElute PCR Purification Kit, QIAGEN) and purified according to the manufacturer’s instructions, except that DNA was eluted with EBT [Buffer EB (QIAGEN) supplemented with 0.005% Tween 20]. The EBT buffer was preheated to 60°C, and 60 μl was added. An extraction blank was incubated at this step to check for cross-contamination and background contamination and processed in parallel with the sample. DNA extracts were quantified by Qubit 4.0 (Thermo Fisher Scientific) and assessed with a Bioanalyzer (Agilent).

### Library construction and sequencing

Library construction was carried out following the protocol based on a previous study (*6*). We prepared double-stranded libraries with the NEBNext Ultra II DNA library preparation kit [New England Biolabs (NEB)] following the manufacturer’s instructions but with minor modifications. Specifically, we diluted the NEBNext Adaptor for Illumina (15 mM) to 1.5 mM with a 10-fold dilution and applied double-sided size selection with 90 μl of Agencourt AMPure XP solution (Beckman Coulter) for both removal of long fragments and recovery of short fragments. A blank was processed in parallel with each sample throughout library preparation. To check the percentage of human endogenous DNA and deamination levels, the initial sequencing using MiSeq (Illumina) was conducted at Kitasato University or the University of Tokyo.

For Ikawazu and Kosaku Jomon individuals, uracil-DNA-glycosylase (UDG)–treated DNA libraries were prepared (*76*). Approximately 5 ng of DNA extract was added to a reaction with a total volume of 50 μl, consisting of 5 μl of 10× buffer Tango (Thermo Fisher Scientific), 0.2 μl of 2.5 mM deoxynucleoside triphosphate (dNTP) mix (Thermo Fisher Scientific), 0.5 μl of 100 mM adenosine 5′-triphosphate (Thermo Fisher Scientific), 2.5 μl of T4 polynucleotide kinase (10 U/μl; Thermo Fisher Scientific), 3 μl of USER enzyme (1000 U/μl; NEB), and ultrapure water (Thermo Fisher Scientific) up to 50 μl. The mixture was then incubated at 37°C for 3 hours. Subsequently, a blunt-ending step was performed by adding 1 μl of T4 polymerase (Thermo Fisher Scientific) to the reaction mix, followed by incubation at 25°C for 15 min and then at 12°C for 5 min. The resulting product was purified using the MinElute kit (QIAGEN) with double elution in 30 and 23 μl of EBT buffer. The libraries were prepared using the NEBNext Ultra II DNA library preparation kit.

For some Ikawazu Jomon individuals (IK2010-2, IK2010-3, and IK2013-2), libraries were enriched using an in-solution hybridization protocol targeting approximately 1.2 million nuclear SNPs (the 1240k SNP panel) (*77*). UDG-treated DNA libraries were reamplified with NEBNext Q5 Master Mix (NEB) and P5 and P7 primers to a concentration of ∼30 ng/μl. In-solution hybridization was performed using myBaits Human Affinities Complete kit (Daicel Arbor Biosciences) with a hybridization temperature set to 60°C and incubated for 20 hours per round.

Libraries without enrichment, referred to as “shotgun,” were sequenced on a NovaSeq 6000 (Illumina) at the National Institute of Genetics in Japan, using 2 × 100–base pair (bp) paired-end sequencing on a NovaSeq 6000 at Macrogen in Japan with 2 × 150–bp paired-end, or a HiSeq 2500 (Illumina) at Genewiz in Japan with 2 × 100–bp paired-end. Enriched libraries, designated as “1240k capture” libraries, were sequenced on a MiSeq at the University of Tokyo, using 2 × 75–bp paired-end sequencing.

### Genetic sex determination

The genetic sex of our samples was determined following the methodology outlined by Skoglund *et al.* (*78*). Briefly, we computed the ratio of Y chromosome reads to the total number of sex chromosomes (Ry) and classified individuals as female if Ry < 0.016 or male if Ry > 0.075 (refer to table S1 for details).

### Ancient DNA authentication

The authenticity of the samples was assessed through several measures. First, we examined typical patterns of deamination toward read ends and estimated contamination rates on mitochondrial DNA (mtDNA) and Y chromosome in males. Deamination patterns were assessed with mapDamage 2 version 2.2.0 (fig. S1) (*79*).

The NEBNext Ultra DNA Library Prep Kit uses adaptors containing a uracil base, which is cleaved by the USER enzyme before library amplification. However, ancient DNA also harbors uracil bases at the 5′ end after the end repair. Consequently, the USER enzyme removes any uracil residues at the 5′ end, resulting in the absence of deamination damage in that region (*80*).

To evaluate mtDNA contamination, sequence reads were aligned to the revised Cambridge Reference Sequence mitochondrial genome (*81*), and the mapped data were processed using the same pipeline as described in the following section. The mtDNA contamination rate was assessed by calculating heterozygosity on the mtDNA using MitoSuite v1.1.0 (*82*). For determining nuclear genome contamination rates in males, heterozygosity was calculated on the X chromosome using ANGSD v0.939 (*83*).

### Sequence data processing

In addition to the 25 Jomon individuals sequenced in this study, the FASTQ files of previously published Jomon individuals (*n* = 17) (*6*, *7*, *84*–*86*) were downloaded from the DNA Data Bank of Japan (DDBJ) and the European Nucleotide Archive. Before mapping, AdaptorRemoval v2.3.1 (*87*) was used to trim ambiguous bases at the termini (--trimns), low-quality bases at the termini (--trimqualities), and short reads (--minlength 35), as well as to combine paired-end reads into a consensus sequence (--collapse). The processed reads were then mapped to the human reference genome (hg38) using the BWA-MEM algorithm (https://github.com/lh3/bwa). Subsequently, the BAM files generated by BWA-MEM were refined with Picard (https://broadinstitute.github.io/picard/) CleanSam, and duplicate reads were removed using Picard MarkDuplicates. The ends of the mapped reads were trimmed by TrimBam v1.0.15 in BamUtil (*88*): For non–UDG-treated samples, 2 bp from the 3′ ends and 10 bp from the 5′ ends were hard-clipped; for UDG-treated samples, 2 bp from both the 5′ and 3′ ends of mapped reads were trimmed. After merging BAM files from the same individual into one BAM file, mapped reads with a mapping quality below a Phred score of 30 were filtered out using SAMtools v1.6 (*89*).

### Genotype imputation

We conducted genotype imputation and haplotype phasing for the Jomon genomes including low-coverage individuals using GLIMPSE2 (*90*). We used phased VCF files from 9290 modern Japanese individuals, reported by Kawai *et al.* (*91*), as the reference panel, along with 2482 individuals from the 1KG including 104 Japanese individuals (*92*). We followed the tutorial on the GLIMPSE2 website. First, the reference panel was divided into chunks using the GLIMPSE2_chunk command and converted into GLIMPSE2’s binary file format with GLIMPSE2_split_reference. We then ran the GLIMPSE2_phase command for each chunk to perform genotype imputation and phasing, and the imputed chunks of Jomon individuals were ligated using the GLIMPSE2_ligate command. To check the accuracy of the genotype imputation in low-coverage Jomon individual genomes, we downsampled the high-coverage data of Jomon individuals (IK2010-1 and FUN23) to low coverage (1×) using SAMtools and compared the original and imputed genotypes with GLIMPSE2_concordance. The imputed genomes of 42 Jomon individuals were combined with the VCF files of 2482 individuals from the 1KG using the bcftools merge command for subsequent analyses. The 42 Jomon individuals were categorized by their regional origins within the Japanese archipelago, based on their sampling sites, as follows: 2 from Hokkaido, 27 from Kanto, 6 from Tokai, 4 from Hokuriku, and 3 from Chugoku-Shikoku (Fig. 1B and tables S1 to S3).

### Principal components analysis and ADMIXTURE analysis

We carried out principal components analysis (PCA) and ADMIXTURE analysis on the 42 imputed Jomon genomes and 503 EAS genomes from the 1KG, which include 104 JPT, 103 CHB, 104 CHS, 93 CDX, and 99 KHV genomes. We selected 1,310,568 biallelic SNPs with a minor allele frequency (MAF) exceeding 35%. The PCA was executed using PLINK v1.9 (*93*), while ADMIXTURE analysis was performed with ADMIXTURE version 1.3.0 (*94*), exploring different values of *K* (ranging from *K* = 2 to *K* = 5). Cross-validation analysis was conducted, and the configuration of *K* = 3, which exhibited the lowest cross-validation error, was chosen.

### TreeMix analysis

Phylogenetic analysis was performed using TreeMix (*95*) to elucidate the phylogenetic relationships among regional Jomon populations. We analyzed 41 of the 42 Jomon individuals, excluding JpKa6904 from Chugoku-Shikoku (see Fig. 1B) (*86*) due to its considerable antiquity compared to the other Jomon specimens, which might distort accurate regional phylogenetic relationships of the Jomon. For comparative analysis, we incorporated 503 genomes from EAS, along with 107 Yoruba (YRI) and 98 Utah residents (CEPH) with Northern and Western European ancestry (CEU) from the 1KG, and 15 Papuans from the Simons Genome Diversity Project (SGDP) (*96*), 10 Andamanese individuals (four Jarwa and six Onge) (*97*). From the dataset, we extracted 1,973,193 biallelic SNPs with a MAF exceeding 30%. In the analysis, considering the formation of modern Japanese through the admixture of the Jomon lineage and continental EASs, we assumed a single admixture pulse from the Jomon lineage to the continental EAS lineage.

### Estimation of genome-wide genealogy and population size change

To ascertain the population history of the Jomon people (i.e., the effective population size change and the divergence time from other Asians), we constructed genome-wide genealogies of the Jomon lineage and contemporary East and Southeast Asians by Relate software (*15*). We used 40 Jomon individuals with available radiocarbon dates and 503 continental EAS individuals from the 1KG and 15 Papuans from SGDP and 10 Andamanese (four Jarwa and six Onge) from Mondal *et al.* (*97*).

Input files for Relate were prepared using RelateFileFormats and PrepareInputFiles.sh. Ancestral sequences of *H. sapiens* (GRCh38) from Ensembl release 103 were used for the ancestral sequence analysis, and the StrictMask from the 1KG was used for the genomic masking.

We estimated the genome-wide genealogy with the “Relate” command, setting the mutation rate at 1.25 × 10^−8^ per base per generation (-m 1.25e-8) and an effective population size to 30,000 (-N 30,000). Sample ages (generations before present) of each Jomon individual were set by --sample_ages option, assuming a human generation time of 28 years. We then estimated the population size change and divergence times between the Jomon lineage and the other East and Southeast Asians with the EstimatePopulationSize.sh script, using the estimated genome-wide genealogies of the Jomon lineage and modern East and Southeast Asians (.anc, .mut) as input files.

### Genetic influence from northern UP populations

To further examine the possibility of gene flow from northern UP populations into the Jomon lineage, we calculated *f*_4_ statistics implemented in ADMIXTOOLS 2 (*98*). Specifically, we tested for asymmetric allele sharing between the Jomon and a northern UP population [Yana1, ∼31,000 B.P. (*29*)], relative to the Andamanese from Mondal *et al.* (*97*), a proxy for southern-route populations closely related to Hòabìnhian hunter-gatherers (*7*), and vice versa. Two sets of *f*_4_ statistics were computed. The first, *f*_4_(YRI, Yana; X, Andamanese), evaluates whether population X shares alleles with Yana, indicating potential gene flow from northern-route ancestry. The second, *f*_4_(YRI, Andamanese; X, Yana), evaluates whether population X shares alleles with the Andamanese, reflecting gene flow from southern-route ancestry.

For these analyses, we used publicly available VCF files. The dataset included modern genomes from the 1KG (*92*), SGDP (*96*), Human Genome Diversity Project (HGDP) (*99*), and the Korean Personal Genome Project (KPGP) (*100*). Ancient genomes included Jomon individuals from multiple regions across the Japanese archipelago, as well as Kolyma1 (*29*), a 14× coverage genome from an individual dated to ∼9800 years ago, excavated from Duvanny Yar in northeastern Siberia. Kolyma1 served as a representative of ancient Siberian ancestry.

We retained a total of 897,958 SNPs with a MAF of >0.35 in the 1KG dataset to ensure high-confidence allele calls. Population X included regional Jomon populations and a range of modern populations, grouped into regional categories based on superpopulation classifications for each dataset: EASs/Southeast Asians, Central Asians/Siberians, and Native Americans.

### Archaic introgression

We conducted SPrime analysis (*31*) to identify archaic segments introgressed from Neanderthal or Denisovan into the genomes of Jomon. SPrime is a reference-free tool designed to detect these segments by leveraging the strong LD present among archaic variants within the genomes of modern populations. We used SPrime v07Dec18.5e2, with YRI from the 1KG serving as a nonadmixed modern population. In addition to the Jomon, we also identified archaic segments in EASs from 1KG JPT, CHB, CHS, CDX, KHV, Papuan from the SGDP, and Andamanese (Onge and Jarwa) from Mondal *et al.* (*97*). We adopted the default settings for all SPrime optional parameters, thereby classifying genetic segments with an SPrime score exceeding 100,000 as “archaic segments.” To calculate the match rate to Denisovan (*101*) and Altai Neanderthal (*102*), we followed the same method described in the original SPrime paper, i.e., the number of matches divided by the number of compared sites for each genetic segment identified as archaic by SPrime. We considered archaic segments with a match rate to Altai Neanderthal of <0.2 and a match rate to Altai Denisovan of >0.2 to be introgressed segments from Denisovan lineages. We downloaded the whole-genome VCFs of Altai Neanderthal and Denisovan that were mapped to the hg19 reference sequence from http://cdna.eva.mpg.de/neandertal/altai/. To calculate the match rate, the chromosomal positions of the detected Jomon and EAS archaic segments were converted from hg38 to hg19 using liftOver (*103*). To compare the distribution of Denisovan match rates across populations, we calculated the 1-Wasserstein distance (also known as Earth mover’s distance) between Jomon and each of the other populations. This metric quantifies the dissimilarity between two probability distributions based on their cumulative differences. For each pairwise comparison, we assessed statistical significance using a permutation test with 10,000 replicates, in which group labels were randomly shuffled and the Wasserstein distance recalculated to generate a null distribution. The empirical *P* value was then defined as the proportion of permuted distances equal to or greater than the observed distance. To control for potential bias due to differences in the number of archaic segments detected in each population, we randomly downsampled each comparison population to match the number of Denisovan-like segments identified in the Jomon. This ensured that differences in distribution were not driven by imbalances in sample size.

To complement the reference-free detection of Denisovan segments performed with SPrime, we additionally applied IBDmix (*37*), a source-aware method that identifies archaic haplotypes based on excess allele sharing with a high-coverage Denisovan genome. IBDmix was run on imputed and phased Jomon genomes using default parameters, and we retained segments of ≥50 kb with a log-odds (LOD) score of >5. The same procedure was applied to 1000 Genomes CHB, CHS, KHV, and CDX populations (excluding JPT), as well as Papuans for calibration. Population-specific segments were defined as those not overlapping segments identified in any comparison populations. This supplementary analysis was used to confirm whether SPrime-based conclusions—specifically, the absence of a Jomon-specific Denisovan introgression pulse—were supported by an independent method.

### Genome-wide selection scan

To investigate the genetic adaptations of the Jomon lineage after their early divergence from the continental EASs, we detected positive natural selection signals. Given our interest in discerning differences in genetic adaptation between the Jomon and continental EASs, we used the XP-nSL method to detect regions with particularly long-range haplotypes (i.e., selective sweep signals) in the Jomon.

XP-nSLs were calculated for 12,603,233 SNPs by Selscan software (*50*). A positive XP-nSL value exceeding the predefined empirical significance threshold suggests signals of positive natural selection in the Jomon lineage, whereas a negative value exceeding the corresponding threshold in the opposite direction indicates positive natural selection in the Han Chinese.

To identify phenotypes that spread among the Jomon through positive natural selection, we devised the rXP-nSL index by combining XP-nSL with summary statistics (β and *P* values) obtained from previous GWAS on 62 traits in modern Japanese (*51*, *52*, *104*, *105*). Given that the GWAS data are based on the hg19 sequence, we converted chromosomal positions from hg19 to hg38 using liftOver.

The rXP-nSL index is designed to detect trait-associated genetic regions enriched with SNPs exhibiting high XP-nSL values. In this study, we used LD-clumping to define independent trait-associated genomic regions. Although GWAS aims to identify variants affecting traits or diseases, association signals often extend to neighboring SNPs through LD. Rather than selecting a single representative SNP from each association peak, we used LD-clumping to group GWAS-associated SNPs that are in LD and define trait-associated genetic regions. Specifically, trait-associated regions were defined as clusters of GWAS-associated SNPs connected through LD within a 1-Mb window.

We combined GWAS data of modern Japanese individuals (effect allele, effect size β, and *P* value for each SNP) from previous studies with VCF files of the modern Japanese (JPT) from the 1KG. Using PLINK, we executed LD-clumping with the options --clump-p1 5e-8, --clump-kb 500 --clump-r2 0 for 1KG JPT modern Japanese genomes. We adopted --clump-kb 500 to set the window length at 1 Mb. With --clump-p1 5e-8, we set the GWAS *P* value threshold at 5.0 × 10^−8^, and with --clump-r2 0, we set the LD coefficient *r*^2^ to be greater than 0, considering trait-associated SNP pairs that are within 1-Mb window as being in the “same” trait-associated region. Subsequently, we calculated the mean value of XP-nSL across the *n* trait–associated SNPs within the trait-associated region of the Jomon (fig. S11). The rXP-nSL was then calculated by the following procedure: (i) for each target region, randomly sample a set of *n* SNPs from the whole genome such that each sampled SNP matches the corresponding target SNP in Jomon allele frequency, recombination rate, LD score, and local SNP density; (ii) obtain the mean XP-nSL of the sampled *n* SNPs; (iii) repeat steps (i) and (ii) 100,000 times to obtain the null distribution of the mean XP-nSL for regions containing *n* matched SNPs; (iv) based on the mean and SD of this null distribution, obtain rXP-nSL by the following formula

$$\text{rXP-nSL}=\frac{\text{mean}\;{\text{XP-nSL}}_{\text{target}}-\text{mean}\;{\text{XP-nSL}}_{\text{null}}}{\text{SD}\left({\text{XP-nSL}}_{\text{null}}\right)}$$

Trait-associated genetic regions with rXP-nSL greater than 11.25, corresponding to the top 5% of the 1702 trait-associated regions identified across 62 GWASs, were interpreted as having undergone positive natural selection in the Jomon lineage (fig. S12). For each trait-associated genetic region, we evaluated whether the haplotype under positive selection in the Jomon lineage was associated with an increase or decrease in the focal trait by comparing the mean 2β*f* within each trait-associated genetic region, the mean value of the product of 2 times the effect allele frequency of each SNP and the effect size (β) in the focal trait–associated genetic region between the Jomon and the Han Chinese. If the mean of 2β*f* was greater for the Jomon than for the Han Chinese, we considered it as having a “relatively positive effect” on the Jomon. Conversely, if the mean of 2β*f* was smaller for the Jomon than for the Han Chinese, then we regarded it as having a “relatively negative effect” on the Jomon.

To evaluate the robustness of the rXP-nSL–based genome-wide scan, we additionally conducted sensitivity analyses for (i) LD-clumping parameters and (ii) alternative EAS GWAS effect-size panels. First, we repeated the rXP-nSL analysis across 12 combinations of LD thresholds (*r*^2^ = 0, 0.05, 0.1, and 0.2) and window sizes (--clump-kb = 250, 500, and 1000). Second, to assess the extent to which our findings depend on the choice of effect-size panel, we repeated the analysis using summary statistics from an independent large-scale EAS GWAS of Taiwanese individuals (*53*), which analyzed 92,615 participants across 32 traits, including BMI and TGs.

### Comparison between the Jomon and Greenlandic Inuit for frequency of alleles associated with diet and cold adaptation

Previous studies on the population genomics of Greenlandic Inuit (GI) have detected signals of positive natural selection at various loci, such as *FADS1*, *FADS2*, and *FADS3* on chromosome 11, *TBX15* and *WARS2* on chromosome 1, and *FN3KRP* on chromosome 17 (*56*). These loci are thought to be associated with adaptations to the cold climates of their settlement areas and their diets rich in omega-3 PUFAs, which are prevalent in seafood (*56*).

Considering the potential similarity in cold environment adaptations between the Jomon and GI, it is plausible that the allele frequencies in the Jomon exhibiting positive natural selection signals observed in GI would more closely resemble those of GI than populations sharing a more recent common ancestor with the Jomon, such as Han Chinese. To explore this hypothesis, we calculated the *f*_4_(CEU, GI; Jomon, CHB) (*106*) statistic for genome-wide SNPs.

Negative *f*_4_ values indicate that allele frequencies in the Jomon are closer to those in GI than to those in Han Chinese. We obtained the allele frequencies in GI for 94,577 genome-wide SNPs from the previous study (*56*) and performed the *f*_4_(CEU, GI; Jomon, CHB) calculation using a custom script in R.

### Haplotype network construction

To investigate the haplotype structures surrounding cold-adaptive variants, we focused on five loci: *APOA5* (rs662799), *FTO* (rs11642015), *UCP1* (rs3113195), *FADS2* (rs74771917), and *TRPM8* (rs10166942). For each locus, we extracted SNPs located within ±500 kb and in LD with the focal SNP (*r*^2^ > 0.1), using phased genotype data. The haplotype network was constructed and visualized using the software PopART (*107*), using the median-joining network algorithm. This analysis included sequences from Jomon individuals and EAS from the 1KG, Andamanese, and Papuan individuals. The resulting networks enabled us to assess the evolutionary relationships among haplotypes and to determine whether the derived alleles associated with cold adaptation were shared across populations or specific to the Jomon lineage.

### Estimation of allele frequency trajectory

To elucidate the timing of positive natural selection within the population history of the Jomon lineage, we conducted CLUES2 (*108*, *109*) analysis to estimate the allele frequency trajectories and selection coefficients of a trait-associated SNP that showed a strong selective sweep signal in the Jomon lineage. CLUES2 can be run on ancient genomes and makes use of ancient genotype probabilities, sample ages, the Relate output genealogies (.anc and .mut), and the population size change (.coal). As the primary target SNP, we analyzed rs662799 (A/G) in *APOA5*, the variant showing the strongest TG association in GWAS (*P* < 1 × 10^−320^). To assess robustness, we additionally applied CLUES2 to 18 SNPs within the *APOA5* region that exhibited extremely strong associations with TG levels (GWAS *P* < 1 × 10^−200^).

Relate genealogies and population-size histories were inferred using Relate v1.2.1 on all 40 radiocarbon-dated Jomon individuals, generating matched .anc, .mut, and .coal files under a consistent time origin. Because our dataset consists solely of ancient individuals, the most recent Jomon sample (IK002) dates to ∼96 generations ago, and, therefore, the allele frequency trajectories inferred by CLUES2 begin at that point. For each SNP, allele frequency trajectories were inferred following the standard CLUES2 workflow, using branch lengths sampled from Relate genealogies and incorporating ancient genotype probabilities, sample ages, and the corresponding Relate-generated population-size histories.

Selection coefficients (*s*) were estimated using the --timeBins option, dividing time into periods corresponding to the Last Glacial period and the post–Last Glacial period, with a cutoff at 10,000 years ago. This framework enables comparison of selection strength between climatic periods while maintaining complete consistency among genealogies, sample ages, and population-size histories.

## Supplementary Materials

### The PDF file includes:

NCBN Controls WGS Consortium members Supplementary Text Figs. S1 to S17 Legends for tables S1 to S5, S7, S8, and S10 to S12 Tables S6 and S9 References
