# Supplementary material for: Jomon genomics reveal cold adaptation in Upper Paleolithic hunter-gatherers of eastern Eurasia
Source: Sci Adv. 2026 Aug 12;12(33):eaea7469. doi: 10.1126/sciadv.aea7469 (PMC13464473; doi:10.1126/sciadv.aea7469)
Supplement: Supplementary file 1 [file sciadv.aea7469_sm.pdf]

Science Advances
AAAS

Supplementary Materials for

Jomon genomics reveal cold adaptation in Upper Paleolithic hunter-gatherers of eastern Eurasia

Yusuke Watanabe *et al.*

Corresponding author: Hiroki Oota, hi2hiroki@g.ecc.u-tokyo.ac.jp

Sci. Adv. **12**, eaea7469 (2026)
DOI: 10.1126/sciadv.aea7469

The PDF file includes:

NCBN Controls WGS Consortium members
Supplementary Text
Figs. S1 to S17
Legends for tables S1 to S5, S7, S8, and S10 to S12
Tables S6 and S9
References

Other Supplementary Material for this manuscript includes the following:

Tables S1 to S5, S7, S8, and S10 to S12

NCBN Controls WGS Consortium members

Hatsue Ishibashi-Ueda,[1] Tsutomu Tomita,[1] Michio Noguchi,[1] Ayako Takahashi,[1]
Yu-ichi Goto,[2] Sumiko Yoshida,[3] Kotaro Hattori,[3] Ryo Matsumura,[3] Aritoshi Iida,[4]
Yutaka Maruoka,[5] Hiroyuki Gatanaga,[6] Akihiko Shimomura,[5] Masaya Sugiyama,[7] Satoshi Suzuki,[5], Kengo Miyo,[8]
Yoichi Matsubara,[9] Akihiro Umezawa,[10] Kenichiro Hata,[11] Tadashi Kaname,[12]
Kouichi Ozaki,[13] Haruhiko Tokuda,[13] Hiroshi Watanabe,[13] Shumpei Niida,[13]
Eisei Noiri,[14] Koji Kitajima,[14] Yosuke Omae,[14,15] Reiko Miyahara,[14] Hideyuki Shimanuki,[14]
Yosuke Kawai,[15] and Katsushi Tokunaga[14,15]

[1] NCVC Biobank, National Cerebral and Cardiovascular Center, Suita, Osaka 564-8565, Japan

[2] Medical Genome Center, National Center of Neurology and Psychiatry, Kodaira, Tokyo 187-8551, Japan

[3] Department of Bioresources, Medical Genome Center, National Center of Neurology and Psychiatry, Kodaira, Tokyo 187-8551, Japan

[4] Department of Clinical Genome Analysis, Medical Genome Center, National Center of Neurology and Psychiatry, Kodaira, Tokyo 187-8551, Japan

[5] NCGM Biobank, National Center for Global Health and Medicine, Shinjuku-ku, Tokyo 162-8655, Japan

[6] AIDS Clinical Center, National Center for Global Health and Medicine, Shinjuku-ku, Tokyo 162-8655, Japan

[7] Department of Viral Pathogenesis and Controls, Research Institute, National Center for Global Health and Medicine, Ichikawa, Chiba 272-8516, Japan

[8] Center for Medical Informatics and Intelligence, National Center for Global Health and Medicine, Shinjuku-ku, Tokyo 162-8655, Japan

[9] National Center for Child Health and Development, Setagaya-ku, Tokyo 157-8535, Japan

[10] Center for Regenerative Medicine, National Center for Child Health and Development, Setagaya-ku, Tokyo 157-8535, Japan

[11] Department of Maternal-Fetal Biology, National Center for Child Health and Development, Setagaya-ku, Tokyo 157-8535, Japan

[12] Department of Genome Medicine, National Center for Child Health and Development, Setagaya-ku, Tokyo 157-8535, Japan

[13] Research Institute, National Center for Geriatrics and Gerontology, Obu, Aichi 474-8511, Japan

[14] Central Biobank, National Center Biobank Network, Shinjuku-ku, Tokyo 162-8655, Japan

[15] Genome Medical Science Project (Toyama), Research Institute, National Center for Global Health and Medicine, Shinjuku-ku, Tokyo 162-8655, Japan

Supplementary Text

1. Genotype Imputation in Jomon Individuals and Ancestral Recombination Graph (ARG) Bias from Reference Panel Mismatch

We analyzed 42 Jomon genomes of newly sequenced (25 bones, see table S1 and 2) and previously reported (17, see table S3) samples (*6*, *7*, *84–86*), including 3 high-coverage samples (two of which were newly sequenced), 18 samples with coverage ranging from 1x to 10x, and 21 low-coverage samples (< 1x). This study includes Jomon samples collected from various regions across the Japanese archipelago (Fig. 1B). These ancient Jomon genomes were imputed and phased by GLIMPSE2 software (*90*), a tool designed for imputation of low-coverage genomic sequences. Given that approximately 20% of their genomic components of modern Hondo Japanese are reported to be derived from the Jomon (*7*, *14*, *84*), and considering the absence of available direct descendants from the Jomon in current populations today with the exception of Japanese archipelago populations, employing the modern Japanese as a reference was deemed necessary for the imputation of the Jomon genomes. For this purpose, we utilized a reference panel comprising the genomes of 9,290 modern Japanese individuals from the National Center Biobank Network (NCBN) (*91*), in addition to 2,482 individuals from the 1000 Genomes Project (1KG) (*92*) including 104 modern Japanese (JPT). We verified the imputation accuracy using high-coverage Jomon genomes [FUN23 (*84*) and IK2010-1] and found that the imputation accuracy, especially at polymorphic sites with minor allele frequency less than 1%, improved when employing a larger number of Japanese individuals in the reference panel (fig. S2A).

To illustrate the importance of using ancestry-matched reference panels for deeply diverged ancient genomes, we conducted a control analysis using the same set of 40 Jomon individuals, imputed with a non-ancestry-matched panel (same as the “1KG without modern Japanese reference panel” in fig. S2A). The resulting demographic trajectories inferred by Relate showed a marked shift in the estimated effective population size (Ne), particularly after the divergence from continental East Asians (fig. S2B). Specifically, the Ne curves for the Jomon individuals were pulled closer to those of present-day Han Chinese (CHB), leading to a distortion of population-specific demographic signals. This pattern suggests a systematic bias in genealogical inference: when an ancient genome is imputed using an inadequate reference, its reconstructed ancestry may be artificially drawn toward the genetic profile of the reference population. In other words, the estimated genealogy is "pulled" toward the structure of the imputation panel.

Using the imputed genotypes of the Jomon alongside contemporary East Asian genomes from the 1KG, we conducted principal component analysis (PCA) and ADMIXTURE analysis (*94*) (fig. S2C and 2D). These analyses showed the 25 Jomon individuals in this study cluster with the 17 Jomon individuals previously published, further suggesting the Jomon were geographically and genetically isolated from the modern East Asians in the continental region (*6*, *14*, *84*, *86*).

2. The correlation between our findings and the archaeological evidence from the Upper Paleolithic (UP) in the Japanese archipelago

The UP occupation began around 38,000 yr cal BP (*11*) and continued until about 15,000 yr cal BP on P-Honshu, extending from 15,000 to 12,000 yr cal BP on Hokkaido, south of PSHK (*16*, *17*). The UP period is divided into three sub-stages – early, middle, and late – characterized by lithic reduction patterns, toolkit compositions, stone-tool types, and date of the sites. Notably,

the middle UP stage, spanning approximately 30,000-20,000 yr cal BP, shows heightened mobility with a blade-based tool-kit assemblage (*12*, *16*, *18*, *19*, *110*).

During the middle UP, the blade-point industry flourished particularly for the northern P-Honshu, marked by prismatic and conical blade cores, and by relatively large blade-based tools including backed points, basally retouched points, burins, endscrapers and sidescrapers, exhibiting a composition akin to UP assemblages found elsewhere in northern latitudes of Asia. These tools were highly standardized with intensive retouch, and toolkit diversity became clearly higher than the small flake-based trapezoid industry during the early UP (*10*, *18*). This particular blade-based reduction strategy was facilitated by the availability of high-quality raw materials such as obsidian and siliceous shale. Tools and blades were mostly manufactured at a limited number of hub sites nearby high-quality raw material sources, and transported to distant logistic satellite sites, suggesting that more scheduled foraging activities were accomplished than in the previous early UP assemblages. This period witnessed increased repeated occupation at certain locations and reduced residential movements, augmented by short-term, task-specific extraction locations (*18*, *111*).

Following the initial appearance of modern humans at 38,000 yr cal BP, drastic changes in the chronological distribution of radiocarbon dates were observed by the Kernel Density Estimate model (*71*, *112*, *113*) around 30,000 yr cal BP, both on P-Honshu and PSHK during Middle UP (*20*). Given the changes observed in cultural evidence during the middle UP, coupled with the estimated divergence time between the Jomon and continental East Asians (Fig. 2 and fig. S8), it is possible that the reduced connectivity of the Japanese archipelago from the East Eurasian continent just before or during the LGM resulted in a divergence between the UP populations on the Eurasian continent and those on the Japanese archipelago, leading to cultural transitions. The ecological background underwent drastic alteration from the temperate Pan-mixed forest dominated landscape, characterized by *Palaeoloxodon-Shinomegaceroides* complex by 30,000 yr cal BP, to a cool-temperate coniferous forest with patchy grassland accompanied by Mammoth fauna in the PSHK during the LGM, showing different pattern of smaller regional biomes (Fig. 1A) (*18*, *114*, *115*). The colder environment of both P-Honshu and PSHK during LGM shared several common faunal and floral species with those of continental northeastern Asia, possibly supporting migration from the northern landscape.

The subsequent decline in the frequency of cultural occupation preceding the Jomon period (*20*) closely aligns with our finding (Fig. 2), indicating a rapid decline in population size until the beginning of the Jomon period. The period around 20,000 years ago, encompassing the LGM, likely presented challenges for the UP population in the Japanese archipelago, potentially leading to a substantial decline in population size. Following the massive increase in effective population size after the LGM (Fig. 2), it is likely that the UP population, survivors of the ice age, formed the Jomon culture in the warming Japanese archipelago, epitomized by its adoption of distinctive pottery (*21*). The divergence between the Hokkaido Jomon and the Hondo Jomon occurred between 8,800 and 10,000 years ago. Archaeologically, it has been suggested that the human population in P-Honshu migrated to PSHK around 13,000 years ago (*20*), a finding supported by Fig. 2B, fig. S3 and 9 (*17*). Moreover, the absence of recovery in the effective population size in the Hokkaido Jomon lineage is consistent with the line of cultural evidence (*10*, *18*, *22*, *23*), indicating a decrease in the intensity of cultural occupation in Hokkaido around 15,000 years ago.

3. *TreeMix* analyses

The 42 Jomon genomes, collected from various regions of the Japanese archipelago as depicted in fig. S3, facilitated our phylogenetic analysis aimed at uncovering the regional genetic diversity of the Jomon. According to Jeong et al. (2023) (*116*), JpKa6904, the earliest Jomon specimens sequenced to date (*86*), was reported to be an outgroup to the more recent Jomon individuals by f-statistic based approaches. Our phylogenetic analysis, excluding JpKa6904 individual, showed that the northwestern Jomon lineage diverged from the southwestern Jomon lineage, suggesting a gradient of genetic diversity that extends from the southwest to the northeast across the Jomon population. The 41 Jomon individuals examined in this study span a broad temporal range, from approximately 6,000 to 2,700 years ago in each geographic region in the Japanese archipelago. Consequently, our results likely reflect geographical diversity within the Jomon population rather than temporal variations.

Our phylogenetic analysis revealed that Jomon individuals on Hokkaido diverged from those in northeastern Hondo, suggesting a migration from Honshu to Hokkaido (fig. S3) after the UP rather than the direct lineage of the Hokkaido Jomon from the UP inhabitants of the area. Our results suggest that these earlier inhabitants were genetically replaced by Jomon migrants from Honshu. This result is supported by robust archaeological evidence argued for a long time indicating cultural discontinuity during the transition from the UP to the Jomon, marked by abrupt changes in the entire lithic reduction system and the introduction of new cultural traits such as production of pottery (21–24).

To evaluate the robustness of the placement of the Jomon within the basal East Asian clade, we conducted additional TreeMix analyses including three ancient basal East Asian individuals—Tianyuan (*117*), Hoabinhian (La368) (*7*), and Xingyi_EN (*118*). These ancient genomes were aligned to the human hg38 reference with BWA-MEM, and genotype calls were generated on the 1240K panel using bcftools. The resulting VCFs were then merged with 1000 Genomes EAS, YRI, CEU, and Jomon datasets before running TreeMix. Four runs were performed varying two conditions: (i) inclusion versus exclusion of the low-coverage Hoabinhian individual La368 (0.5×), and (ii) use of either all 1240K SNPs or transversions only (figs. S4-S7). While these parameter changes led to different branching orders among ancient basal East Asians, a consistent pattern emerged across all analyses: the Jomon always occupied the most derived position within the basal East Asian cluster. This qualitative stability supports that the inferred placement of the Jomon as a recently diverged basal East Asian lineage is robust to SNP ascertainment and sample composition.

4. Our findings and their implications for the initial peopling of the Americas

We have confirmed that the Hokkaido Jomon, represented by Funadomari individuals, diverged from a single lineage shared with the Hondo Jomon (Fig. 2 and fig. S3 and S9). All Jomon individuals examined so far thus have consistently indicated a shared common ancestor. These findings from our analysis may offer insights into the initial peopling of the Americas.

The discovery of bifacial stemmed points at the Cooper’s Ferry site (Idaho, USA), dating back to ~15,780 yr cal BP, indicates a closest similarity to Late UP stemmed point technology in PSHK. This suggests the existence of cultural connections with UP peoples at the Pacific Rim, potentially tracing back to some of the earliest peoples in the Americas between ~22,000 and 16,000 years ago (*119*, *120*). Other archaeological evidence also supports this hypothesis; for instance, bifacial stemmed point technology of this old did not emerge in any region of northeast Asia, including Beringia until around 14,200 yr cal BP (*121*). Despite the cultural evidence

supporting this scenario, further discussion must be postponed due to the absence of human skeletal remains and subsequent lack of ancient genomic data from PSHK.

Furthermore, the “Out-of-Japan” hypothesis is challenged by various lines of evidence from bioanthropology. Morphological analysis of skeletal remains, along with data on haploid markers (both on mtDNA and Y-chromosomes), and whole genomic analyses of Holocene Jomon, all suggested that the Jomon are unlikely to be direct ancestors of the ancestral and modern Native Americans (*30*).

However, our analysis indicates that Hokkaido Jomon diverged from those of Hondo Jomon around 10,000-8,000 years ago (Fig. 2B and fig. S9). The UP peoples who inhabited PSHK before the migration of Jomon people to the isolated island of Hokkaido in Holocene likely belong to a different lineage, diverging from ancestral East Eurasians. As previously suggested, UP in PSHK, dated between ~30,000 and 12,000 yr cal BP, exhibited a higher diversity of lithic assemblage, including various stone tool technologies and lithic reduction sequences such as small flake-based, blade-based, flake-based, bifacial, and microblade (*10*, *16*, *122*). This higher diversity in lithic assemblage in PSHK may suggest a more complex prehistory of human migrations in and out of the region, situated in the ecotone between the cool-temperate coniferous forest to the south and patches of sub-arctic open forest and steppe to the north (*20*, *23*). These pre-Jomon people in PSHK still stand as candidates for the first Americans. Further testing of the “Out-of-Japan” hypothesis or coastal migration theory is challenging based solely on the Jomon genomes presented in this study. Detailed analysis of the UP peoples in PSHK is imperative.

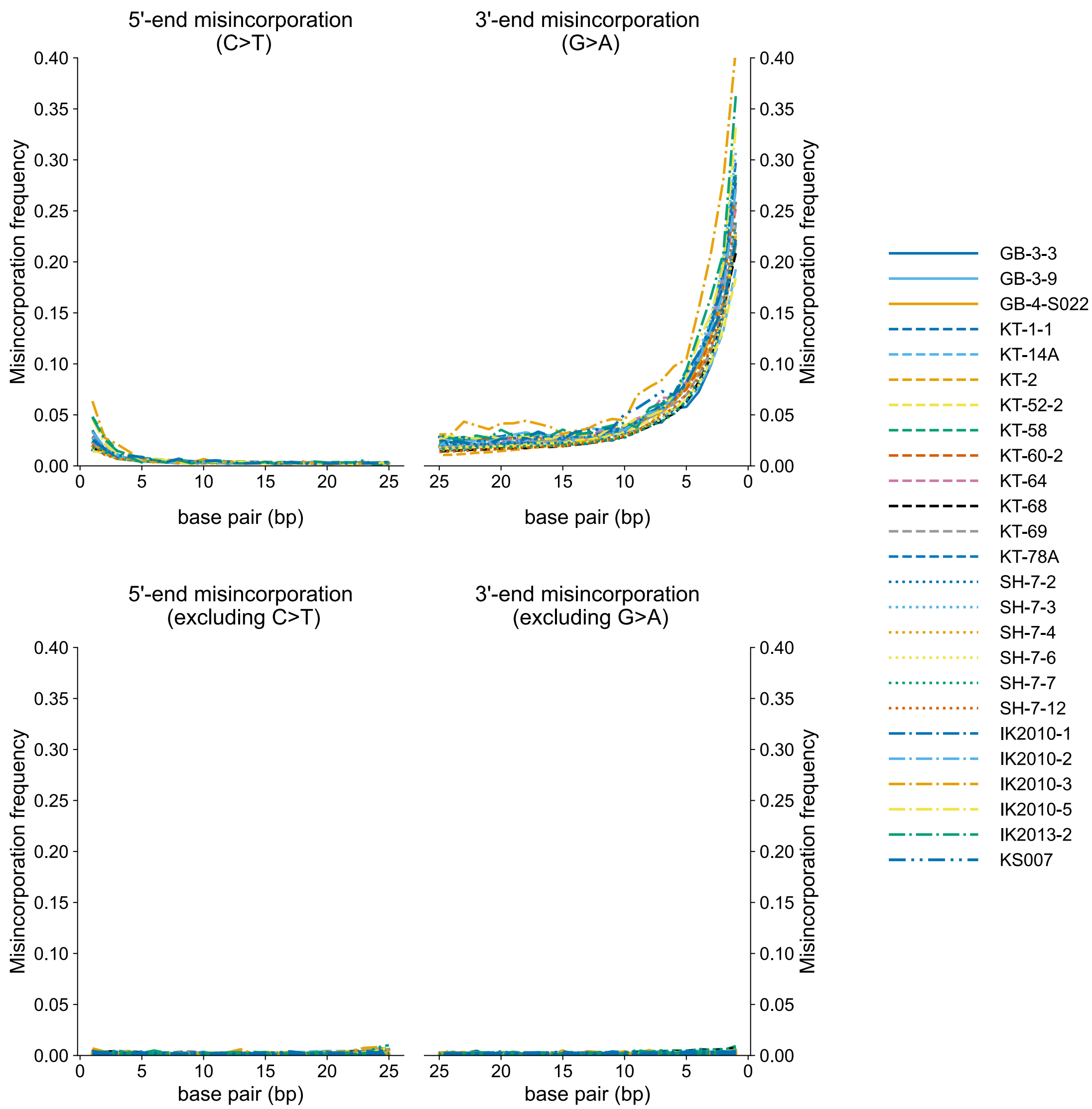

Fig. S1. DNA damage plots for newly sequenced Jomon individuals. The upper left plot shows C>T misincorporations at the 5’ end, and the upper right plot shows G>A misincorporations at the 3’ end (characteristic deamination patterns of ancient DNA). The lower left plot displays misincorporations other than C>T at the 5’ end, while the lower right plot displays misincorporations other than G>A at the 3’ end.

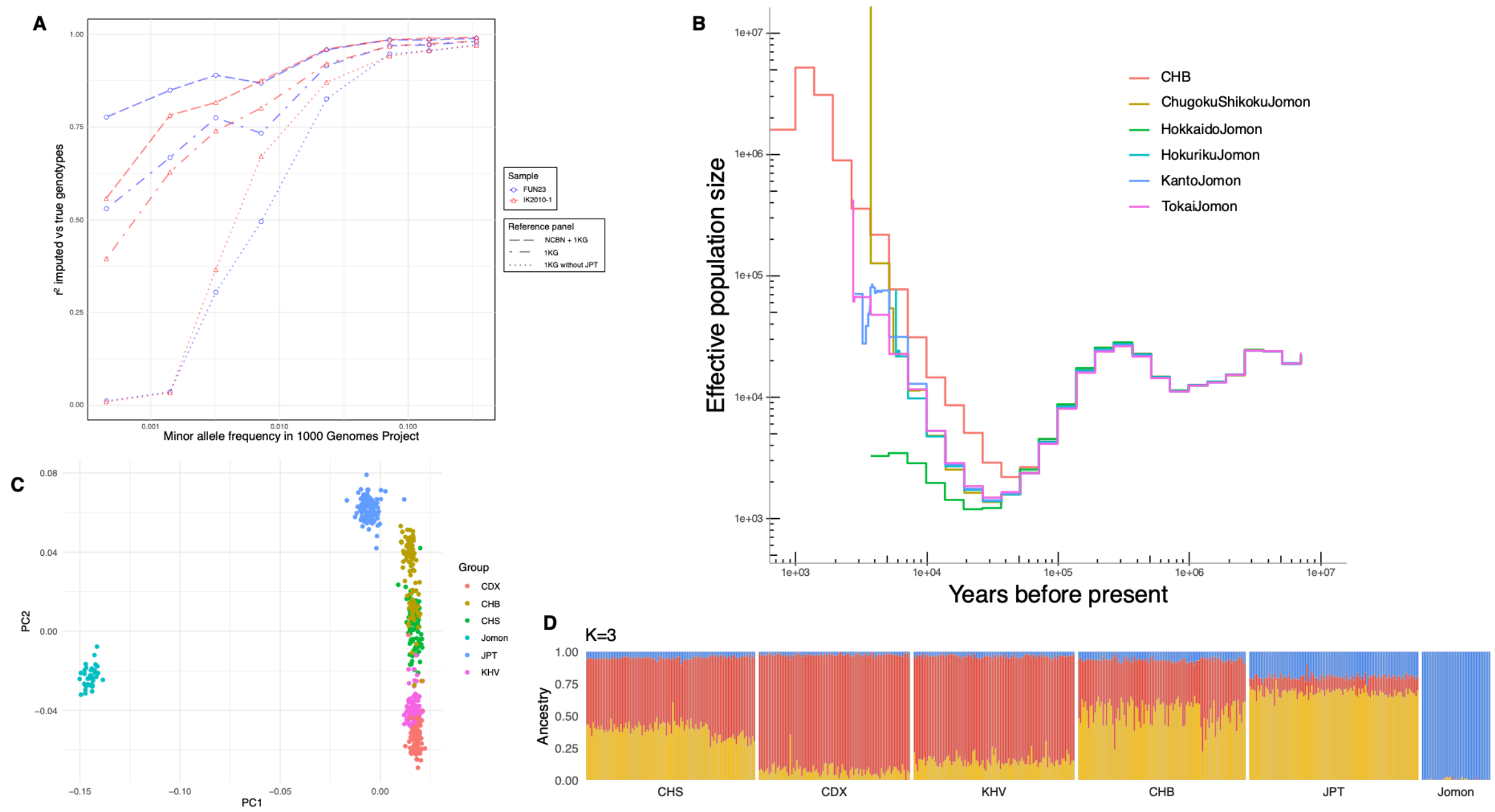

Fig. S2. Imputation performance, principal component analysis (PCA) and ADMIXTURE analysis of the Jomon individuals. (A) Imputation accuracy (r^2, y axis) on chromosome 22 for two high-coverage Jomon individuals (FUN23 (*14*) and IK2010-1) across the NCBN + 1KG, 1KG, 1KG without modern Japanese reference panels. Accuracy is plotted against log-scaled minor allele frequency in the 1KG panel (x axis). (B) Effective population size change within the Jomon lineage imputed with a non-ancestry-matched panel (same as the "1KG without modern Japanese reference panel" in fig. S2A) (C) PCA plot for 42 Jomon imputed genomes and 1KG East Eurasian genomes (modern Japanese, JPT; Han Chinese, CHB, Southern Han Chinese, CHS; Dai Chinese, CDX; Kinh Vietnamese, KHV) (D) ADMIXTURE analysis for 42 Jomon imputed genomes and 1KG East Eurasian genomes (K = 3)

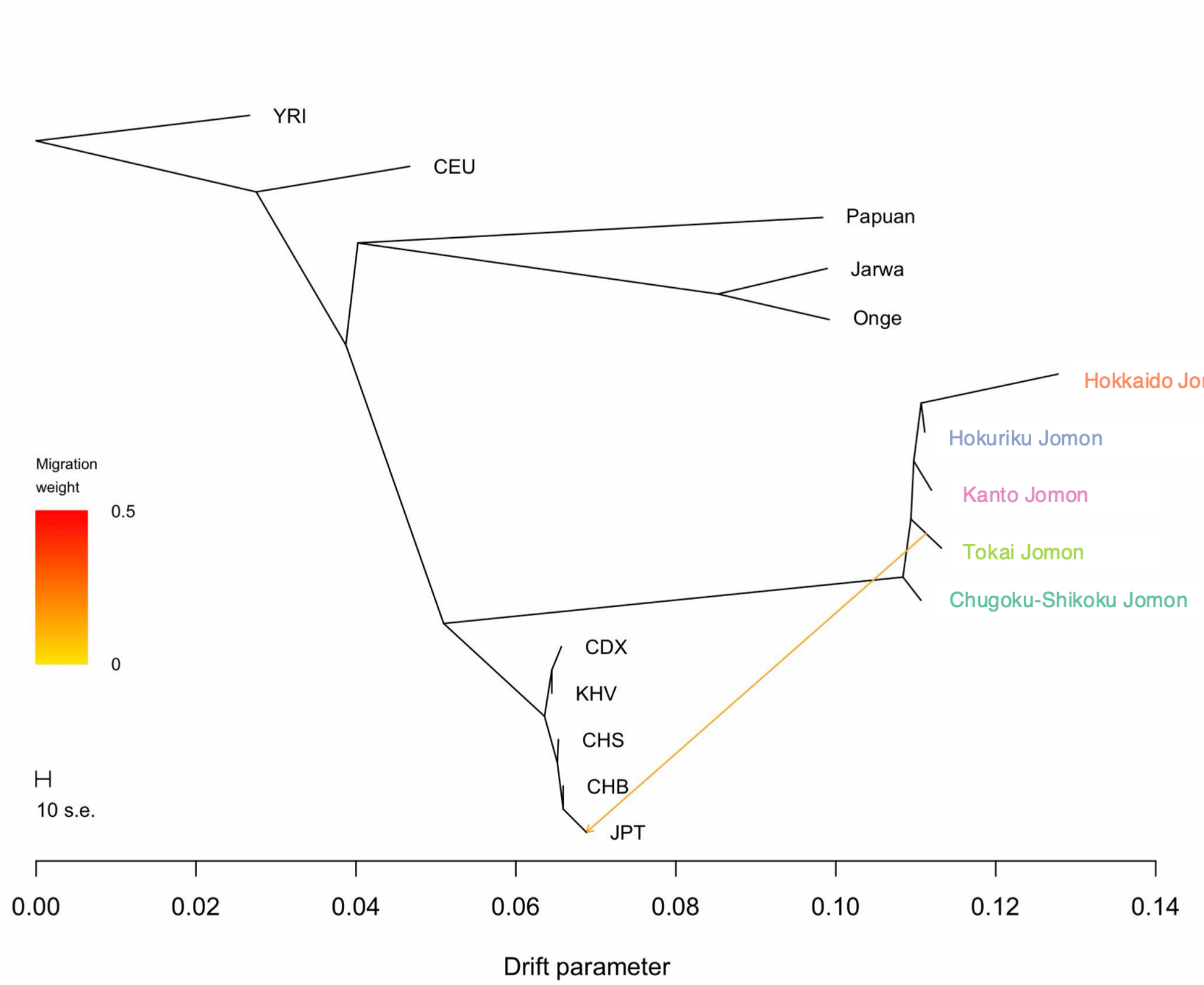
YRI
CEU
Papuan
Jarwa
Onge
Hokkaido Jomon
Hokuriku Jomon
Kanto Jomon
Tokai Jomon
Chugoku-Shikoku Jomon
CDX
KHV
CHS
CHB
JPT
Migration weight
0.5
0
10 s.e.
0.00
0.02
0.04
0.06
0.08
0.10
0.12
0.14
Drift parameter

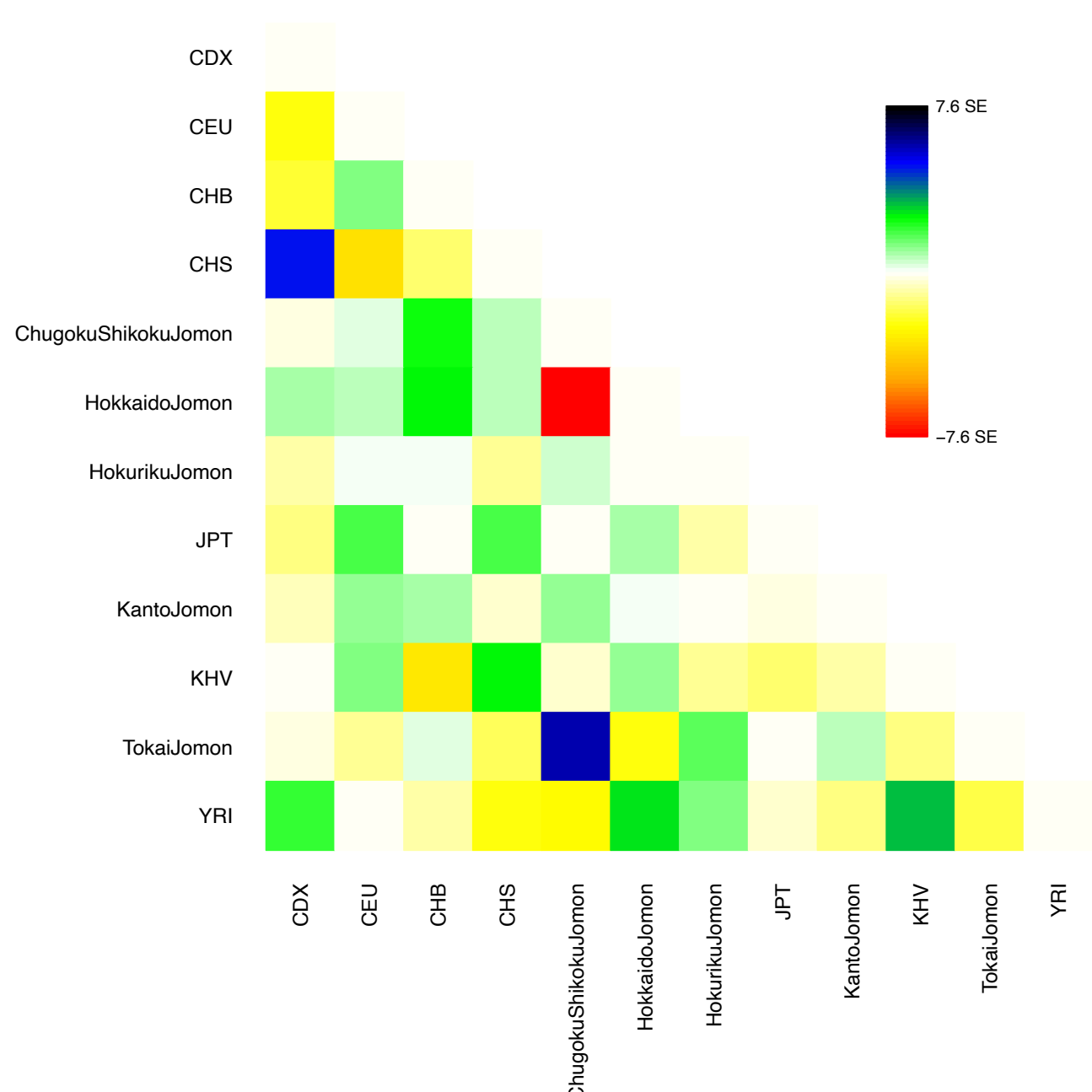
CDX
CEU
CHB
CHS
ChugokuShikokuJomon
HokkaidoJomon
HokurikuJomon
JPT
KantoJomon
KHV
TokaiJomon
YRI
7.6 SE
−7.6 SE

Fig. S3. Phylogenetic analysis of the Jomon. Maximum likelihood phylogenetic tree of regional populations of the Jomon reconstructed by *TreeMix* under a model of one migration. The upper panel shows the inferred tree; the lower panel shows the corresponding residual fit plot. Abbreviations: Japanese in Tokyo, Japan, JPT; Han Chinese in Beijing, China, CHB; Han Chinese South, CHS; Chinese Dai in Xishuangbanna, China, CDX; Kinh in Ho Chi Minh City, Vietnam, KHV; Utah residents (CEPH) with Northern and Western European ancestry, CEU; Yoruba in Ibadan, Nigeria, YRI.

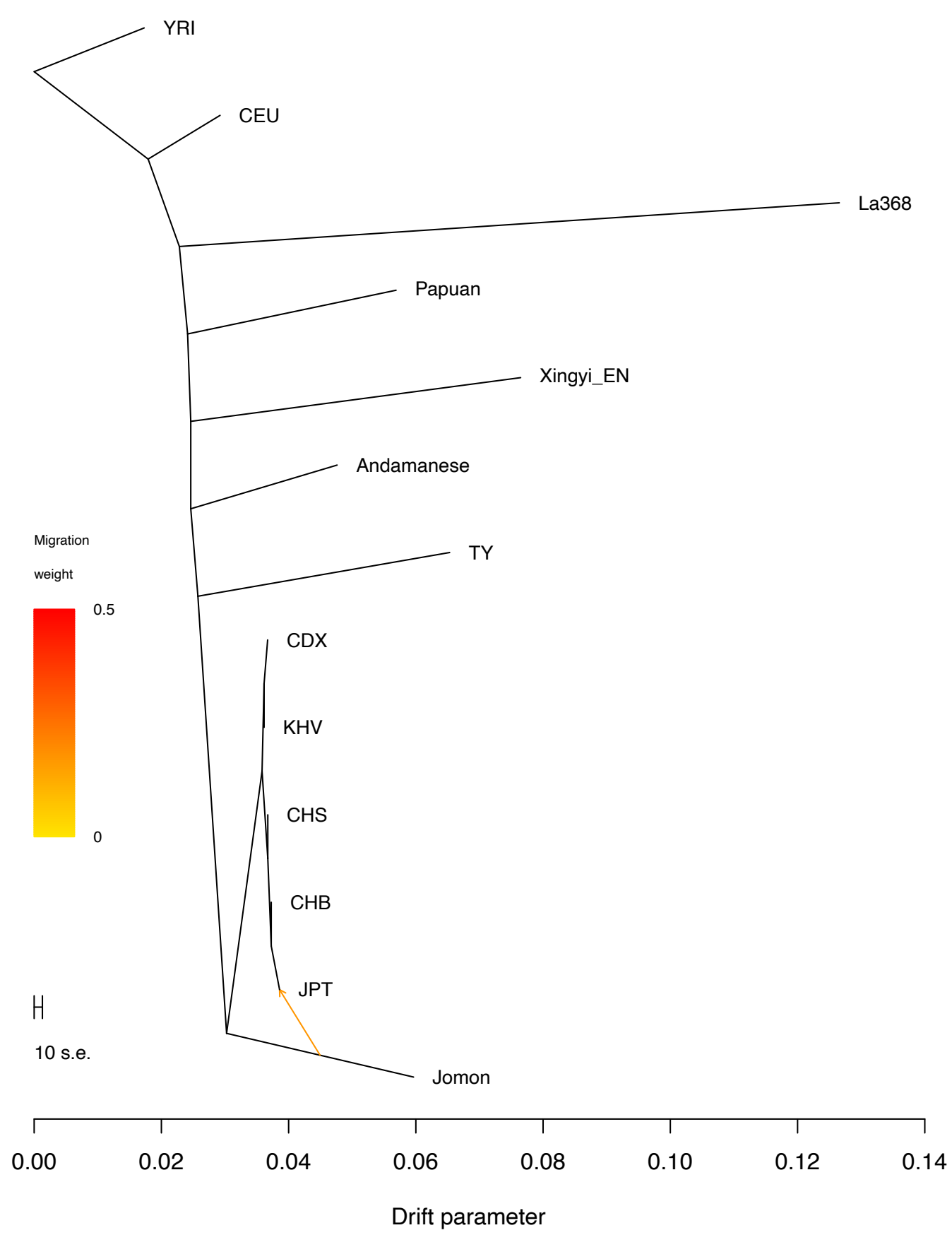
YRI
CEU
La368
Papuan
Xingyi_EN
Andamanese
TY
CDX
KHV
CHS
CHB
JPT
Jomon
Migration
weight
0.5
0
10 s.e.
0.00
0.02
0.04
0.06
0.08
0.10
0.12
0.14
Drift parameter

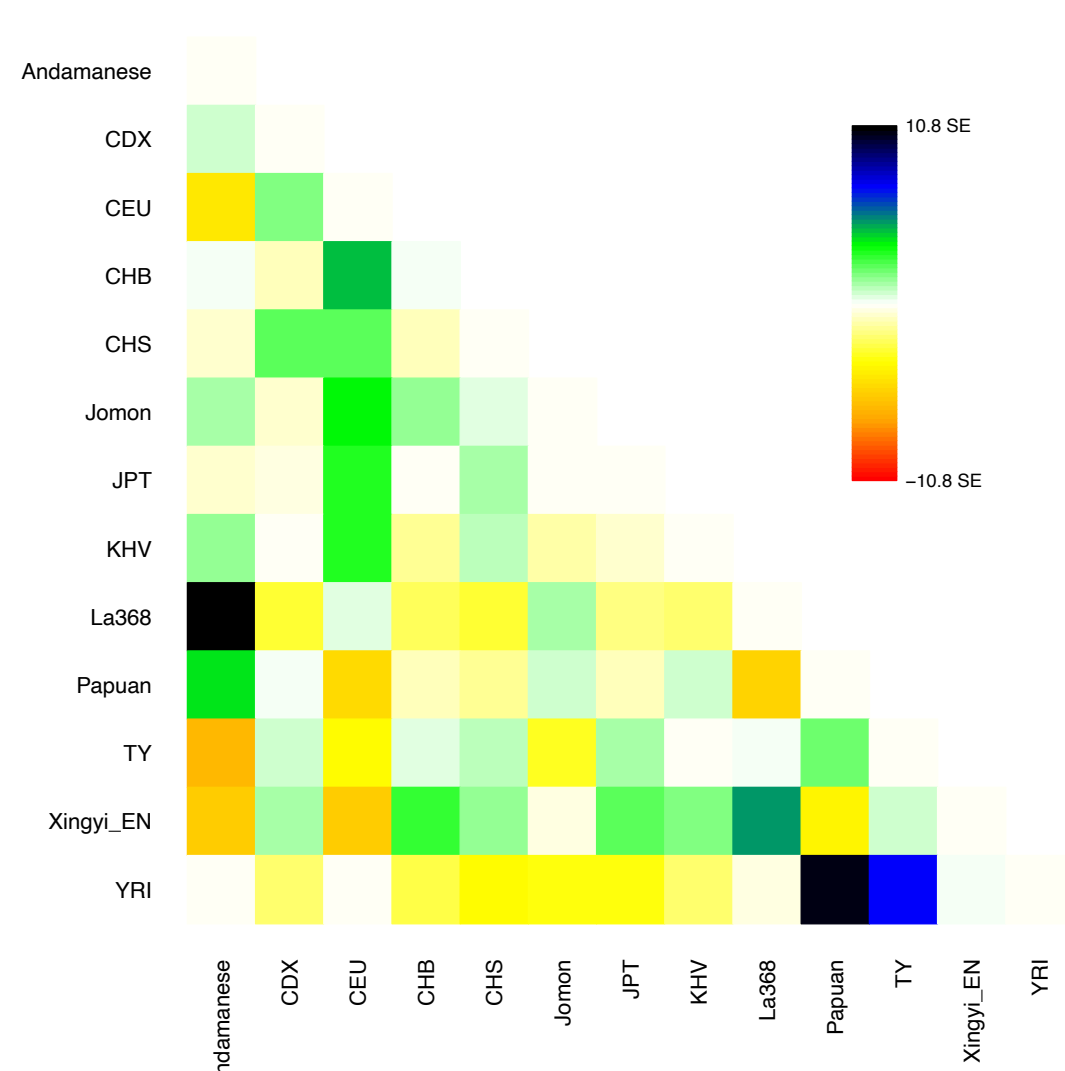
Andamanese
CDX
CEU
CHB
CHS
Jomon
JPT
KHV
La368
Papuan
TY
Xingyi_EN
YRI
10.8 SE
−10.8 SE

Fig. S4. TreeMix topology using 1240K SNPs (224,162 SNPs) with La368 included. Maximum likelihood phylogenetic tree of the basal East Eurasians (Jomon, Tianyuan [TY], XingyiEN, Hoabinhian [La368]) reconstructed by *TreeMix* under a model of one migration. The upper panel shows the inferred tree; the lower panel shows the corresponding residual fit plot. Abbreviations: Japanese in Tokyo, Japan, JPT; Han Chinese in Beijing, China, CHB; Han Chinese South, CHS; Chinese Dai in Xishuangbanna, China, CDX; Kinh in Ho Chi Minh City, Vietnam, KHV; Utah residents (CEPH) with Northern and Western European ancestry, CEU; Yoruba in Ibadan, Nigeria, YRI.

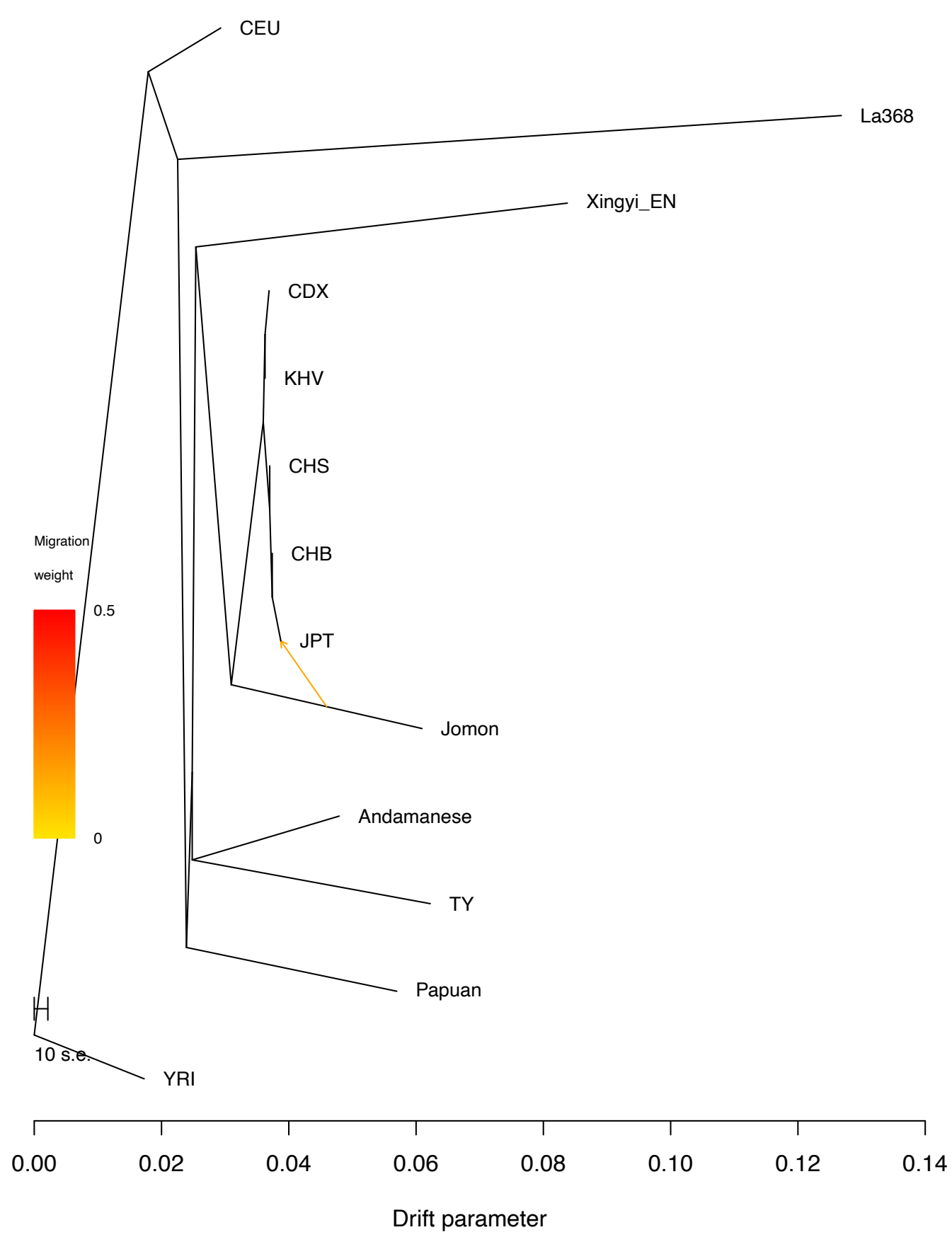

CEU
La368
Xingyi_EN
CDX
KHV
CHS
CHB
JPT
Jomon
Andamanese
TY
Papuan
YRI
Migration weight
0.5
0
10 s.e.
0.00
0.02
0.04
0.06
0.08
0.10
0.12
0.14
Drift parameter

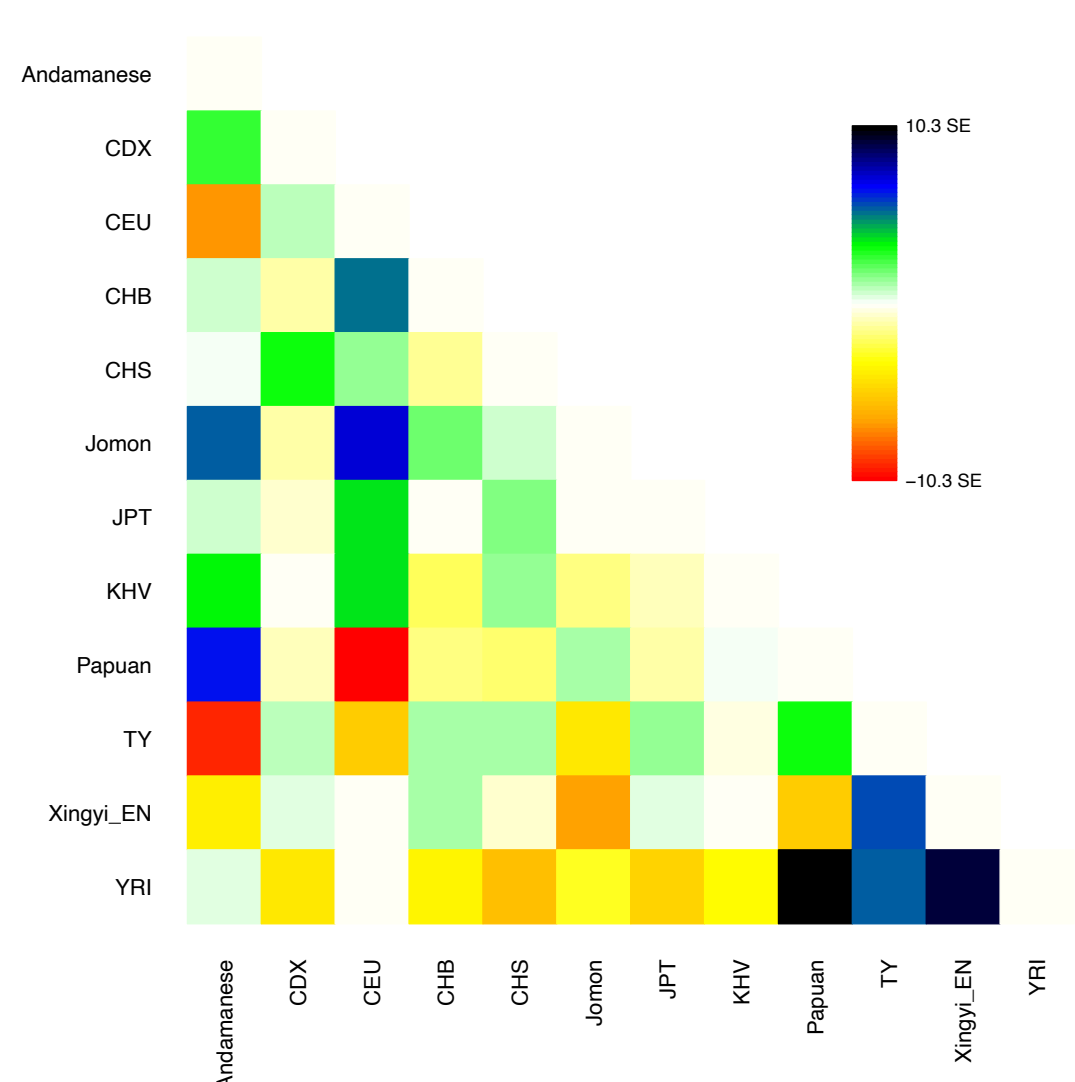

Andamanese
CDX
CEU
CHB
CHS
Jomon
JPT
KHV
Papuan
TY
Xingyi_EN
YRI
10.3 SE
−10.3 SE

Fig. S5. TreeMix topology using 1240K transversions-only (40,735 SNPs) with La368 included. Maximum likelihood phylogenetic tree of the basal East Eurasians (Jomon, Tianyuan [TY], XingyiEN, Hoabinhian [La368]) reconstructed by *TreeMix* under a model of one migration. The upper panel shows the inferred tree; the lower panel shows the corresponding residual fit plot. Abbreviations: Japanese in Tokyo, Japan, JPT; Han Chinese in Beijing, China, CHB; Han Chinese South, CHS; Chinese Dai in Xishuangbanna, China, CDX; Kinh in Ho Chi Minh City, Vietnam, KHV; Utah residents (CEPH) with Northern and Western European ancestry, CEU; Yoruba in Ibadan, Nigeria, YRI.

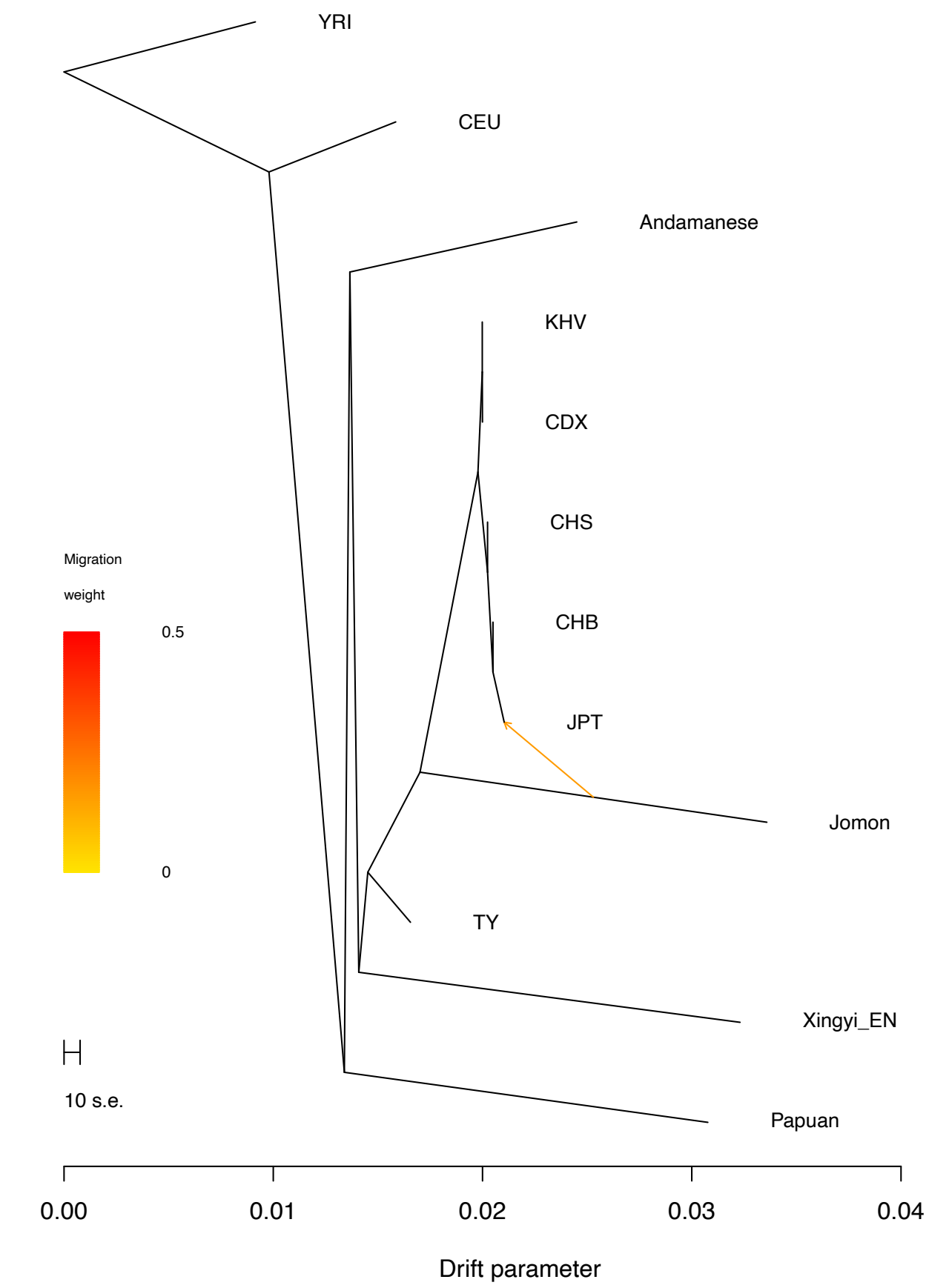
YRI
CEU
Andamanese
KHV
CDX
CHS
CHB
JPT
Jomon
TY
Xingyi_EN
Papuan
Migration
weight
0.5
0
10 s.e.
0.00
0.01
0.02
0.03
0.04
Drift parameter

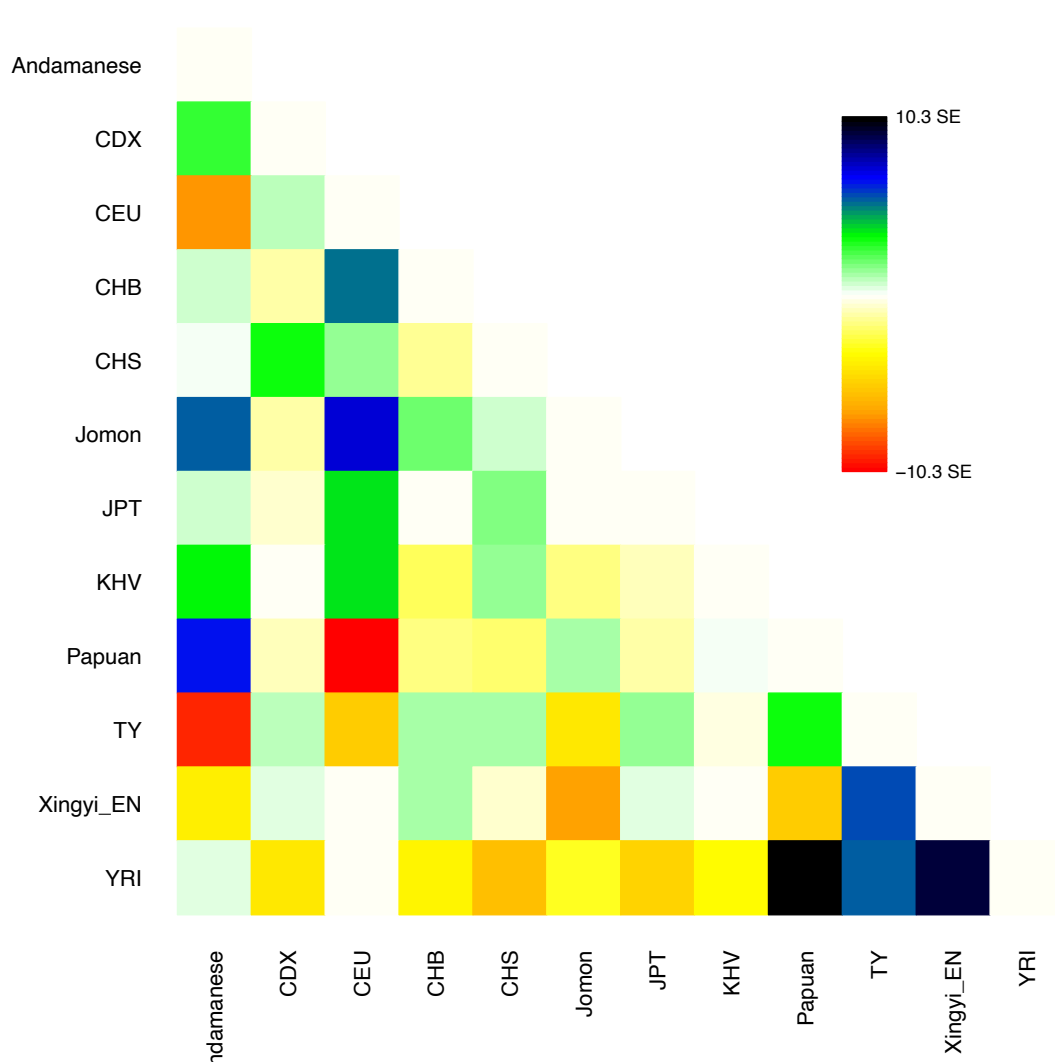
Andamanese
CDX
CEU
CHB
CHS
Jomon
JPT
KHV
Papuan
TY
Xingyi_EN
YRI
10.3 SE
−10.3 SE

Fig. S6. TreeMix topology using 1240K SNPs (582,535 SNPs) with La368 excluded. Maximum likelihood phylogenetic tree of the basal East Eurasians (Jomon, Tianyuan [TY], XingyiEN) reconstructed by *TreeMix* under a model of one migration. The upper panel shows the inferred tree; the lower panel shows the corresponding residual fit plot. Abbreviations: Japanese in Tokyo, Japan, JPT; Han Chinese in Beijing, China, CHB; Han Chinese South, CHS; Chinese Dai in Xishuangbanna, China, CDX; Kinh in Ho Chi Minh City, Vietnam, KHV; Utah residents (CEPH) with Northern and Western European ancestry, CEU; Yoruba in Ibadan, Nigeria, YRI.

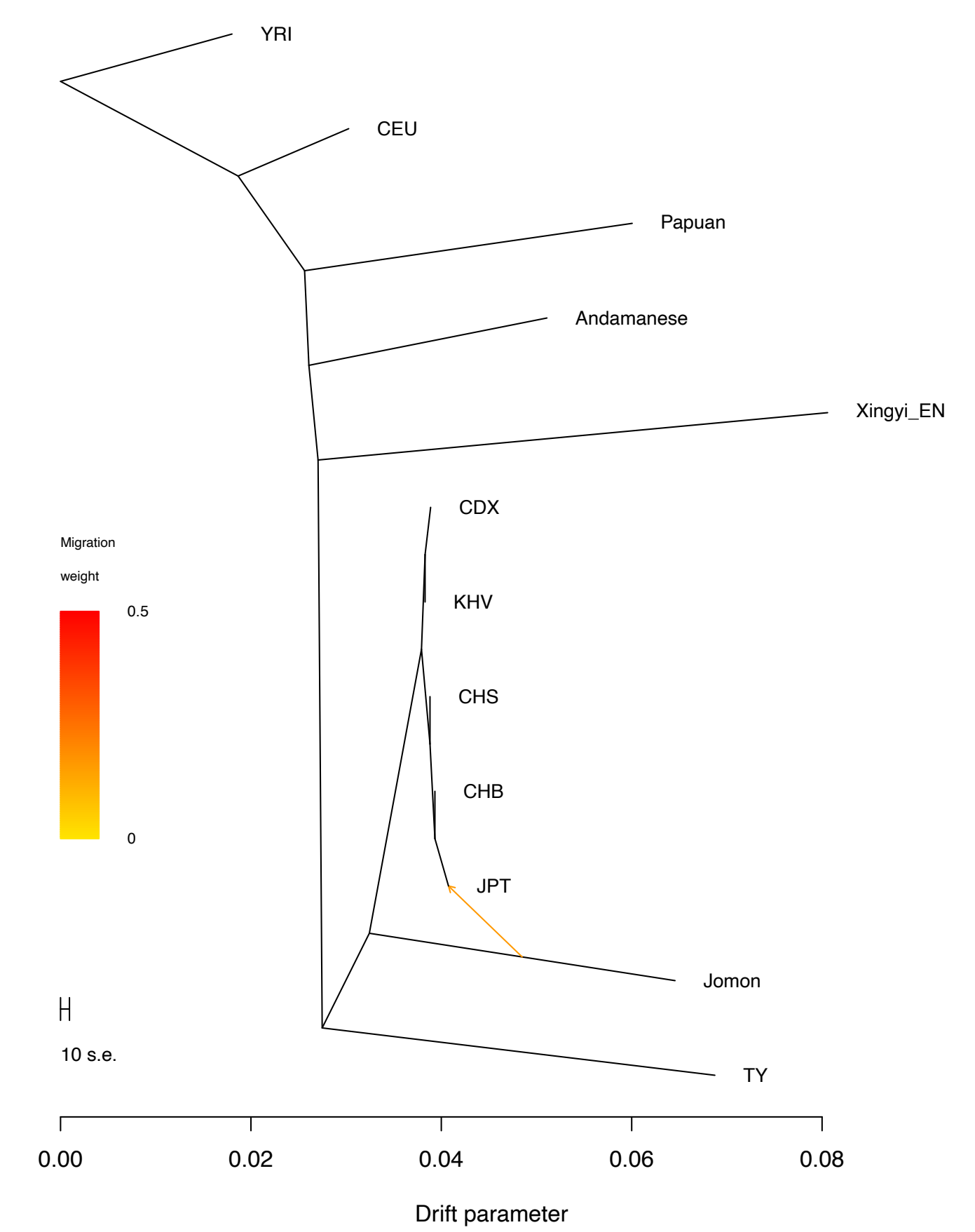
YRI
CEU
Papuan
Andamanese
Xingyi_EN
CDX
KHV
CHS
CHB
JPT
Jomon
TY
Migration
weight
0.5
0
10 s.e.
0.00
0.02
0.04
0.06
0.08
Drift parameter

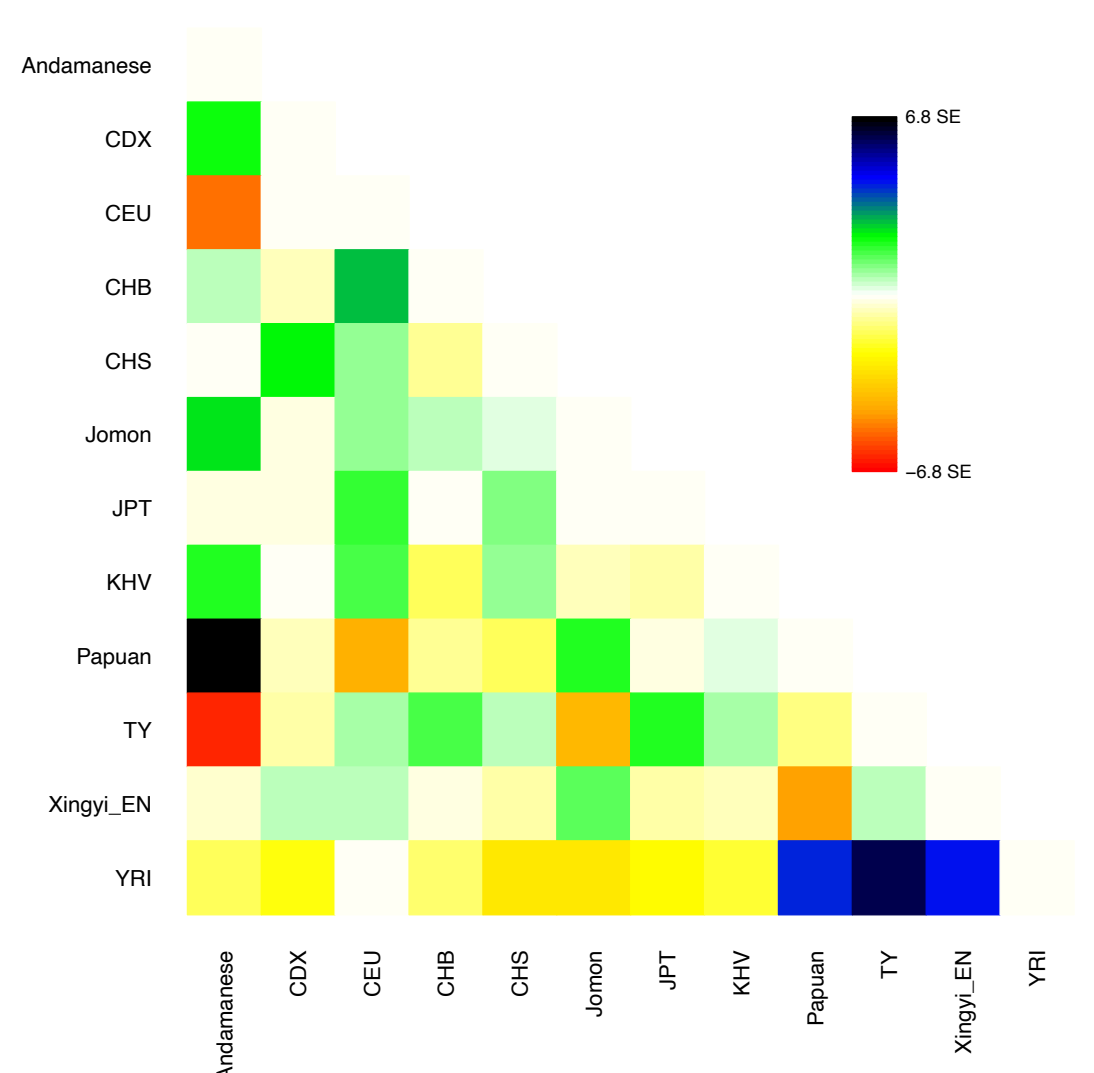
Andamanese
CDX
CEU
CHB
CHS
Jomon
JPT
KHV
Papuan
TY
Xingyi_EN
YRI
6.8 SE
−6.8 SE

Fig. S7. **TreeMix topology using 1240K transversions-only (242,353 SNPs) with La368 excluded.** Maximum likelihood phylogenetic tree of the basal East Eurasians (Jomon, Tianyuan [TY], XingyiEN) reconstructed by *TreeMix* under a model of one migration. The upper panel shows the inferred tree; the lower panel shows the corresponding residual fit plot. Abbreviations: Japanese in Tokyo, Japan, JPT; Han Chinese in Beijing, China, CHB; Han Chinese South, CHS; Chinese Dai in Xishuangbanna, China, CDX; Kinh in Ho Chi Minh City, Vietnam, KHV; Utah residents (CEPH) with Northern and Western European ancestry, CEU; Yoruba in Ibadan, Nigeria, YRI.

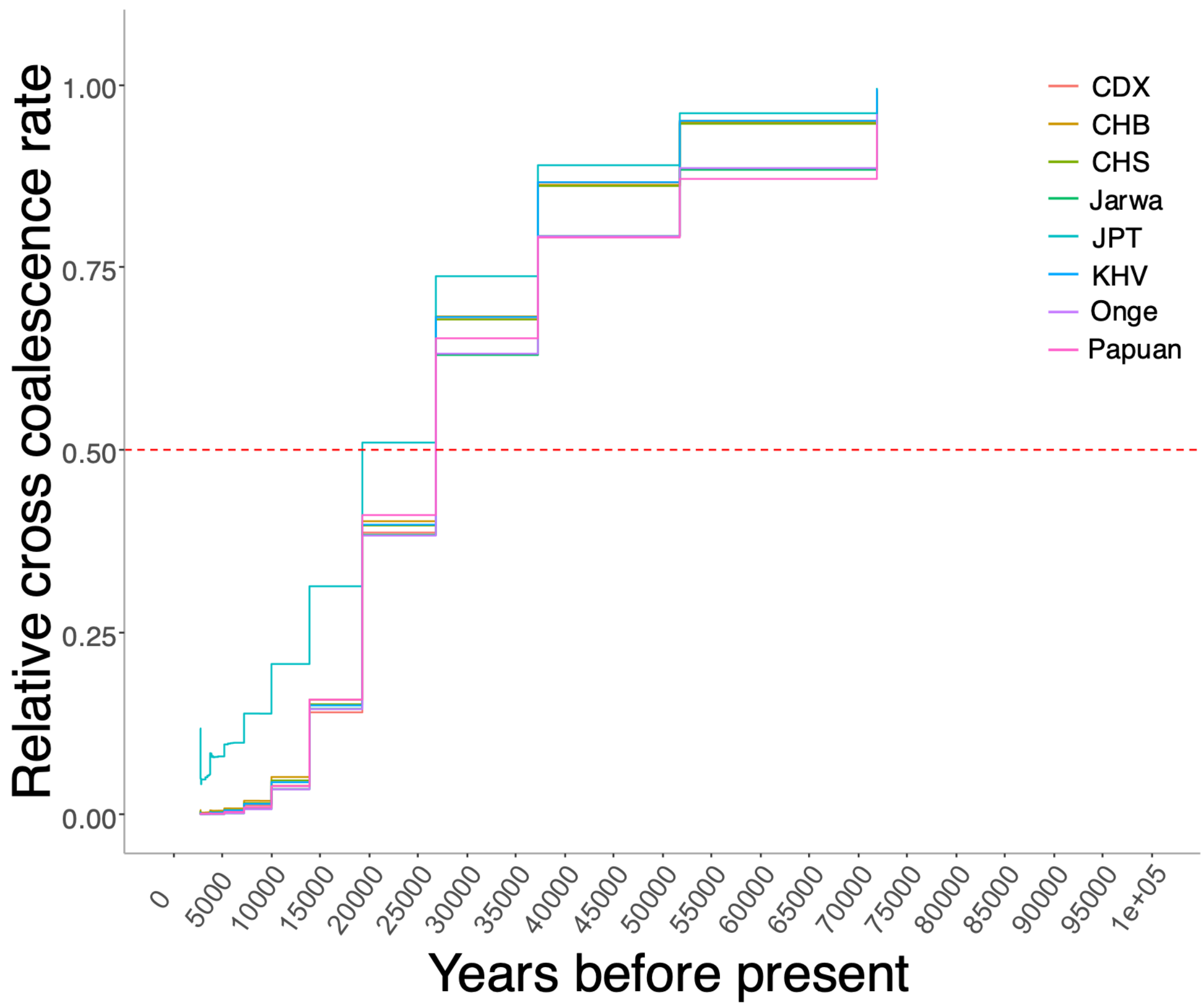

Fig. S8. Relative cross coalescence rate (RCCR) between the Jomon lineage and modern 1KG East Asians, SGDP Papuans and Andamanese from Mondal et al. (2016) (*97*)**.** RCCR is calculated between pairs of populations, where a generation time at an RCCR of 0.5 indicates the divergence time between the two focal populations.

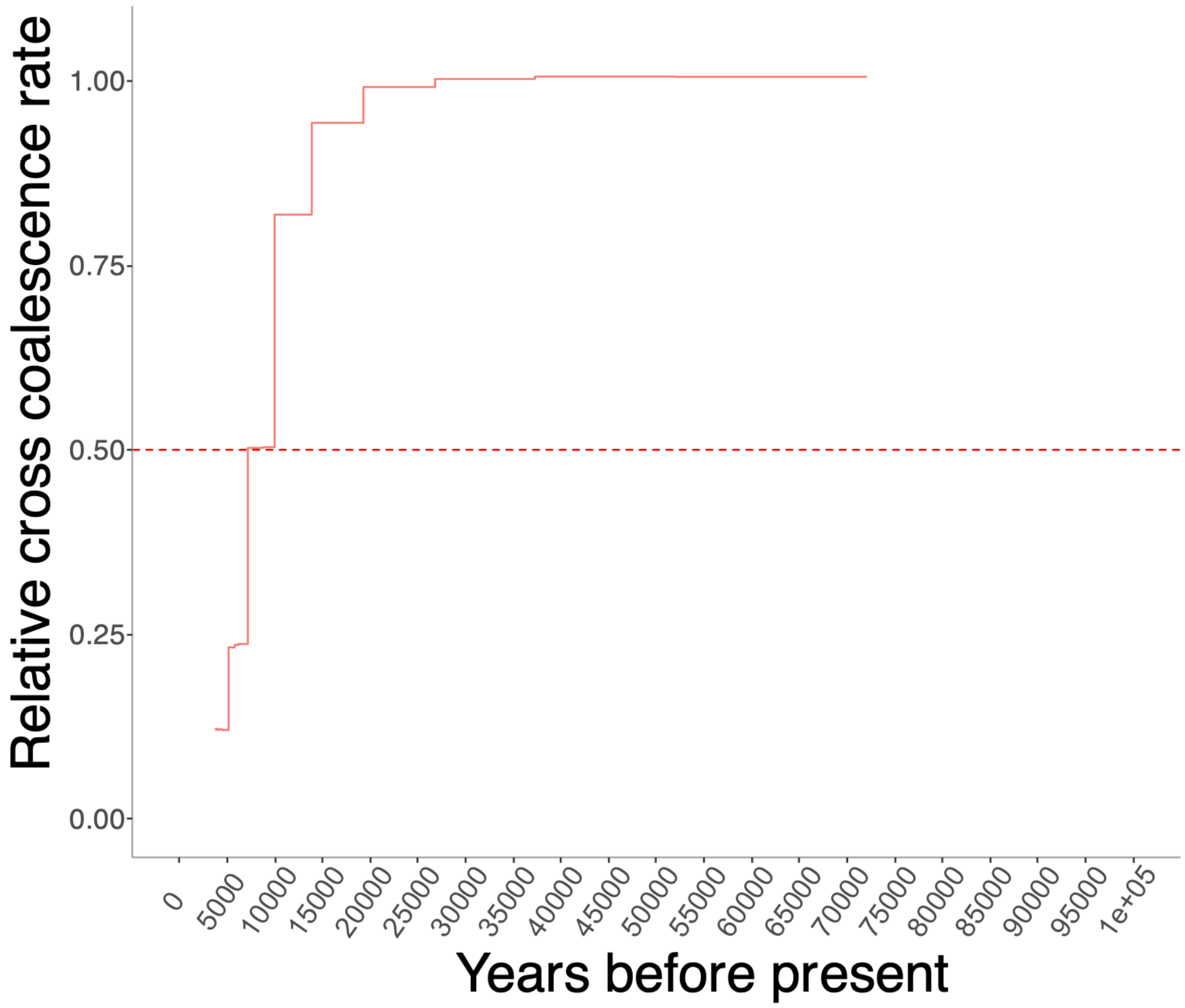

Fig. S9. Relative cross coalescence rate (RCCR) between the Hondo and Hokkaido Jomon.

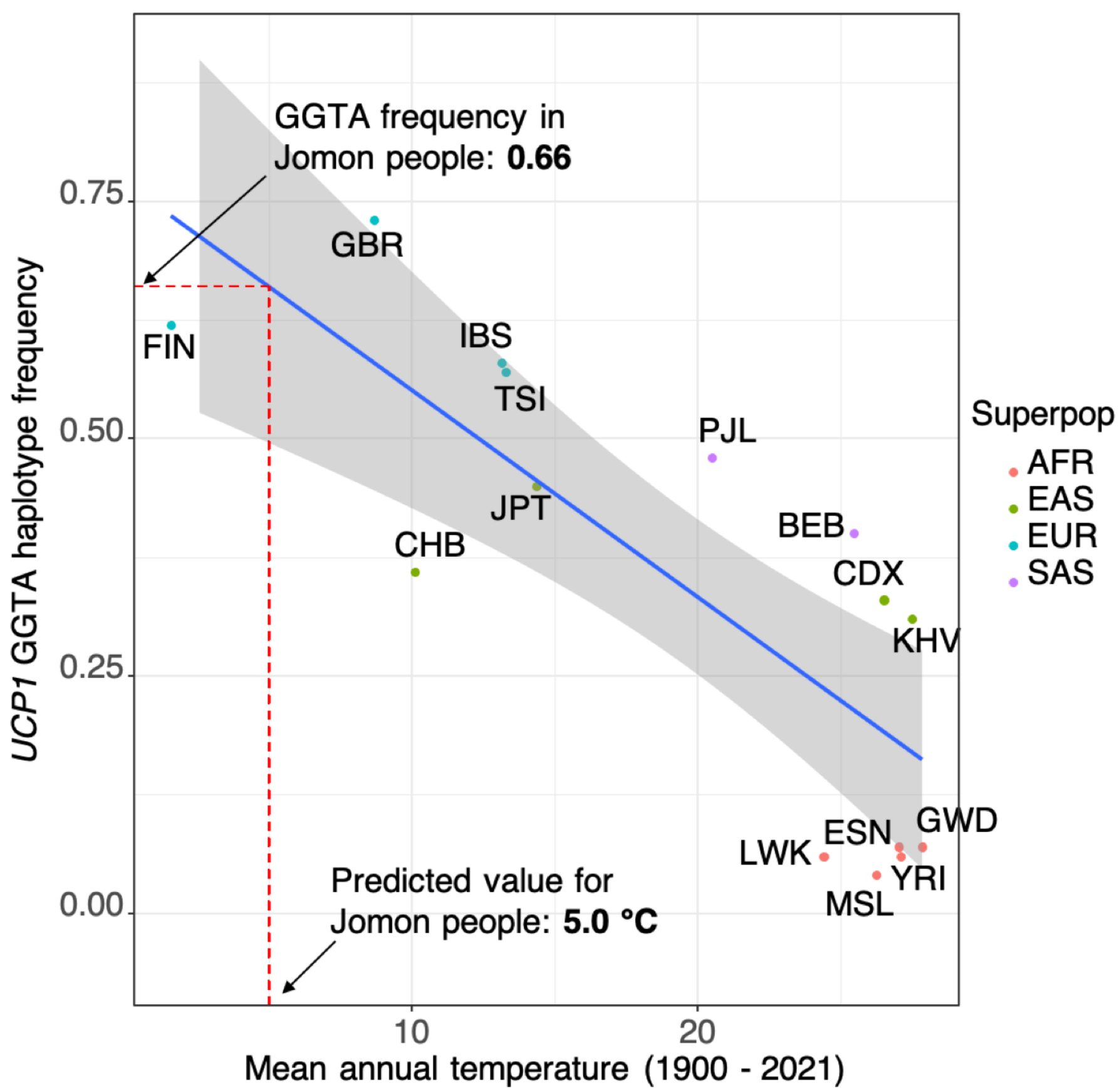

Fig. S10. Replication of the spatial association between the *UCP1* GGTA haplotype and mean annual temperature reported in Nishimura et al. (2017) (*48*)**.** Blue line and grey band represent a regression line and its 95% confidence interval, which were computed with the exception of the Jomon. The abbreviations used are the following: African (AFR): ESN (Esan in Nigeria), GWD (Gambian in Western Divisions in the Gambia), LWK (Luhya in Webuye, Kenya), MSL (Mende in Sierra Leone), YRI (Yoruba in Ibadan, Nigeria); European (EUR): FIN (Finnish in Finland), GBR (British in England and Scotland), IBS (Iberian Population in Spain), TSI (Toscani in Italia); East Asian (EAS): CDX (Chinese Dai in Xishuangbanna, China), CHB (Han Chinese in Beijing, China), JPT (Japanese in Tokyo, Japan), KHV (Kinh in Ho Chi Minh City, Vietnam); South Asian (SAS): BEB (Bengali from Bangladesh), PJL (Punjabi from Lahore, Pakistan).

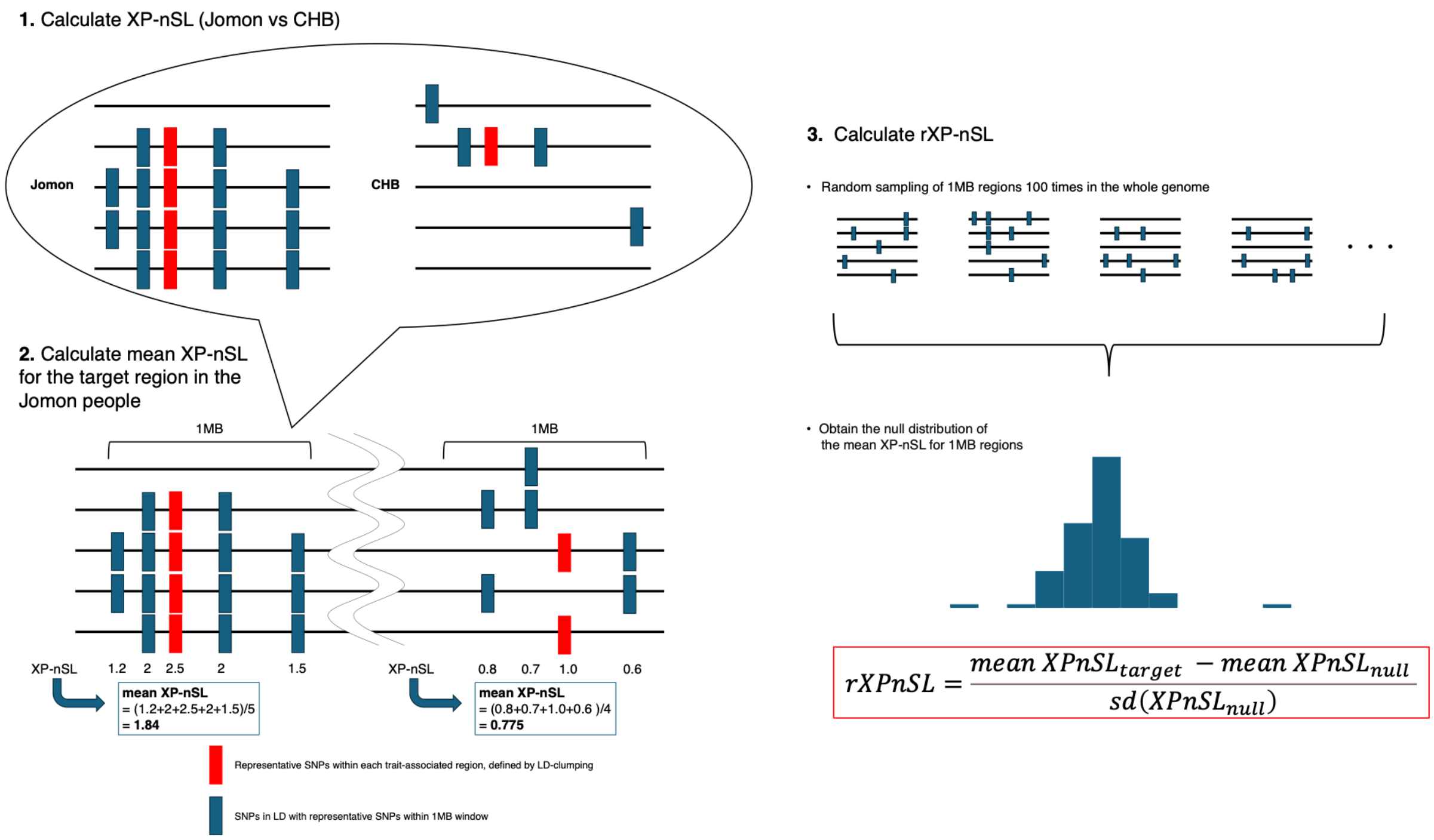

Fig. S11. Overview of genome-wide selection scan methods of this study. We executed LD-clumping for 1KG JPT modern Japanese genomes to determine the representative SNP (red) and other trait-associated SNPs (blue) of each trait-associated region.

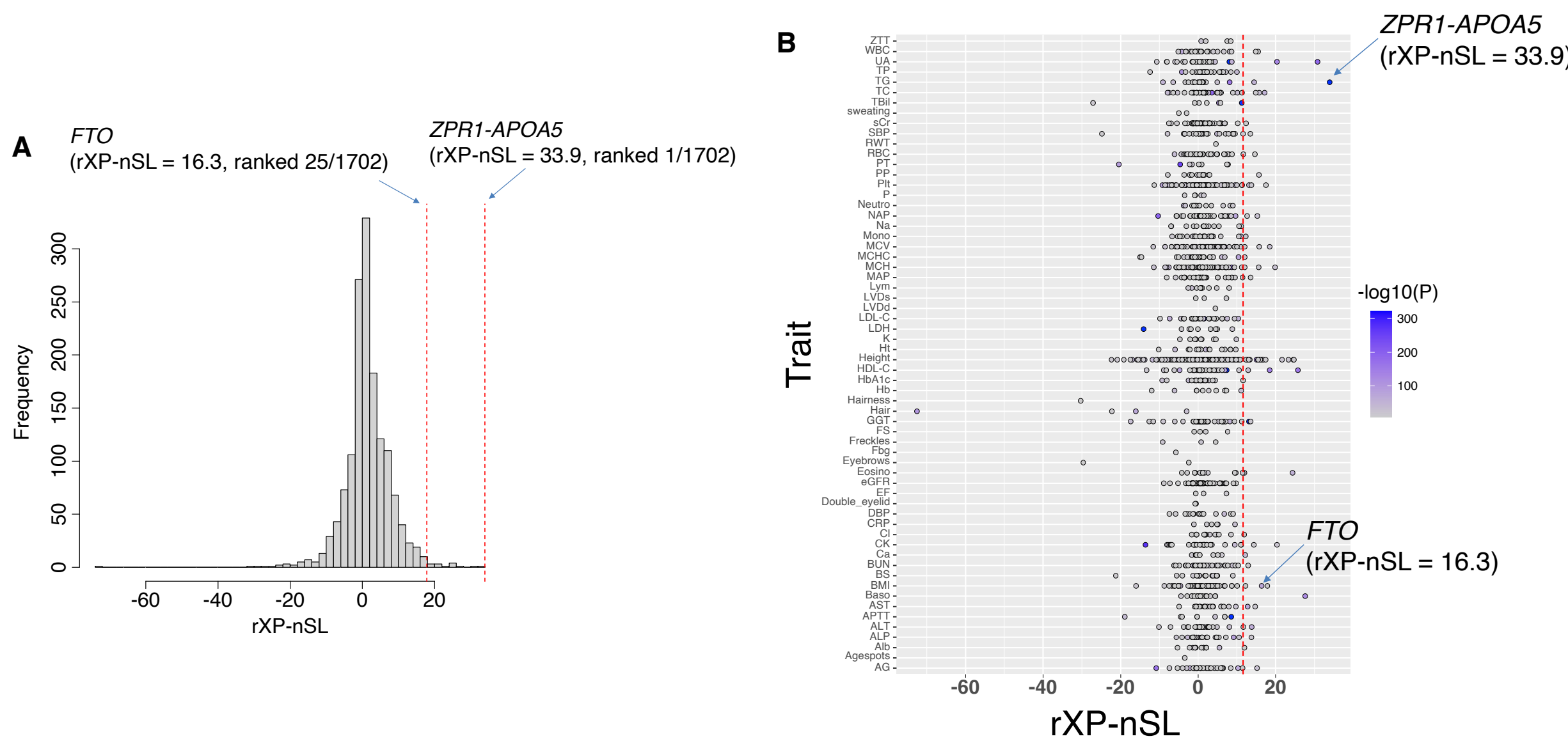

Fig. S12. rXP-nSL values for trait-associated regions which were detected by previous genome-wide association studies (GWAS). (A) Histogram of rXP-nSL values of 1,702 trait-associated regions. Red dashed vertical lines indicate the rXP-nSL values for the *FTO* and *APOA5* regions. (B) rXP-nSL values for trait-associated regions of each trait. The vertical axis represents traits cited from GWAS targeting modern Japanese individuals in previous studies (*51*, *52*, *104*, *105*). Each point represents a trait-associated region. The color of each point is displayed darker for lower P-values in the previous GWAS. The 95th percentile of the empirical distribution is shown as red dashed vertical line (rXP-nSL = 11.25).

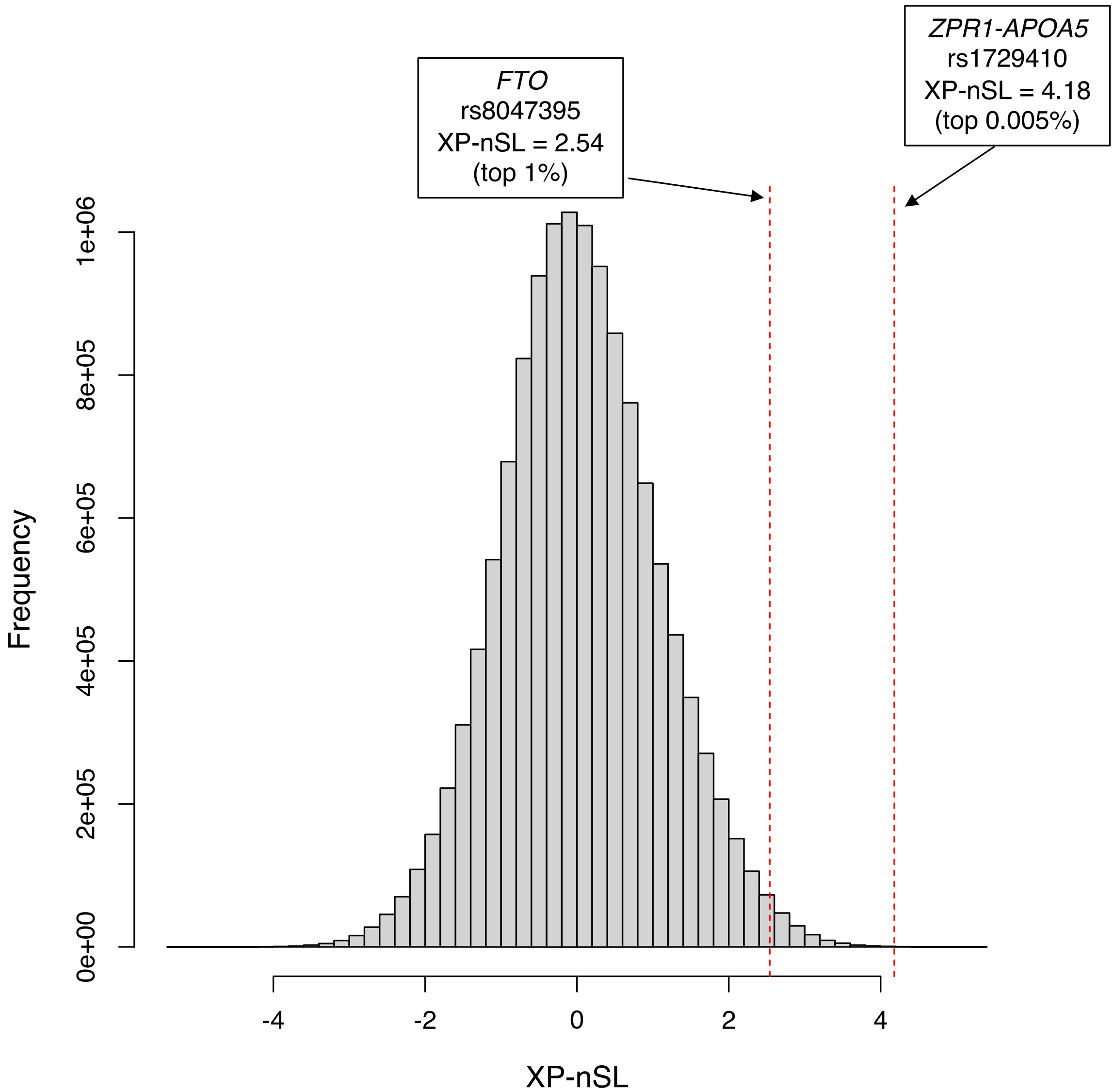

Fig. S13. Histogram of XP-nSL values for 13 million SNPs comparing the Jomon with the CHB population.

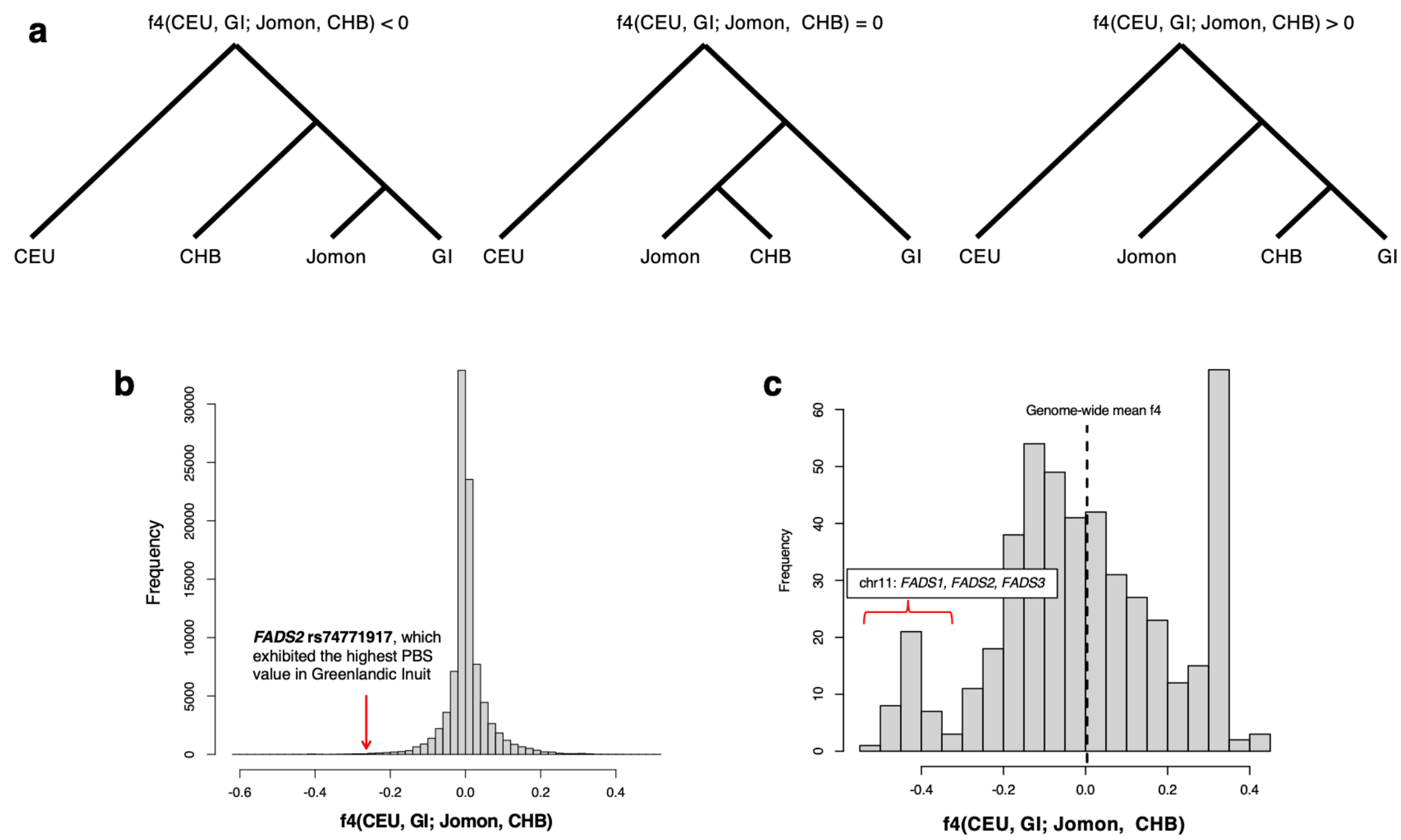

Fig. S14 Histogram of f_4(CEU, Greenlandic Inuit; Jomon, CHB) for SNPs with significant PBS values in Greenlandic Inuit (GI) in Fumagalli et al. (2015) (*56*). We conducted the f_4 test to clarify whether the allele frequencies in the Jomon are similar to those in the GI, regarding SNPs with positive natural selection signals in the GI (*56*). (A) Supported topologies of the Jomon, GI and CHB for various f_4 values. (B) Histogram of f_4(CEU, GI; Jomon, CHB) for 94,577 whole genome SNPs. The red arrow shows the f_4(CEU, GI; Jomon, CHB) value of *FADS2* rs74771917, which exhibited the highest PBS value in GI. (C) Histogram of f_4(CEU, GI; Jomon, CHB) for SNPs with significant PBS values higher than the 99.5th percentile in GI, as reported in the previous study. While most SNPs yielded f_4 values centered around zero—consistent with the closer overall genetic relationship between the Jomon and Han Chinese—the SNP with the lowest f_4 value was located in *FADS2*, indicating that allele frequencies at this locus were more similar between the Jomon and Inuit than between the Jomon and Han Chinese. This pattern was not observed elsewhere in the genome and is unlikely to reflect shared ancestry. Instead, it suggests locus-specific convergence, potentially driven by similar dietary pressures.

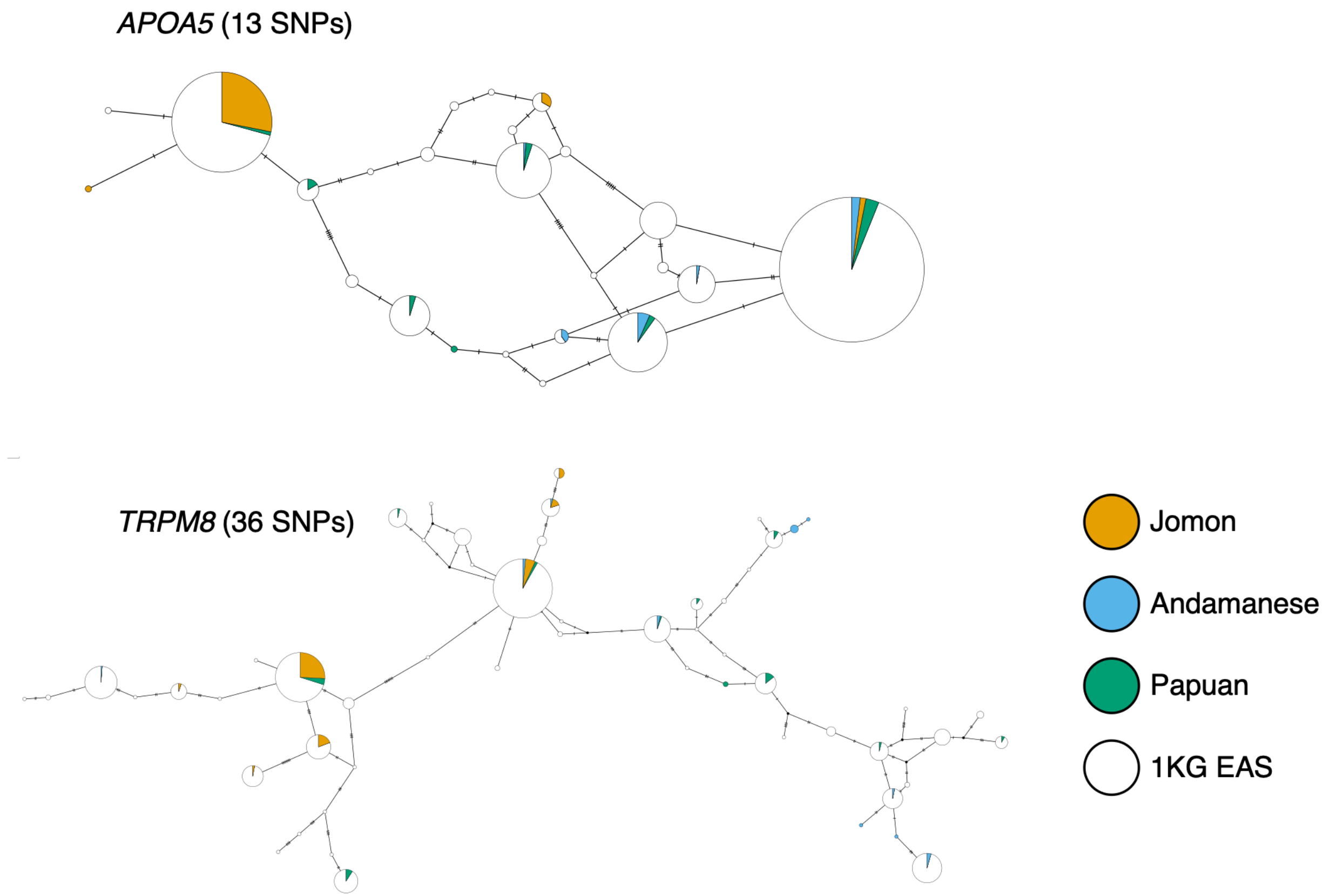

Fig. S15. Haplotype networks of the cold-adaptive loci APOA5 and TRPM8 in Jomon, 1KG East Asians, Andamanese, and Papuans. Each circle represents a unique haplotype, with the size proportional to its frequency in the combined dataset. Pie chart colors indicate the population(s) in which each haplotype is observed: orange = Jomon; blue = Andamanese; green = Papuans; white = 1KG East Asians (CHB, CHS, CDX, JPT, and KHV).
The gene name and the number of SNPs used for each network are indicated in the upper left of each panel.

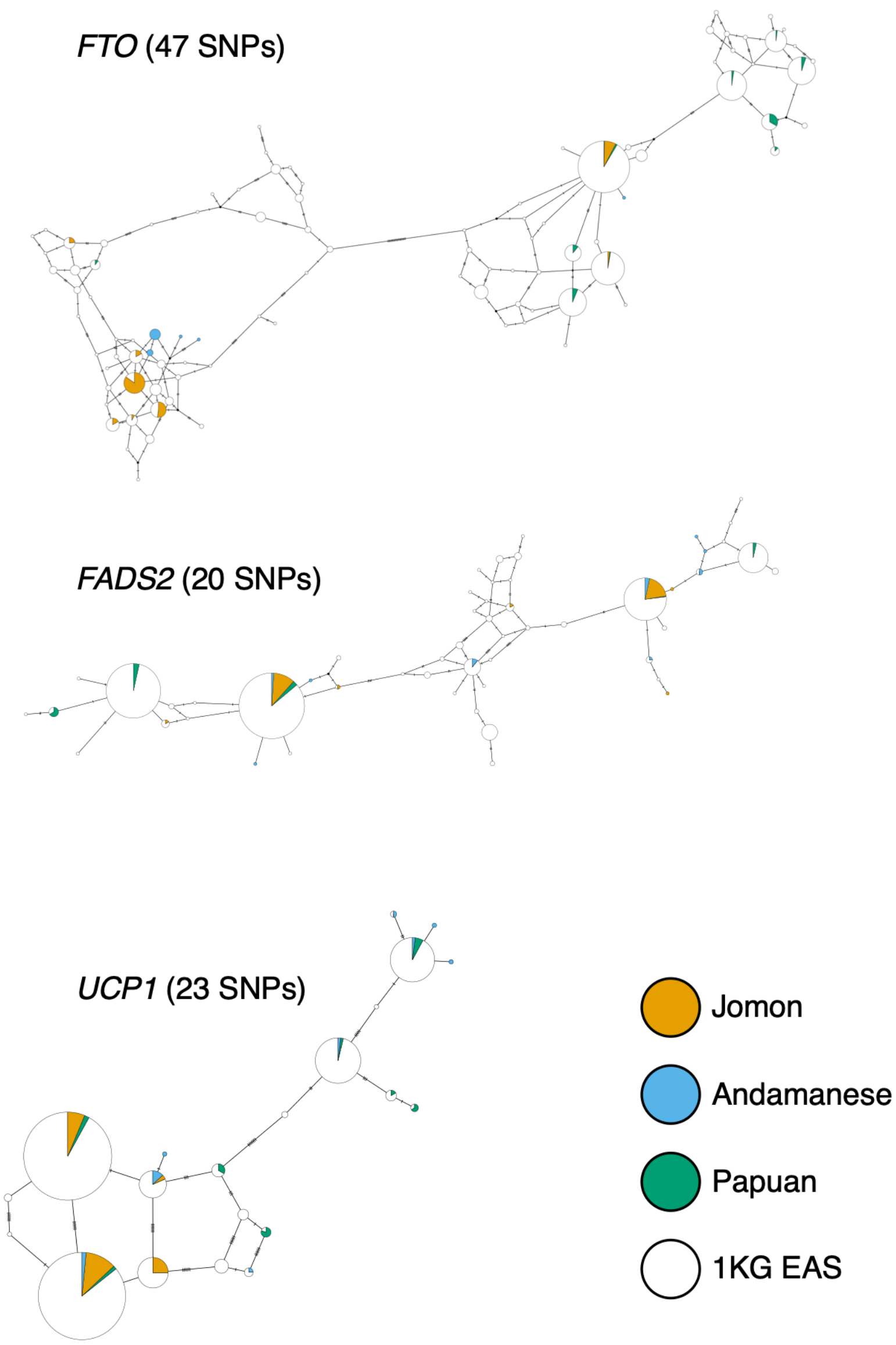

Fig. S16. Haplotype networks of the cold-adaptive loci *FTO*, *FADS2* and *UCP1* in Jomon, 1KG East Asians, Andamanese, and Papuans. Each circle represents a unique haplotype, with the size proportional to its frequency in the combined dataset. Pie chart colors indicate the population(s) in which each haplotype is observed: orange = Jomon; blue = Andamanese; green = Papuans; white = 1KG East Asians (CHB, CHS, CDX, JPT, and KHV).
The gene name and the number of SNPs used for each network are indicated in the upper left of each panel.

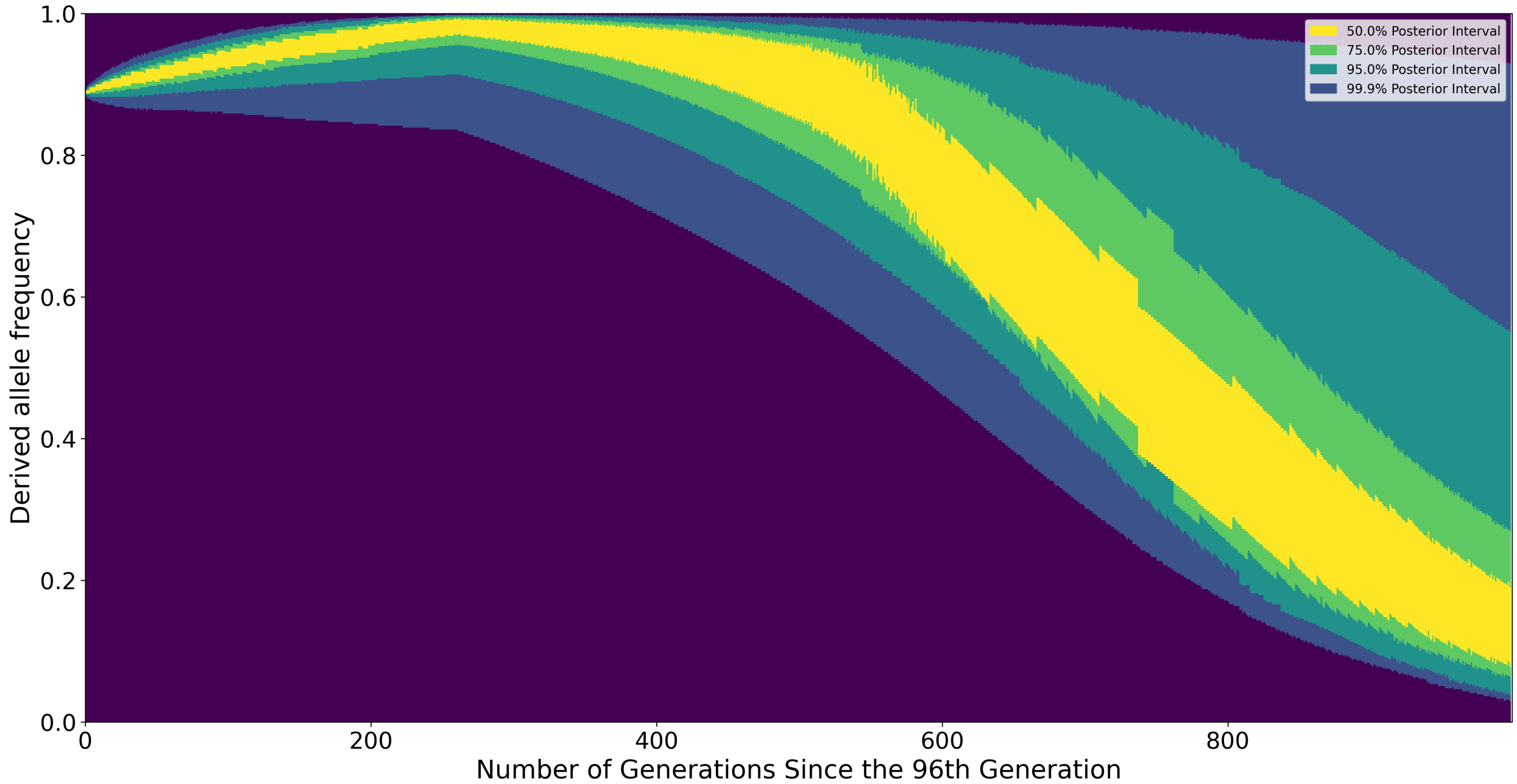

Fig. S17. CLUES2 plot for the Jomon showing the posterior probability of the derived allele frequency trajectory for rs662799. The derived allele for rs662799 in the *ZPR1-APOA5* region elevates TG. Selection coefficients (s) were estimated as 0.00725 during the Last Glacial period (10,000 to 28,000 years before present) and -0.00554 after the Last Glacial period (~10,000 years before present).

Table S1. Newly sequenced sample information.

Table S2. Newly sequenced library information with accession numbers.

Table S3. List of the Jomon individuals from previous studies used in this study.

Table S4. f_4-statistics underlying Fig. 2C. f_4(YRI, Yana; X, Andamanese) and f_4(YRI, Andamanese; X, Yana) for present-day East Asians and Jomon groups. Columns report the estimate (est), standard error (se), Z-score, and P-value for each test.

Table S5. f_4-statistics comparing Hokkaido Jomon to present-day and ancient populations. f_4(YRI, Yana; Hokkaido Jomon, X) for a wide panel of modern and ancient populations. Columns show the estimate (est), standard error (se), Z-score, and P-value for each test.

Table S6 Pairwise distributional differences in Denisovan match rates between the Jomon and other populations. This table summarizes the pairwise Wasserstein distances between the distributions of Denisovan match rates observed in the Jomon and seven other populations (JPT, CHB, CHS, CDX, KHV, Papuan, Andamanese). For each comparison, we report the observed Wasserstein distance and the empirical P-value from a permutation test (10,000 replicates). Significant differences ($P < 0.05$) indicate that the distribution of Denisovan-like archaic segments in the target population significantly deviates from that of the Jomon.

Target Population	Wasserstein distance to Jomon	Permutation test *P* value
JPT	0.032	0.96
CHB	0.029	0.46
CHS	0.034	0.82
CDX	0.033	0.97
KHV	0.058	0.29
Papuan	0.12	0.0049
Andamanese	0.14	0.0069

Table S7. Jomon-specific Denisovan-derived segments of D0 origin shared with continental East Asians

Table S8. Sensitivity of LD-clumping settings: pairwise comparison of region rankings across r^2 and window-size combinations. Pairwise comparisons of LD-clumping parameter settings (r^2 = {0, 0.05, 0.1, 0.2} × window = {250, 500, 1000 kb}). For each pair of settings (A, B), we report the number of genomic regions in common (n common regions), Spearman's rank correlation (ρ) of region-level scores, its P value, the number of overlapping top 5% regions (n top 5%), and the Jaccard index of top 5% sets.

Table S9. Sensitivity analysis using an alternative East Asian GWAS panel. Results of applying the rXP-nSL framework to 1,487 genomic regions using effect sizes from Chen et al. (2023) (*53*) (Taiwanese GWAS; n = 92,615). Both *APOA5* and *FTO* loci appear among the top candidates, confirming robustness to the choice of East Asian GWAS panel.

Locus	GWAS Dataset	Reference	rXP-nSL	Rank
APOA5	Japanese GWAS	Kanai et al. (2018) *(52)*	33.9	1/1702
APOA5	Taiwanese GWAS	Chen et al. (2023) *(53)*	25.1	5/1487
FTO	Japanese GWAS	Akiyama et al. (2017) (51)	16.3	25/1702
FTO	Taiwanese GWAS	Chen et al. (2023) *(53)*	9.79	55/1487

Table S10. Allele frequencies, PBS in Greenlandic Inuit (GI) and f_4(CEU, GI; Jomon, CHB) for SNPs with significant PBS values higher than the 99.5th percentile in GI, as reported in Fumagalli et al. (2015) (*56*)**.** Allele frequencies in GI and PBS values were cited from the previous study (*56*).

Table S11. Derived allele frequencies of cold-adaptive variants in Jomon, Andamanese, Ancient Siberian and North American.

Table S12. CLUES2 inference for 18 *APOA5* SNPs. CLUES2 results for 18 triglyceride-associated SNPs (GWAS $p < 10^{-200}$) within the *APOA5* locus, analyzed using Relate-derived genealogies. Shown are the posterior mean selection coefficient (s), log-likelihood ratio (LLR), inferred selection onset time, and other summary statistics for each SNP.

REFERENCES

1. C. Higham, Hunter-gatherers in Southeast Asia: From prehistory to the present. *Hum. Biol.* **85**, 21–43 (2013).

2. K. E. Westaway, J. Louys, R. D. Awe, M. J. Morwood, G. J. Price, J. X. Zhao, M. Aubert, R. Joannes-Boyau, T. M. Smith, M. M. Skinner, T. Compton, R. M. Bailey, G. D. Van Den Bergh, J. De Vos, A. W. G. Pike, C. Stringer, E. W. Saptomo, Y. Rizal, J. Zaim, W. D. Santoso, A. Trihascaryo, L. Kinsley, B. Sulistyanto, An early modern human presence in Sumatra 73,000–63,000 years ago. *Nature* **548**, 322–325 (2017).

3. T. Goebel, The missing years for modern humans. *Science* **315**, 194–196 (2007).

4. Y. Kaifu, M. Izuho, T. Goebel, "Modern human dispersal and behavior in Paleolithic Asia: Summary and discussion," in *Emergence and Diversity of Modern Human Behavior in Paleolithic Asia*, Y. Kaifu, M. Izuho, T. Goebel, H. Sato, A. Ono, Eds. (Texas A&M Univ. Press, 2015), pp. 535–566.

5. R. Dennell, *From Arabia to the Pacific: How Our Species Colonised Asia* (Routledge, 2020).

6. T. Gakuhari, S. Nakagome, S. Rasmussen, M. E. Allentoft, T. Sato, B. N. Chuinneagáin, H. Matsumae, K. Koganebuchi, R. Schmidt, S. Mizushima, O. Kondo, N. Shigehara, M. Yoneda, Ancient Jomon genome sequence analysis sheds light on migration patterns of early East Asian. *Commun. Biol.* **3**, 437 (2020).

7. H. McColl, F. Racimo, L. Vinner, F. Demeter, T. Gakuhari, J. V. Moreno-Mayar, G. van Driem, U. Gram Wilken, A. Seguin-Orlando, C. de la Fuente Castro, S. Wasef, R. Shoocongdej, V. Souksavatdy, T. Sayavongkhamdy, M. M. Saidin, M. E. Allentoft, T. Sato, A.-S. Malaspinas, F. A. Aghakhanian, T. Korneliussen, A. Prohaska, A. Margaryan, P. de Barros Damgaard, S. Kaewsutthi, P. Lertrit, T. M. H. Nguyen, H. Hung, T. Minh Tran, H. Nghia Truong, G. H. Nguyen, S. Shahidan, K. Wiradnyana, H. Matsumae, N. Shigehara, M. Yoneda, H. Ishida, T. Masuyama, Y. Yamada, A. Tajima, H. Shibata, A. Toyoda, T. Hanihara, S. Nakagome, T. Deviese, A.-M. Bacon, P. Duringer, J.-L. Ponche, L. Shackelford, E. Patole-Edoumba, A. T. Nguyen, B. Bellina-Pryce, J.-C. Galipaud, R. Kinaston, H. Buckley, C. Pottier, S. Rasmussen, T. Higham, R. A. Foley, M. M. Lahr, L. Orlando, M. Sikora, M. E. Phipps, H. Oota, C.

Higham, D. M. Lambert, E. Willerslev, The prehistoric peopling of Southeast Asia. *Science* **361**, 88–92 (2018).

8. J.-C. Launay, G. Savourey, Cold Adaptations. *Ind. Health* **47**, 221–227 (2009).

9. A. A. Vasilevsky, Periodization and classification of the Upper Paleolithic of Sakhalin and Hokkaido in the light of the research conducted at the Ogonki-5 site. *Archaeol. Ethnol. Anthropol. Eurasia* **3**, 51–69 (2003).

10. M. Izuho, “Human technological and behavioral adaptation to landscape changes around the Last Glacial Maximum in Japan: A focus on Hokkaido,” in *Paleoamerican Odyssey*, K. E. Graf, C. V. Ketron, M. R. Waters, Eds. (Texas A&M Univ. Press, 2014), pp. 45–64.

11. M. Izuho, Y. Kaifu, “The appearance and characteristics of the early Upper Palaeolithic in the Japanese Archipelago,” in *Emergence and Diversity of Modern Human Behavior in Paleolithic Asia*, Y. Kaifu, M. Izuho, T. Goebel, H. Sato, A. Ono, Eds. (Texas A&M Univ. Press, 2015), pp. 289–313.

12. K. Morisaki, K. Sano, M. Izuho, Early Upper Paleolithic blade technology in the Japanese Archipelago. *Archaeol. Res. Asia* **17**, 79–97 (2019).

13. P. U. Clark, A. S. Dyke, J. D. Shakun, A. E. Carlson, J. Clark, B. Wohlfarth, J. X. Mitrovica, S. W. Hostetler, A. M. McCabe, The Last Glacial Maximum. *Science* **325**, 710–714 (2009).

14. H. Kanzawa-Kiriyama, K. Kryukov, T. A. Jinam, K. Hosomichi, A. Saso, G. Suwa, S. Ueda, M. Yoneda, A. Tajima, K. Shinoda, I. Inoue, N. Saitou, A partial nuclear genome of the Jomons who lived 3000 years ago in Fukushima, Japan. *J. Hum. Genet.* **62**, 213–221 (2017).

15. L. Speidel, M. Forest, S. Shi, S. R. Myers, A method for genome-wide genealogy estimation for thousands of samples. *Nat. Genet.* **51**, 1321–1329 (2019).

16. M. Izuho, D. Kunikita, Y. Nakazawa, N. Oda, K. Hiromatsu, O. Takahashi, New AMS dates from the Shukubai-Kaso Site (Loc. Sankakuyama), Hokkaido (Japan): Refining the chronology of small flake-based assemblages during the early upper paleolithic in the Paleo-Sakhalin-Hokkaido-Kuril Peninsula. *PaleoAmerica* **4**, 134–150 (2018).

17. D. Natsuki, Migration and adaptation of Jomon people during Pleistocene/Holocene transition period in Hokkaido, Japan. *Quat. Int.* **608-609**, 49–64 (2022).

18. K. Morisaki, M. Izuho, K. Terry, H. Sato, Lithics and climate: Technological responses to landscape change in Upper Palaeolithic northern Japan. *Antiquity* **89**, 554–572 (2015).

19. P. Zhang, R. Haas, C. Paine, X. Zhang, N. Zwyns, A cold habitat: Mapping blade assemblages between the Siberian Altai and the Tibetan Plateau during MIS 3. *J. Paleolit. Archaeol.* **7**, 11 (2024).

20. M. Izuho, The nature of the transition from Late Upper Paleolithic to Incipient Jomon in the Japanese Archipelago: An archaeological perspective. *Anthropol. Pap. Univ. Alaska* **7**, 122–135 (2023).

21. F. Iizuka, The timing and behavioral context of the late-pleistocene adoption of ceramics in greater east and northeast asia and the first people (Without Pottery) in the americas. *PaleoAmerica* **4**, 267–324 (2018).

22. L. G. Davis, D. B. Madsen, The coastal migration theory: Formulation and testable hypotheses. *Quat. Sci. Rev.* **249**, 106605 (2020).

23. I. Buvit, K. Terry, M. Izuho, "Pathways along the Pacific: Using early stone tools to. Reconstruct coastal migration between Japan and the Americas," in *Palaeolandscapes in Archaeology*, M. T. Carson, Ed. (Routledge, 2021), pp. 39–81.

24. M. Izuho, K. Hayashi, Y. Nakazawa, T. Soda, N. Oda, T. Yamahara, M. Kitazawa, I. Buvit, Investigating the eolian context of the Last Glacial Maximum occupation at Kawanishi-C, Hokkaido, Japan. *Geoarchaeology* **29**, 202–220 (2014).

25. I. Buvit, M. Izuho, K. Terry, M. V. Konstantinov, A. V. Konstantinov, Radiocarbon dates, microblades and Late Pleistocene human migrations in the Transbaikal, Russia and the Paleo-Sakhalin-Hokkaido-Kuril Peninsula. *Quat. Int.* **425**, 100–119 (2016).

26. K. Morisaki, "Appearance of Hakuhen-Sentoki (HS points) and second modern human migration into Kyushu, Japan," in *Emergence and Diversity of Modern Human Behavior in*

Paleolithic Asia, Y. Kaifu, M. Izuho, T. Gobel, H. Sato, Eds. (Texas A&M Univ. Press, 2015), pp. 376–388.

27. K. E. Graf, I. Buvit, Human dispersal from Siberia to Beringia: Assessing a Beringian standstill in light of the archaeological evidence. *Curr. Anthropol.* **58**, S583–S603 (2017).

28. M. Rasmussen, S. L. Anzick, M. R. Waters, P. Skoglund, M. Degiorgio, T. W. Stafford, S. Rasmussen, I. Moltke, A. Albrechtsen, S. M. Doyle, G. D. Poznik, V. Gudmundsdottir, R. Yadav, A. S. Malaspinas, V. S. S. White, M. E. Allentoft, O. E. Cornejo, K. Tambets, A. Eriksson, P. D. Heintzman, M. Karmin, T. S. Korneliussen, D. J. Meltzer, T. L. Pierre, J. Stenderup, L. Saag, V. M. Warmuth, M. C. Lopes, R. S. Malhi, S. Brunak, T. Sicheritz-Ponten, I. Barnes, M. Collins, L. Orlando, F. Balloux, A. Manica, R. Gupta, M. Metspalu, C. D. Bustamante, M. Jakobsson, R. Nielsen, E. Willerslev, The genome of a Late Pleistocene human from a Clovis burial site in western Montana. *Nature* **506**, 225–229 (2014).

29. M. Sikora, V. Vladimir, A. Margaryan, P. D. B. Damgaard, C. De Fuente, G. Renaud, M. A. Yang, Q. Fu, I. Dupanloup, K. Giampoudakis, D. Nogués-bravo, C. Rahbek, G. Kroonen, E. Veselovskaya, M. Gerasimova, E. Y. Pavlova, V. G. Chasnyk, P. A. Nikolskiy, V. Andrei, The population history of northeastern Siberia since the Pleistocene. *Nature* **570**, 182–188 (2019).

30. G. R. Scott, D. H. O'Rourke, J. A. Raff, J. C. Tackney, L. J. Hlusko, S. A. Elias, L. Bourgeon, O. Potapova, E. Pavlova, V. Pitulko, J. F. Hoffecker, Peopling the Americas: Not "Out of Japan". *PaleoAmerica* **7**, 309–332 (2021).

31. S. R. Browning, B. L. Browning, Y. Zhou, S. Tucci, J. M. Akey, Analysis of human sequence data reveals two pulses of archaic denisovan admixture. *Cell* **173**, 53–61.e9 (2018).

32. G. S. Jacobs, G. Hudjashov, L. Saag, P. Kusuma, C. C. Darusallam, D. J. Lawson, M. Mondal, L. Pagani, F. X. Ricaut, M. Stoneking, M. Metspalu, H. Sudoyo, J. S. Lansing, M. P. Cox, Multiple deeply divergent denisovan ancestries in papuans. *Cell* **177**, 1010–1021.e32 (2019).

33. F. Chen, F. Welker, C. C. Shen, S. E. Bailey, I. Bergmann, S. Davis, H. Xia, H. Wang, R. Fischer, S. E. Freidline, T. L. Yu, M. M. Skinner, S. Stelzer, G. Dong, Q. Fu, G. Dong, J.

Wang, D. Zhang, J. J. Hublin, A late Middle Pleistocene Denisovan mandible from the Tibetan Plateau. *Nature* **569**, 409–412 (2019).

34. H. Xia, D. Zhang, J. Wang, Z. Fagernäs, T. Li, Y. Li, J. Yao, D. Lin, G. Troché, G. M. Smith, X. Chen, T. Cheng, X. Shen, Y. Han, J. V. Olsen, Z. Shen, Z. Pei, J. J. Hublin, F. Chen, F. Welker, Middle and Late Pleistocene Denisovan subsistence at Baishiya Karst Cave. *Nature* **632**, 108–113 (2024).

35. T. Tsutaya, R. Sawafuji, A. J. Taurozzi, Z. Fagernäs, I. Patramanis, G. Troché, M. Mackie, T. Gakuhari, H. Oota, C. H. Tsai, J. V. Olsen, Y. Kaifu, C. H. Chang, E. Cappellini, F. Welker, A male Denisovan mandible from Pleistocene Taiwan. *Science* **388**, 176–180 (2025).

36. Q. Fu, F. Bai, H. Rao, S. Chen, Y. Ji, A. Liu, E. A. Bennett, F. Liu, Q. Ji, The proteome of the late Middle Pleistocene Harbin individual. *Science* **389**, 704–707 (2025).

37. L. Chen, A. B. Wolf, W. Fu, L. Li, J. M. Akey, Identifying and interpreting apparent Neanderthal ancestry in African individuals. *Cell* **180**, 677–687.e16 (2020).

38. J. Yang, L. N. M. Iasi, Q. Fu, N. P. Cooke, J. Kelso, S. Peyrégne, B. M. Peter, An early East Asian lineage with unexpectedly low Denisovan ancestry. *Curr. Biol.* **35**, 4898–4908.e4 (2025).

39. A. Fujimoto, R. Kimura, J. Ohashi, K. Omi, R. Yuliwulandari, L. Batubara, M. S. Mustofa, U. Samakkarn, W. Settheetham-ishida, T. Ishida, Y. Morishita, T. Furusawa, M. Nakazawa, R. Ohtsuka, K. Tokunaga, A scan for genetic determinants of human hair morphology: EDAR is associated with Asian hair thickness. *Hum. Mol. Genet.* **17**, 835–843 (2008).

40. R. Kimura, T. Yamaguchi, M. Takeda, O. Kondo, T. Toma, K. Haneji, T. Hanihara, H. Matsukusa, S. Kawamura, K. Maki, M. Osawa, H. Ishida, H. Oota, A common variation in EDAR Is a genetic determinant of shovel-shaped incisors. *Am. J. Hum. Genet.* **85**, 528–535 (2009).

41. H. J. Edenberg, W. F. Bosron, "Alcohol dehydrogenases," in *Biotransformation*, F. P. Guengreich, Ed., vol. 3 of *Comprehensive Toxicology*, I. G. Sipes, C. A. McQueen, A. J. Gandolfi, Eds. (Pergamon, 1997), pp. 119–131.

42. S. Harada, D. P. Agarwal, H. W. Goedde, Aldehyde dehydrogenase deficiency as cause of facial flushing reaction to alcohol in japanese. *Lancet* **318**, 982 (1981).

43. Z. Yang, H. Zhong, J. Chen, X. Zhang, H. Zhang, X. Luo, S. Xu, H. Chen, D. Lu, Y. Han, J. Li, L. Fu, X. Qi, Y. Peng, K. Xiang, Q. Lin, Y. Guo, M. Li, X. Cao, Y. Zhang, S. Liao, Y. Peng, L. Zhang, X. Guo, S. Dong, F. Liang, J. Wang, A. Willden, H. Seang Aun, B. Serey, T. Sovannary, L. Bunnath, H. Samnom, G. Mardon, Q. Li, A. Meng, H. Shi, B. Su, A genetic mechanism for convergent skin lightening during recent human evolution. *Mol. Biol. Evol.* **33**, 1177–1187 (2016).

44. L. Rawofi, M. Edwards, S. Krithika, P. Le, D. Cha, Z. Yang, Y. Ma, J. Wang, B. Su, L. Jin, H. L. Norton, E. J. Parra, Genome-wide association study of pigmentary traits (skin and iris color) in individuals of East Asian ancestry. *PeerJ* **5**, e3951 (2017).

45. K. Yamaguchi, C. Watanabe, A. Kawaguchi, T. Sato, I. Naka, M. Shindo, K. Moromizato, K. Aoki, H. Ishida, R. Kimura, Association of melanocortin 1 receptor gene (MC1R) polymorphisms with skin reflectance and freckles in Japanese. *J. Hum. Genet.* **57**, 700–708 (2012).

46. K. I. Yoshiura, A. Kinoshita, T. Ishida, A. Ninokata, T. Ishikawa, T. Kaname, M. Bannai, K. Tokunaga, S. Sonoda, R. Komaki, M. Ihara, V. A. Saenko, G. K. Alipov, I. Sekine, K. Komatsu, H. Takahashi, M. Nakashima, N. Sosonkina, C. K. Mapendano, M. Ghadami, M. Nomura, D. S. Liang, N. Miwa, D. K. Kim, A. Garidkhuu, N. Natsume, T. Ohta, H. Tomita, A. Kaneko, M. Kikuchi, G. Russomando, K. Hirayama, M. Ishibashi, A. Takahashi, N. Saitou, J. C. Murray, S. Saito, Y. Nakamura, N. Niikawa, A SNP in the ABCC11 gene is the determinant of human earwax type. *Nat. Genet.* **38**, 324–330 (2006).

47. A. M. Hancock, V. J. Clark, Y. Qian, A. Di Rienzo, Population genetic analysis of the uncoupling proteins supports a role for UCP3 in human cold resistance. *Mol. Biol. Evol.* **28**, 601–614 (2011).

48. T. Nishimura, T. Katsumura, M. Motoi, H. Oota, S. Watanuki, Experimental evidence reveals the UCP1 genotype changes the oxygen consumption attributed to non-shivering thermogenesis in humans. *Sci. Rep.* **7**, 5570 (2017).

49. F. M. Key, M. A. Abdul-Aziz, R. Mundry, B. M. Peter, A. Sekar, M. D'Amato, M. Y. Dennis, J. M. Schmidt, A. M. Andrés, Human local adaptation of the TRPM8 cold receptor along a latitudinal cline. *PLOS Genet.* **14**, e1007298 (2018).

50. Z. A. Szpiech, R. D. Hernandez, Selscan: An efficient multithreaded program to perform EHH-based scans for positive selection. *Mol. Biol. Evol.* **31**, 2824–2827 (2014).

51. M. Akiyama, Y. Okada, M. Kanai, A. Takahashi, Y. Momozawa, M. Ikeda, N. Iwata, S. Ikegawa, M. Hirata, K. Matsuda, M. Iwasaki, T. Yamaji, N. Sawada, T. Hachiya, K. Tanno, A. Shimizu, A. Hozawa, N. Minegishi, S. Tsugane, M. Yamamoto, M. Kubo, Y. Kamatani, Genome-wide association study identifies 112 new loci for body mass index in the Japanese population. *Nat. Genet.* **49**, 1458–1467 (2017).

52. M. Kanai, M. Akiyama, A. Takahashi, N. Matoba, Y. Momozawa, M. Ikeda, N. Iwata, S. Ikegawa, M. Hirata, K. Matsuda, M. Kubo, Y. Okada, Y. Kamatani, Genetic analysis of quantitative traits in the Japanese population links cell types to complex human diseases. *Nat. Genet.* **50**, 390–400 (2018).

53. C. Y. Chen, T. T. Chen, Y. C. A. Feng, M. Yu, S. C. Lin, R. J. Longchamps, S. H. Wang, Y. H. Hsu, H. I. Yang, P. H. Kuo, M. J. Daly, W. J. Chen, H. Huang, T. Ge, Y. F. Lin, Analysis across Taiwan Biobank, Biobank Japan, and UK Biobank identifies hundreds of novel loci for 36 quantitative traits. *Cell Genom.* **3**, 100436 (2023).

54. Z. Zhang, N. Chen, N. Yin, R. Liu, Y. He, D. Li, M. Tong, A. Gao, P. Lu, Y. Zhao, H. Li, J. Zhang, D. Zhang, W. Gu, J. Hong, W. Wang, L. Qi, G. Ning, J. Wang, The rs1421085 variant within FTO promotes brown fat thermogenesis. *Nat. Metab.* **5**, 1337–1351 (2023).

55. X. Y. Zheng, B. L. Yu, Y. F. Xie, S. P. Zhao, C. L. Wu, Apolipoprotein A5 regulates intracellular triglyceride metabolism in adipocytes. *Mol. Med. Rep.* **16**, 6771–6779 (2017).

56. M. Fumagalli, I. Moltke, N. Grarup, F. Racimo, P. Bjerregaard, M. E. Jørgensen, T. S. Korneliussen, P. Gerbault, L. Skotte, A. Linneberg, C. Christensen, I. Brandslund, T. Jørgensen, E. Huerta-Sánchez, E. B. Schmidt, O. Pedersen, T. Hansen, A. Albrechtsen, R. Nielsen, Greenlandic Inuit show genetic signatures of diet and climate adaptation. *Science* **349**, 1343–1347 (2015).

57. O. E. Craig, H. Saul, A. Lucquin, Y. Nishida, K. Taché, L. Clarke, A. Thompson, D. T. Altoft, J. Uchiyama, M. Ajimoto, K. Gibbs, S. Isaksson, C. P. Heron, P. Jordan, Earliest evidence for the use of pottery. *Nature* **496**, 351–354 (2013).

58. J. V. Moreno-Mayar, B. A. Potter, L. Vinner, M. Steinrücken, S. Rasmussen, J. Terhorst, J. A. Kamm, A. Albrechtsen, A.-S. Malaspinas, M. Sikora, J. D. Reuther, J. D. Irish, R. S. Malhi, L. Orlando, Y. S. Song, R. Nielsen, D. J. Meltzer, E. Willerslev, Terminal Pleistocene Alaskan genome reveals first founding population of Native Americans. *Nature* **553**, 203–207 (2018).

59. V. J. Moreno-Mayar, L. Vinner, P. de Barros Damgaard, C. de la Fuente, J. Chan, J. P. Spence, M. E. Allentoft, T. Vimala, F. Racimo, T. Pinotti, S. Rasmussen, A. Margaryan, M. I. Orbegozo, D. Mylopotamitaki, M. Wooller, C. Bataille, L. Becerra-Valdivia, D. Chivall, D. Comeskey, T. Devièse, D. K. Grayson, L. George, H. Harry, V. Alexandersen, C. Primeau, J. Erlandson, C. Rodrigues-Carvalho, S. Reis, M. Q. R. Bastos, J. Cybulski, C. Vullo, F. Morello, M. Vilar, S. Wells, K. Gregersen, K. L. Hansen, N. Lynnerup, M. M. Lahr, K. Kjær, A. Strauss, M. Alfonso-Durruty, A. Salas, H. Schroeder, T. Higham, R. S. Malhi, J. T. Rasic, L. Souza, F. R. Santos, A. S. Malaspinas, M. Sikora, R. Nielsen, Y. S. Song, D. J. Meltzer, E. Willerslev, Early human dispersals within the Americas. *Science* **362**, eaav2621 (2018).

60. Y. Watanabe, J. Ohashi, Modern Japanese ancestry-derived variants reveal the formation process of the current Japanese regional gradations. *iScience* **26**, 106130 (2023).

61. K. Yamamoto, S. Namba, K. Sonehara, K. Suzuki, S. Sakaue, N. P. Cooke, S. Higashiue, S. Kobayashi, H. Afuso, K. Matsuura, Y. Mitsumoto, Y. Fujita, T. Tokuda, K. Matsuda, T. Gakuhari, T. Yamauchi, T. Kadowaki, S. Nakagome, Y. Okada, Genetic legacy of ancient hunter-gatherer Jomon in Japanese populations. *Nat. Commun.* **15**, 9780 (2024).

62. J. V. Neel, Diabetes mellitus: A “thrifty” genotype rendered detrimental by “progress”? *Am. J. Hum. Genet.* **14**, 353–362 (1962).

63. M. J. Palma-Martínez, Y. S. Posadas-García, A. Shaukat, B. E. López-Ángeles, M. Sohail, Evolution, genetic diversity, and health. *Nat. Med.* **31**, 751–761 (2025).

64. N. Matsumoto, J. Habu, A. Matsui, “Subsistence, sedentism, and social complexity among Jomon hunter-gatherers of the Japanese archipelago,” in *Handbook of East and Southeast Asian Archaeology*, J. Habu, P. V Lape, J. W. Olsen, Eds. (Springer, 2017), pp. 437–450.

65. Y. Nakamura, D. Waku, Y. Wakiyama, Y. Watanabe, K. Koganebuchi, T. Nagaoka, K. Hirata, J. Ohashi, R. Takahashi, M. Yoneda, H. Oota, Collagen of ancient bones gives an indication of endogenous DNA preservation based on next-generation sequencing technology. *Anthropol. Sci.* **132**, 143–150 (2024).

66. Y. Yamada, Archaeological and anthropological views of Jomon society: Methods and practices. *Anthropol. Sci.* **130**, 3–13 (2022).

67. R. Longin, New method of collagen extraction for radiocarbon dating. *Nature* **230**, 241–242 (1971).

68. M. Yoneda, M. Hirota, M. Uchida, A. Tanaka, Y. Shibata, M. Morita, T. Akazawa, Radiocarbon and stable isotope analyses on the Earliest Jomon skeletons from the Tochibara rockshelter, Nagano, Japan. *Radiocarbon* **44**, 549–557 (2002).

69. P. J. Reimer, W. E. N. Austin, E. Bard, A. Bayliss, P. G. Blackwell, C. Bronk Ramsey, M. Butzin, H. Cheng, R. L. Edwards, M. Friedrich, P. M. Grootes, T. P. Guilderson, I. Hajdas, T. J. Heaton, A. G. Hogg, K. A. Hughen, B. Kromer, S. W. Manning, R. Muscheler, J. G. Palmer, C. Pearson, J. Van Der Plicht, R. W. Reimer, D. A. Richards, E. M. Scott, J. R. Southon, C. S. M. Turney, L. Wacker, F. Adolphi, U. Büntgen, M. Capano, S. M. Fahrni, A. Fogtmann-Schulz, R. Friedrich, P. Köhler, S. Kudsk, F. Miyake, J. Olsen, F. Reinig, M. Sakamoto, A. Sookdeo, S. Talamo, The IntCal20 Northern Hemisphere radiocarbon age calibration curve (0-55 cal kBP). *Radiocarbon* **62**, 725–757 (2020).

70. T. J. Heaton, P. Köhler, M. Butzin, E. Bard, R. W. Reimer, W. E. N. Austin, C. Bronk Ramsey, P. M. Grootes, K. A. Hughen, B. Kromer, P. J. Reimer, J. Adkins, A. Burke, M. S. Cook, J. Olsen, L. C. Skinner, Marine20 - The Marine radiocarbon age calibration curve (0-55,000 cal BP). *Radiocarbon* **62**, 779–820 (2020).

71. C. B. Ramsey, Bayesian analysis of radiocarbon dates. *Radiocarbon* **51**, 337–360 (2009).

72. K. Yoshida, T. Hara, D. Kunikita, Y. Miyazaki, T. Sasaki, M. Yoneda, H. Matsuzaki, Pre-bomb marine reservoir ages in the Western Pacific. *Radiocarbon* **52**, 1197–1206 (2010).

73. C. Gamba, E. R. Jones, M. D. Teasdale, R. L. McLaughlin, G. Gonzalez-Fortes, V. Mattiangeli, L. Domboróczki, I. Kovári, I. Pap, A. Anders, A. Whittle, J. Dani, P. Raczky, T. F. G. Higham, M. Hofreiter, D. G. Bradley, R. Pinhasi, Genome flux and stasis in a five millennium transect of European prehistory. *Nat. Commun.* **5**, 5257 (2014).

74. R. Pinhasi, D. Fernandes, K. Sirak, M. Novak, S. Connell, S. Alpaslan-Roodenberg, F. Gerritsen, V. Moiseyev, A. Gromov, P. Raczky, A. Anders, M. Pietrusewsky, G. Rollefson, M. Jovanovic, H. Trinhhoang, G. Bar-Oz, M. Oxenham, H. Matsumura, M. Hofreiter, Optimal ancient DNA yields from the inner ear part of the human petrous bone. *PLOS ONE* **10**, e0129102 (2015).

75. P. B. Damgaard, A. Margaryan, H. Schroeder, L. Orlando, E. Willerslev, M. E. Allentoft, Improving access to endogenous DNA in ancient bones and teeth. *Sci. Rep.* **5**, 11184 (2015).

76. N. Rohland, E. Harney, S. Mallick, S. Nordenfelt, D. Reich, Partial uracil–DNA–glycosylase treatment for screening of ancient DNA. *Philos. Trans. R. Soc. B Biol. Sci.* **370**, 20130624 (2015).

77. Q. Fu, M. Hajdinjak, O. T. Moldovan, S. Constantin, S. Mallick, P. Skoglund, N. Patterson, N. Rohland, I. Lazaridis, B. Nickel, B. Viola, K. Prüfer, M. Meyer, J. Kelso, D. Reich, S. Pääbo, An early modern human from Romania with a recent Neanderthal ancestor. *Nature* **524**, 216–219 (2015).

78. P. Skoglund, J. Storå, A. Götherström, M. Jakobsson, Accurate sex identification of ancient human remains using DNA shotgun sequencing. *J. Archaeol. Sci.* **40**, 4477–4482 (2013).

79. H. Jónsson, A. Ginolhac, M. Schubert, P. L. F. Johnson, L. Orlando, MapDamage2.0: Fast approximate Bayesian estimates of ancient DNA damage parameters. *Bioinformatics* **29**, 1682–1684 (2013).

80. C. Ning, C. C. Wang, S. Gao, Y. Yang, X. Zhang, X. Wu, F. Zhang, Z. Nie, Y. Tang, M. Robbeets, J. Ma, J. Krause, Y. Cui, Ancient genomes reveal Yamnaya-related ancestry and a potential source of Indo-European speakers in Iron Age Tianshan. *Curr. Biol.* **29**, 2526–2532.e4 (2019).

81. R. M. Andrews, D. M. Turnbull, I. Kubacka, P. F. Chinnery, R. N. Lightowlers, N. Howell, Reanalysis and revision of the Cambridge reference sequence for human mitochondrial DNA. *Nat. Genet.* **23**, 147 (1999).

82. K. Ishiya, S. Ueda, MitoSuite: A graphical tool for human mitochondrial genome profiling in massive parallel sequencing. *PeerJ* **5**, e3406 (2017).

83. T. S. Korneliussen, A. Albrechtsen, R. Nielsen, ANGSD: Analysis of next generation sequencing data. *BMC Bioinformatics* **15**, 356 (2014).

84. H. Kanzawa-Kiriyama, T. A. Jinam, Y. Kawai, T. Sato, K. Hosomichi, A. Tajima, N. Adachi, H. Matsumura, K. Kryukov, N. Saitou, K. I. Shinoda, Late Jomon male and female genome sequences from the funadomari site in Hokkaido, Japan. *Anthropol. Sci.* **127**, 83–108 (2019).

85. C. C. Wang, H. Y. Yeh, A. N. Popov, H. Q. Zhang, H. Matsumura, K. Sirak, O. Cheronet, A. Kovalev, N. Rohland, A. M. Kim, S. Mallick, R. Bernardos, D. Tumen, J. Zhao, Y. C. Liu, J. Y. Liu, M. Mah, K. Wang, Z. Zhang, N. Adamski, N. Broomandkhoshbacht, K. Callan, F. Candilio, K. S. D. Carlson, B. J. Culleton, L. Eccles, S. Freilich, D. Keating, A. M. Lawson, K. Mandl, M. Michel, J. Oppenheimer, K. T. Özdoğan, K. Stewardson, S. Wen, S. Yan, F. Zalzala, R. Chuang, C. J. Huang, H. Looh, C. C. Shiung, Y. G. Nikitin, A. V. Tabarev, A. A. Tishkin, S. Lin, Z. Y. Sun, X. M. Wu, T. L. Yang, X. Hu, L. Chen, H. Du, J. Bayarsaikhan, E. Mijiddorj, D. Erdenebaatar, T. O. Iderkhangai, E. Myagmar, H. Kanzawa-Kiriyama, M.

Nishino, K.-I. Shinoda, O. A. Shubina, J. Guo, W. Cai, Q. Deng, L. Kang, D. Li, D. Li, R. Lin, Nini, R. Shrestha, L. X. Wang, L. Wei, G. Xie, H. Yao, M. Zhang, G. He, X. Yang, R. Hu, M. Robbeets, S. Schiffels, D. J. Kennett, L. Jin, H. Li, J. Krause, R. Pinhasi, D. Reich, Genomic insights into the formation of human populations in East Asia. *Nature* **591**, 413–419 (2021).

86. N. P. Cooke, V. Mattiangeli, L. M. Cassidy, K. Okazaki, C. A. Stokes, S. Onbe, S. Hatakeyama, K. Machida, K. Kasai, N. Tomioka, A. Matsumoto, M. Ito, Y. Kojima, D. G. Bradley, T. Gakuhari, S. Nakagome, Ancient genomics reveals tripartite origins of Japanese populations. *Sci. Adv.* **7**, eabh2419 (2021).

87. M. Schubert, S. Lindgreen, L. Orlando, AdapterRemoval v2: Rapid adapter trimming, identification, and read merging. *BMC. Res. Notes* **9**, 88 (2016).

88. G. Jun, M. K. Wing, G. R. Abecasis, H. M. Kang, An efficient and scalable analysis framework for variant extraction and refinement from population-scale DNA sequence data. *Genome Res.* **25**, 918–925 (2015).

89. H. Li, B. Handsaker, A. Wysoker, T. Fennell, J. Ruan, N. Homer, G. Marth, G. Abecasis, R. Durbin, The sequence alignment/map format and SAMtools. *Bioinformatics* **25**, 2078–2079 (2009).

90. S. Rubinacci, R. J. Hofmeister, B. Sousa da Mota, O. Delaneau, Imputation of low-coverage sequencing data from 150,119 UK Biobank genomes. *Nat. Genet.* **55**, 1088–1090 (2023).

91. Y. Kawai, Y. Watanabe, Y. Omae, R. Miyahara, S. S. Khor, E. Noiri, K. Kitajima, H. Shimanuki, H. Gatanaga, K. Hata, K. Hattori, A. Iida, H. Ishibashi-Ueda, T. Kaname, T. Kanto, R. Matsumura, K. Miyo, M. Noguchi, K. Ozaki, M. Sugiyama, A. Takahashi, H. Tokuda, T. Tomita, A. Umezawa, H. Watanabe, S. Yoshida, Y. I. Goto, Y. Maruoka, Y. Matsubara, S. Niida, M. Mizokami, K. Tokunaga, Exploring the genetic diversity of the Japanese population: Insights from a large-scale whole genome sequencing analysis. *PLOS Genet.* **19**, e1010625 (2023).

92. The 1000 Genomes Project Consortium, A. Auton, L. D. Brooks, R. M. Durbin, E. P. Garrison, H. Min Kang, J. O. Korbel, J. L. Marchini, S. McCarthy, G. A. McVean, G. R. Abecasis, A global reference for human genetic variation. *Nature* **526**, 68–74 (2015).

93. S. Purcell, B. Neale, K. Todd-Brown, L. Thomas, M. A. R. Ferreira, D. Bender, J. Maller, P. Sklar, P. I. W. De Bakker, M. J. Daly, P. C. Sham, PLINK: A tool set for whole-genome association and population-based linkage analyses. *Am. J. Hum. Genet.* **81**, 559–575 (2007).

94. D. H. Alexander, J. Novembre, K. Lange, Fast model-based estimation of ancestry in unrelated individuals. *Genome Res.* **19**, 1655–1664 (2009).

95. J. K. Pickrell, J. K. Pritchard, Inference of population splits and mixtures from genome-wide allele frequency data. *PLOS Genet.* **8**, e1002967 (2012).

96. S. Mallick, H. Li, M. Lipson, I. Mathieson, M. Gymrek, F. Racimo, M. Zhao, N. Chennagiri, S. Nordenfelt, A. Tandon, P. Skoglund, I. Lazaridis, S. Sankararaman, Q. Fu, N. Rohland, G. Renaud, Y. Erlich, T. Willems, C. Gallo, J. P. Spence, Y. S. Song, G. Poletti, F. Balloux, G. van Driem, P. de Knijff, I. G. Romero, A. R. Jha, D. M. Behar, C. M. Bravi, C. Capelli, T. Hervig, A. Moreno-Estrada, O. L. Posukh, E. Balanovska, O. Balanovsky, S. Karachanak-Yankova, H. Sahakyan, D. Toncheva, L. Yepiskoposyan, C. Tyler-Smith, Y. Xue, M. S. Abdullah, A. Ruiz-Linares, C. M. Beall, A. Di Rienzo, C. Jeong, E. B. Starikovskaya, E. Metspalu, J. Parik, R. Villems, B. M. Henn, U. Hodoglugil, R. Mahley, A. Sajantila, G. Stamatoyannopoulos, J. T. S. Wee, R. Khusainova, E. Khusnutdinova, S. Litvinov, G. Ayodo, D. Comas, M. F. Hammer, T. Kivisild, W. Klitz, C. A. Winkler, D. Labuda, M. Bamshad, L. B. Jorde, S. A. Tishkoff, W. S. Watkins, M. Metspalu, S. Dryomov, R. Sukernik, L. Singh, K. Thangaraj, S. Pääbo, J. Kelso, N. Patterson, D. Reich, The Simons Genome Diversity Project: 300 genomes from 142 diverse populations. *Nature* **538**, 201–206 (2016).

97. M. Mondal, F. Casals, T. Xu, G. M. Dall'Olio, M. Pybus, M. G. Netea, D. Comas, H. Laayouni, Q. Li, P. P. Majumder, J. Bertranpetit, Genomic analysis of Andamanese provides insights into ancient human migration into Asia and adaptation. *Nat. Genet.* **48**, 1066–1070 (2016).

98. R. Maier, P. Flegontov, O. Flegontova, U. Işıldak, P. Changmai, D. Reich, On the limits of fitting complex models of population history to *f*-statistics. *eLife* **12**, e85492 (2023).

99. A. Bergström, S. A. McCarthy, R. Hui, M. A. Almarri, Q. Ayub, P. Danecek, Y. Chen, S. Felkel, P. Hallast, J. Kamm, H. Blanché, J. F. Deleuze, H. Cann, S. Mallick, D. Reich, M. S. Sandhu, P. Skoglund, A. Scally, Y. Xue, R. Durbin, C. Tyler-Smith, Insights into human genetic variation and population history from 929 diverse genomes. *Science* **367**, eaay5012 (2020).

100. W. Zhang, J. Meehan, Z. Su, H. W. Ng, M. Shu, H. Luo, W. Ge, R. Perkins, W. Tong, H. Hong, Whole genome sequencing of 35 individuals provides insights into the genetic architecture of Korean population. *BMC Bioinformatics* **15**, S6 (2014).

101. M. Meyer, M. Kircher, M. T. Gansauge, H. Li, F. Racimo, S. Mallick, J. G. Schraiber, F. Jay, K. Prüfer, C. De Filippo, P. H. Sudmant, C. Alkan, Q. Fu, R. Do, N. Rohland, A. Tandon, M. Siebauer, R. E. Green, K. Bryc, A. W. Briggs, U. Stenzel, J. Dabney, J. Shendure, J. Kitzman, M. F. Hammer, M. V. Shunkov, A. P. Derevianko, N. Patterson, A. M. Andrés, E. E. Eichler, M. Slatkin, D. Reich, J. Kelso, S. Pääbo, A high-coverage genome sequence from an archaic Denisovan individual. *Science* **338**, 222–226 (2012).

102. K. Prüfer, F. Racimo, N. Patterson, F. Jay, S. Sankararaman, S. Sawyer, A. Heinze, G. Renaud, P. H. Sudmant, C. de Filippo, H. Li, S. Mallick, M. Dannemann, Q. Fu, M. Kircher, M. Kuhlwilm, M. Lachmann, M. Meyer, M. Ongyerth, M. Siebauer, C. Theunert, A. Tandon, P. Moorjani, J. Pickrell, J. C. Mullikin, S. H. Vohr, R. E. Green, I. Hellmann, P. L. F. Johnson, H. Blanche, H. Cann, J. O. Kitzman, J. Shendure, E. E. Eichler, E. S. Lein, T. E. Bakken, L. V. Golovanova, V. B. Doronichev, M. V. Shunkov, A. P. Derevianko, B. Viola, M. Slatkin, D. Reich, J. Kelso, S. Pääbo, The complete genome sequence of a Neanderthal from the Altai Mountains. *Nature* **505**, 43–49 (2014).

103. R. M. Kuhn, D. Haussler, W. James Kent, The UCSC genome browser and associated tools. *Brief. Bioinform.* **14**, 144–161 (2013).

104. C. Endo, T. A. Johnson, R. Morino, K. Nakazono, S. Kamitsuji, M. Akita, M. Kawajiri, T. Yamasaki, A. Kami, Y. Hoshi, A. Tada, K. Ishikawa, M. Hine, M. Kobayashi, N. Kurume, Y.

Tsunemi, N. Kamatani, M. Kawashima, Genome-wide association study in Japanese females identifies fifteen novel skin-related trait associations. *Sci. Rep.* **8**, 8974 (2018).

105. M. Akiyama, K. Ishigaki, S. Sakaue, Y. Momozawa, M. Horikoshi, M. Hirata, K. Matsuda, S. Ikegawa, A. Takahashi, M. Kanai, S. Suzuki, D. Matsui, M. Naito, T. Yamaji, M. Iwasaki, N. Sawada, K. Tanno, M. Sasaki, A. Hozawa, N. Minegishi, K. Wakai, S. Tsugane, A. Shimizu, M. Yamamoto, Y. Okada, Y. Murakami, M. Kubo, Y. Kamatani, Characterizing rare and low-frequency height-associated variants in the Japanese population. *Nat. Commun.* **10**, 4393 (2019).

106. N. Patterson, P. Moorjani, Y. Luo, S. Mallick, N. Rohland, Y. Zhan, T. Genschoreck, T. Webster, D. Reich, Ancient admixture in human history. *Genetics* **192**, 1065–1093 (2012).

107. J. W. Leigh, D. Bryant, POPART: Full-feature software for haplotype network construction. *Methods Ecol. Evol.* **6**, 1110–1116 (2015).

108. A. J. Stern, P. R. Wilton, R. Nielsen, An approximate full-likelihood method for inferring selection and allele frequency trajectories from DNA sequence data. *PLOS Genet.* **15**, e1008384 (2019).

109. A. H. Vaughn, R. Nielsen, Fast and accurate estimation of selection coefficients and allele histories from ancient and modern DNA. *Mol. Biol. Evol.* **41**, msae156 (2024).

110. K. Morisaki, *Kyusekki Shakai No Jinrui Seitaigaku* (Douseisha Publishing, 2022).

111. L. R. Binford, Willow smoke and dogs' tails: Hunter-gatherer settlement systems and archaeological site formation. *Am. Antiq.* **45**, 4–20 (1980).

112. C. B. Ramsey, Methods for summarizing radiocarbon datasets. *Radiocarbon* **59**, 1809–1833 (2017).

113. K. Shichi, T. Goebel, M. Izuho, K. Kashiwaya, Climate amelioration, abrupt vegetation recovery, and the dispersal of *Homo sapiens* in Baikal Siberia. *Sci. Adv.* **9**, eadi0189 (2023).

114. A. Iwase, J. Hashizume, M. Izuho, K. Takahashi, H. Sato, Timing of megafaunal extinction in the late Late Pleistocene on the Japanese Archipelago. *Quat. Int.* **255**, 114–124 (2012).

115. H. Takahara, R. Hayashi, "Paleovegetation during marine isotope Stage 3 in East Asia," in *Emergence and Diversity of Modern Human Behavior in Palaeolithic Asia*, Y. Kaifu, M. Izuho, T. Goebel, H. Sato, A. Ono, Eds. (Texas A&M Univ. Press, 2015), pp. 314–324.

116. G. Jeong, H. Gill, H. Moon, C. Jeong, An ancient genome perspective on the dynamic history of the prehistoric Jomon people in and around the Japanese archipelago. *Hum. Popul. Genet. Genom.* **3**, 0008 (2023).

117. M. A. Yang, X. Gao, C. Theunert, H. Tong, A. Aximu-Petri, B. Nickel, M. Slatkin, M. Meyer, S. Pääbo, J. Kelso, Q. Fu, 40,000-year-old individual from Asia provides insight into early population structure in Eurasia. *Curr. Biol.* **27**, 3202–3208.e9 (2017).

118. T. Wang, M. A. Yang, Z. Zhu, M. Ma, H. Shi, L. Speidel, R. Min, H. Yuan, Z. Jiang, C. Hu, X. Li, D. Zhao, F. Bai, P. Cao, F. Liu, Q. Dai, X. Feng, R. Yang, X. Wu, X. Liu, M. Zhang, W. Ping, Y. Liu, Y. Wan, F. Yang, R. Zhou, L. Kang, G. Dong, M. Stoneking, Q. Fu, Prehistoric genomes from Yunnan reveal ancestry related to Tibetans and Austroasiatic speakers. *Science* **388**, eadq9792 (2025).

119. L. G. Davis, D. B. Madsen, L. Becerra-Valdivia, T. Higham, D. A. Sisson, S. M. Skinner, D. Stueber, A. J. Nyers, A. Keen-Zebert, C. Neudorf, M. Cheyney, M. Izuho, F. Iizuka, S. R. Burns, C. W. Epps, S. C. Willis, I. Buvit, Late Upper Paleolithic occupation at Cooper's Ferry, Idaho, USA, ∼16,000 years ago. *Science* **365**, 891–897 (2019).

120. L. G. Davis, D. B. Madsen, D. A. Sisson, L. Becerra-Valdivia, T. Higham, D. Stueber, D. W. Bean, A. J. Nyers, A. Carroll, C. Ryder, M. Sponheimer, M. Izuho, F. Iizuka, G. Li, C. W. Epps, F. K. Halford, Dating of a large tool assemblage at the Cooper's Ferry site (Idaho, USA) to ∼15,785 cal yr B.P. extends the age of stemmed points in the Americas. *Sci. Adv.* **8**, eade1248 (2022).

121. L. G. Davis, D. B. Madsen, Exploring potential cultural connections between the Late Pleistocene Peoples of Japan and North America. *Anthropol. Pap. Univ. Alaska* **7**, 96–112 (2023).

122. M. R. Waters, Late Pleistocene exploration and settlement of the Americas by modern humans. *Science* **365**, eaat5447 (2019).
